# Aloperine alleviates LPS-induced inflammation in bovine intestinal epithelial cells through autophagy and TLR4/p38 MAPK/NF-κB pathway

**DOI:** 10.1186/s12917-026-05337-7

**Published:** 2026-02-20

**Authors:** Panpan Tan, Yazhou Wang, Cai Zhang, Qinghua Deng, Liyin Du, Baoyu Zhao, Gulman Muhametbay, Chenxu Zhao, Jianguo Wang

**Affiliations:** 1 College of Animal Science and Technology, Inner Mongolia Minzu University, Tongliao, 028000 China; 2 College of Veterinary Medicine, Northwest A&F University, Yangling, Shaanxi 712100 China; 3 Inner Mongolia Engineering Technology Research Center of Prevention and Control the Beef Cattle Disease, Tongliao, 028000 China; 4 Beef Cattle Industry School of Inner Mongolia, Tongliao, 028000 China; 5 College of Animal Science and Technology, Henan University of Science and Technology, Luoyang, 471023 China; 6 Animal Disease Prevention and Control Center of Xinjiang, Urumqi, 830000 China

**Keywords:** Calf diarrhea, Aloperine, Inflammation, Autophagy, Network pharmacology

## Abstract

**Background:**

Calf diarrhea is a major cause of mortality and morbidity, leading to substantial economic losses in the cattle industry. Aloperine (Alo) exhibits an anti-inflammation effect and alleviates dextran sulfate sodium salt (DSS)-induced colitis; however, it remains unclear whether Alo alleviates calf diarrhea-induced intestinal inflammation.

**Results:**

In this study, network pharmacology was used to discover the possible mechanism of Alo on the anti-inflammatory effect; Then, an inflammation model was induced by LPS in BIECs-21 to evaluate the protective effect of Alo on LPS-induced inflammation. Results found that a total of 68 overlapping targets of Alo and inflammation were obtained, among which ALB, AKT1, IL-6, and EGFR exhibited good affinity for Alo. In vitro experiments, Alo inhibited LPS-induced pro-inflammatory cytokine levels, increased the expression of ZO-1 and Claudin 1, and reduced the expression of proteins related to autophagy and TLR4/p38 MAPK/NF-κB pathway; the autophagy inhibitor CQ and Baf A1 results further demonstrated that Alo inhibited late stages of autophagy maturation and impaired intestinal epithelial barrier function.

**Conclusions:**

These results indicated that Alo has protective effects on LPS-induced inflammation by decreasing the levels of the pro-inflammatory cytokines through the TLR4/p38 MAPK/NF-κB pathway, inhibiting late stages of autophagy maturation, and impairing intestinal epithelial barrier function.

**Supplementary Information:**

The online version contains supplementary material available at 10.1186/s12917-026-05337-7.

## Background

Diarrhea is one of the most common diseases in dairy calfs, accounting for more than 50% of calf mortality, leading to malnutrition, digestive dysfunction, and reduced growth and production performance, resulting in significant economic losses [1–3]. Calf diarrhea is predominantly caused by various infectious agents (viruses, parasites, bacteria, and enteric pathogens), among which *Escherichia coli* (ETEC) is a primary bacterial cause of diarrhea in newborn calfs [4, 5]. Currently, the prevention and treatment of calf diarrhea primarily rely on antimicrobials [4]; However, the overuse of antimicrobials leads to the development and spread of antimicrobial resistance and drug residues [5–7]. Therefore, it is essential to develop safe and effective alternative treatments for diarrhea.

Aloperine (Alo) is a quinolizidine alkaloid derived from the *Sophora Alopecuroides* plant, a traditional Chinese medicine [8]. Alo has shown various pharmacological effects, including anti-neoplastic, antioxidant, anti-microbial, antiviral, and anti-allergic effects, and antipyretic [9–11]. Alo has been found to inhibit the fusion of autophagosomes with lysosomes and autophagic flux, leading to tumor cell apoptosis and suppressing tumor growth [12, 13]. Additionally, Alo exhibits anti-inflammation effect by blocking the activation of the TLR4/MyD88/NF-κB pathway, reducing ROS production, and inhibiting the NLRP3 inflammasome activation [14–16]. Research suggested that Alo can alleviate DSS-induced colitis through PP2A-mediated PI3K/AKT/mTOR signaling [17]. Diarrhea is often accompanied by enteritis, while it remains unclear whether Alo can exert anti-inflammatory activity to alleviate diarrhea.

To evaluate the therapeutic and preventive effect of Alo on calf diarrhea, in the present study, the network pharmacology method was used to predict potential targets of Alo and its hub genes involved in the treatment of inflammation, and an inflammation model in bovine intestinal epithelial cells to used to validate the underlying mechanism as predicted.

## Materials and methods

### Chemicals and reagents

Aloperine (Alo) was purchased from Shanghai Yuanye Biological Technology Co., Ltd. (purity: >98%, Shanghai, China); LPS (*E. coli* O111:B4, S1732) was from Beyotime Biotechnology (Shanghai, China); bovine IL-1β (MM-36949O1), IL-6 (MM-34736O1), TNF-α (MM-1568O1) ELISA kits were purchased from Jiangsu Meimian industrial Co., Ltd (Yancheng, China); Cell Counting Kit (CCK8) was purchased from ZETA life (USA); p-p65 (AF2006), p65 (AF5006) and IKKβ (AF6009) were from Affinity Biosciences (Jiangsu, China), p-IκB (9246 S), IκB (4814 S), p-p38 (8690 S), p38 (4511 S) and LC3B (#2775) were from Cell Signaling Technology (Danvers, MA, USA); TLR4 (ab217274) was from Abcam (Cambridge, the United Kingdom); MyD88 (A0980) was from Abclonal Technology (Wuhan, China); p62 (AF0279) and LAMP2A (AF1036) were from Beyotime Biotechnology (Shanghai, China); ZO-1 (UC280753) and Occludin (TK276503A) were from Thermo Fisher (Waltham, MA, USA); Claudin 1 (CY6872) was from Abway (Shanghai, China); Bafilomycin A1 (Baf A1, HY-100558) and chloroquine (CQ, HY-17589 A) were purchased from MCE (Shanghai, China).

### Potential target proteins of Alo and diarrhea

The potential targets of Alo were searched from SwissTargetPrediction databases (http://swisstargetprediction.ch/), PharmMapper (http://www.lilab-ecust.cn/pharmmapper/), Herb (http://herb.ac.cn/) database.

The disease target information related to inflammation was searched from web databases, including TCMSP (https://old.tcmsp-e.com/tcmsp.php), Therapeutic Target Database (https://db.idrblab.net/ttd/), DisGeNET database (https://www.disgenet.org/home/), Online Mendelian Inheritance in Man (OMIM) database (https://www.omim.org/), and Genecard database (https://www.genecards.org/). After combining these targets, the redundancy was removed and was regarded as the related targets of inflammation.

Through the UniProt database (https://www.uniprot.org/), the target was screened and limited to the species named “*Bos taurus*”, which was standardized to the target genes. The VENNY 2.1 (https://bioinfogp.cnb.csic.es/tools/venny/) was used to obtain the overlapping targets of Alo and inflammatory.

### Gene ontology and KEGG pathway enrichment analyses

The obtained intersection targets of Alo and inflammatory were imported into the Omicshare Database (https://www.omicshare.com/tools/home/index/index.html) for GO and pathway enrichment analysis. The enrichment was conducted with the “*Bos taurus*” condition, and a threshold BH-corrected *P-value* (*q-value*) < 0.05, and the top 20 GO enrichment and pathways with count values were selected. The visualization analysis was carried out to make bubble chart and histogram.

### Protein-protein interaction network construction and hub genes analysis

To further elucidate the potential mechanism underlying Alo’s anti-inflammatory effect, these overlapping targets were used to construct a protein-protein interaction (PPI) network on the Search Tool for the Retrieval of Interacting Genes/Proteins (STRING) database (https://string-db.org/) with the “*Bos taurus*” setting. Cytoscape 3.7.0 was used to visualize the PPI network, and the plug-in “Network Analysis” was used to visualize the topological properties of each node in the network. The hub genes were screened out based on the conditions of degree, betweenness, and closeness.

### Molecular docking

The molecular structure of Alo (*Mol2 format) was downloaded from the PubChem Compound database (https://pubchem.ncbi.nlm.nih.gov/). The 3D structure of the target protein was downloaded from the Protein Data Bank (PDB) database (https://www.rcsb.org/). After removing solvent and heteromolecules, hydrogen and Kollman charges were added to the receptor; Then, molecular docking was performed by AutoDock Vina (1.5.7) to select the optimal result with the lowest binding capacity, and the online platform Proteins Plus (https://proteins.plus/) were analyzed their interactions and 2D interaction maps were obtained, and the results were visualized with PYMOL-Open-Source (2.4) software. The intermolecular force was analysis by PLIP web tool (https://plip-tool.biotec.tu-dresden.de/plip-web/plip/index). The binding ability of ligands and receptors is evaluated by the binding energy. If the binding energy is less than 0, it means that the ligand and receptor can spontaneously bind, and the smaller the value, the higher the binding activity.

### Cell culture and drug treatment

Bovine intestinal epithelial cells (BIECs-21) were a gift from Dr. Cai Zhang (Henan University of Science and Technology) [18]. Cells were cultured in DMEM (high glucose) supplemented with 10% fetal bovine serum (Cegrogen Biotech GmbH, Germany) and 100 IU/mL streptomycin-penicillin at 37 °C with 5% CO_2_ in a humidified incubator.

BIECs-21 cells were pre-treated with CQ (solvent: DMSO, 10 µmmol/L), or Baf A1 (solvent: DMSO, 100 nmmol/L) for 6 h, then added LPS (solvent: PBS buffer) or Alo (solvent: sterile water). In the control group, add an equal volume of fresh medium. Incubate the cells at 37 °C for an additional 6 h. Finally, the cells were collected for subsequent experiments. When used as a solvent, DMSO should have a final concentration of less than 0.1%. Each experiment was repeated three times.

### Cell viability

BIECs-21 cells were seeded into 96-well plates at a density of 5000 cell/well and grown until 80–90% confluent. Subsequently, DMEM containing 10% FBS with different concentrations of LPS (0, 1, 3, 5, and 10 µg/mL) for 12 h or Alo (0, 10, 25, 50, 75, 100, 200, and 400 µmol/L) for 12 h was replaced. Then, 10 µL of CCK-8 solution was added to each well, and the absorbance was measured at 450 nm with a multimode microplate reader after 4 h of incubation (BioTek, Winooski, VT, USA). The cell viability was calculated using the following formula: cell viability = (OD_drug_ - OD_blank_) / (OD_control_ - OD_blank_) × 100%. The experiment for each dose was given six times.

### RT-PCR

Total RNA was extracted from the treated cells with TRIzol reagent and reverse-transcribed into cDNA with PrimeScript II 1st Strand cDNA Synthesis Kit (KR116, TIANGEN, Beijing, China). Amplification and quantification were measured by a real-time PCR system (BIO-RAD S1000, USA) with SYBR qPCR Master Mix (Q311, Vazyme, Nanjing, China). 18 S and GAPDH were chosen as internal normalization controls. The relative mRNA expression of genes was calculated using the 2^−ΔΔCt^ method. All primers were designed to span introns (Table 1). Three replicates were performed in each sample. PCR was performed under the following conditions: 95 °C for 15 s and 40 cycles of amplification (95 °C for 5 s, 60 °C for 10 s, and 72 °C for 15 s), then Melt curve (65–95 °C).

**Table 1:** Sequences of the primers

Genes	Primer sequence (5’ → 3’)	Product size (bp)	Genbank
GAPDH	F: CCTGCCAAGTATGATGAGAT; R: AGTGTCGCTGTTGAAGTC	117	NM_001034034
18S	F: ACCCATTCGAACGTCTGCCCTATT; R: TCCTTGGATGTGGTAGCCGTTTCT	130	NR_036642.1
IL-1β	F: TCTTCGAAACGTCCTCCGAC; R: GCTCATGCAGAACACCACTTC	170	NM_174093.1
IL-6	F: AACGAGTGGGTAAAGAACGC; R: CTGACCAGAGGAGGGAATGC	144	NW_00310889.1
TNF-α	F:TCTCTCTCACATACCCTGCCA; R: ACCTGGGGACTGCTCTTCC	270	NM_173966.3

### ELISA

Cytokine concentrations in the cell supernatant were examined using ELISA kits according to the manufacturer’s instructions for IL-1β, IL-6, and TNF-α. The reference standard was used on an ELISA plate to make a standard curve. Each well was filled with 50 µL of standard or diluted cell supernatant samples (1:4). After sealing the plate, it was incubated in the dark for 30 min at 37 °C. Following five washes, each well was incubated with 50 µL of streptavidin-HRP (except the blank well) in the dark for 30 min at 37 °C; After washed five times, 50 µL of 3, 3’, 5, 5’-tetramethylbenzidine (TMB) substrate reagents A and B were added to each well, respectively, and the plate was incubated for 10 min at 37 °C in the dark. The colour changed from blue to yellow when 50 µL of stop solution was added. Finally, the optical density was spectrophotometrically measured at 450 nm using a microplate reader.

### Western blot

The total protein was extracted from cells using RIPA lysis buffer (Strong) supplemented with PSMF and phosphatase inhibitor cocktail. The protein concentrations were measured using the BCA protein assay kit (Beyotime, Shanghai, China). Then, equal amounts of protein were separated by SDS-PAGE and transferred to PVDF membranes. The membranes were blocked with 5% skimmed milk powder and subsequently incubated overnight at 4 ℃ with primary antibodies against rabbit anti-p-p65 (1:2000), rabbit anti-p65 (1:2000), rabbit anti-IKKβ (1:2000), mouse anti-p-IκB (1:2000), mouse anti-IκB (1:2000), rabbit anti-p-p38 (1:2000), rabbit anti-p38(1:2000), rabbit anti-LC3B (1:1000), rabbit anti-TLR4 (1:1000), rabbit anti-MyD88 (1:3000), mouse anti-p62 (1:1000), rabbit anti-LAMP2A (1:2000), rabbit anti-ZO-1 (1:1000), rabbit anti-Occludin (1:1000), rabbit anti-Claudin 1 (1:2000), and mouse anti-β-actin (1:5000). Then membranes were incubated with the corresponding secondary antibody (1:5000) for 1 h at room temperature. A chemiluminescent solution was used to develop color under an illumination instrument. The blotted proteins were visualized after staining with the enhanced chemiluminescent (ECL) reagent. Protein band intensity was measured and quantified by ImageJ software. The blots were stripped and reprobed with an antibody against β-actin. Experiments were repeated 3 times. Intensity values were normalized relative to control values.

### Immunofluorescence assay

BIECs-21 cells were seeded into coverslips and grown until 80–90% confluent. The cell coverslips were stimulated with LPS (10 µg/mL) and treated with Alo extract for 6 h. Then the cell coverslips were washed, fixed, permeabilized, and incubated with rabbit anti-p-p65 antibody (1:500), mouse anti-p-IκB antibody (1:500), mouse anti-p62 antibody (1:500), rabbit anti-LAMP2A antibody (1:500), and rabbit anti-LC3B antibody (1:500) overnight at 4 ℃. The cells were incubated with appropriate fluorescence-conjugated secondary antibodies (1:800), followed by DAPI mounting (1:800). Finally, the anti-fluorescence quenching agent was used to seal the sections.

### Statistical analysis

Data are depicted as the mean ± SEM. The ANOVA test was used for comparisons between groups. *P* values < 0.05 were considered statistically significant. Statistical tests were performed using GraphPad Prism 8.0.

## Results

### Identification of targets for Alo and inflammation

**Fig. 1:**
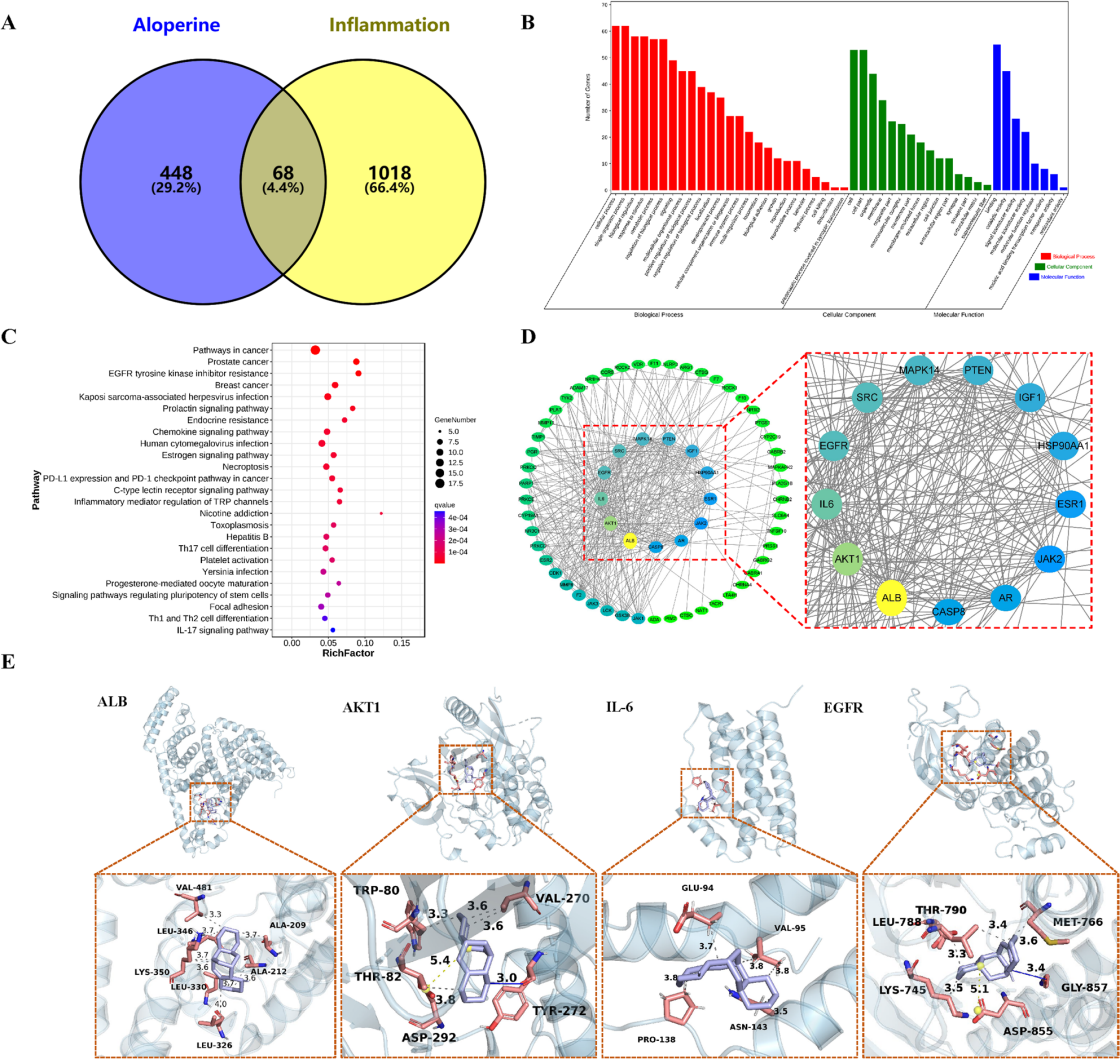
Network pharmacology and molecular docking analysis of Alo on inflammation. **A** Venny of Alo and inflammation. **B** GO enrichment analysis of common target for Alo and inflammation; the top 20 significantly enriched pathways (*q-value* < 0.05) were listed. **C** KEGG enrichment analysis of common target for Alo and inflammation; the top 20 significantly enriched pathways (*q-value* < 0.05) were listed. **D** Protein-protein interaction network analysis of common target for Alo and inflammation. The closer the color is to yellow, the higher the degree; the closer it is to green, the lower the degree. **E** Results of molecular docking for Alo and core targets

By searching databases, 516 targets related to Alo were screened, and 1086 targets related to inflammation were collected; as shown in Fig. 1A, a total of 68 overlapping targets were computed, the overlapping targets accounted for 4.4% of the total, indicating that the effects of Alo on inflammation were associated with multiple overlapping targets. All targets for Alo and inflammation are presented in Table S1.

### GO enrichment

By GO enrichment analysis of 68 overlapping targets intersecting Alo and inflammatory, 1561 GO items were obtained (*q-value* < 0.05), including 1334 biological process (BP), 52 cellular component (CC), and 175 molecular function (MF) terms (Fig. 1B). According to the *q-value*, the top 20 enriched BPs, including the response to an organic substance, regulation of programmed cell death, response to stimulus, regulation of cell death, regulation of apoptotic process, programmed cell death, etc. The top 100 GO terms are presented in Table S2.

### KEGG pathway enrichment

KEGG analysis of the overlapping targets intersecting Alo and inflammatory showed that the key target genes were enriched in 80 KEGG enrichment items (*q-value* < 0.05). The top 25 pathways mainly included the pathways in cancer, prostate cancer, EGFR tyrosine kinase inhibitor resistance, breast cancer, kaposi sarcoma-associated herpesvirus infection, prolactin signaling pathway, and other signaling pathways(Fig. 1C). The top 100 KEGG pathways are listed in Table S3.

### Network analysis

To identify the potential hub targets of Alo and inflammation, the overlapping targets were introduced into the STRING network platform for PPI network analysis. The Alo-inflammation target network was generated using Cytoscape 3.9.1. The three important parameters of degree (11.0794), betweenness (70.4762), and closeness (0.00784) were used to evaluate the hub targets. Based on the above screening, the top 10 targets were selected as the hub targets, namely ALB, AKT1, IL-6, EGFR, SEC, MAPK14, PTEN, IGF1, HSP90AA1, and ESR1 (Fig. 1D).

### Molecular docking

The molecular docking results showed that the binding energies of the compounds and the four target proteins were less than 0 kcal/mol and that there was a strong binding effect. The lower binding energy of the compounds to the targets indicated better binding. As shown in Table 2, the docking binding exergies of Alo with ALB, AKT1, IL-6, and EGFR were < 0, indicating that Alo had good binding affinity with ALB, AKT1, IL-6, and EGFR, giving a spontaneous and stable binding.

**Table 2:** Docking studies of aloperine and core targets (kcal/mol)

Targets	PBDID	Residue involved in H bonding	Docking score (kcal/mol)	Combination type	Center grid box size		
					X center	Y center	Z center
ALB	4OR0	ALA-209 A, ALA-212 A, LEU-326 A, LEU-346 A, LEU-330 A, LYS-350 A, VAL-481 A	-8.6	Hydrophobic interactive	47.467	53.4	44.5
AKT1	6HHG	TRP-80 A, THR-82 A, VAL-270 A, ASP-292 A, TYR-272 A	-8.0	Hydrogen bonds, Hydrophobic interactive, Salt bridges	30.678	29.283	33.467
IL-6		GLU-94 A, VAL-95 A, VAL-95 A, PRO-138 A, ASN-143 A	-6.1	Hydrophobic Interactions	41.328	42.289	25.95
EGFR	8A2B	LYS-745 A, MET-766 A, LEU-788 A, THR-790 A, GLY-857 A, ASP-855 A	-8.0	Hydrogen bonds, Hydrophobic interactive, Salt bridges	28.144	19.038	18.211

As shown in Fig. 1E; Table 2, Alo bound to ALB by hydrophobic interactions bonds with ALA-209 A (length: 3.68Å), ALA-212 A (length: 3.62 Å), LEU-326 A (length: 3.96 Å), LEU-346 A (length: 3.72 Å), LEU-330 A (length: 3.73 Å), LYS-350 A (length: 3.61 Å), VAL-481 A (length: 3.34 Å). Alo was predicted to dock into the binding pocket of AKT1 via hydrophobic interactions bonds TRP-80 A (length: 3.3 Å), THR-82 A (length: 3.84 Å), VAL-270 A (length: 3.56 Å), and VAL-270 A (length: 3.64 Å), Hydrogen Bonds TYR-272 A (length: 2.04 Å), Salt Bridges ASP-292 A (length: 5.38 Å). Alo was predicated to dock into the binding pocket of IL-6 via hydrophobic interactions bonds GLU-94 A (length: 3.66Å), VAL-95 A (length: 3.76Å), VAL-95 A (length: 3.8Å), PRO-138 A(length: 3.78Å), ASN-143 A (length: 3.55Å). There are also 4 Hydrophobic Interactions, LYS-745 A (length: 3.55Å), MET-766 A (length: 3.62Å), LEU-788 A (length: 3.36Å), THR-790 A (length: 3.32Å), 1 hydrogen bond GLY-857 A (length: 2.96Å), 1 Salt Bridges ASP-855 A (length: 5.07Å) were predicted between Alo and EGFR. The combination of Alo with ALB, AKT1, IL-6, and EGFR through hydrophobic interactions, and aalt bridges indicated high stability.

### Effect of Alo on the BIECs-21 viability and the model established for inflammatory

**Fig. 2:**
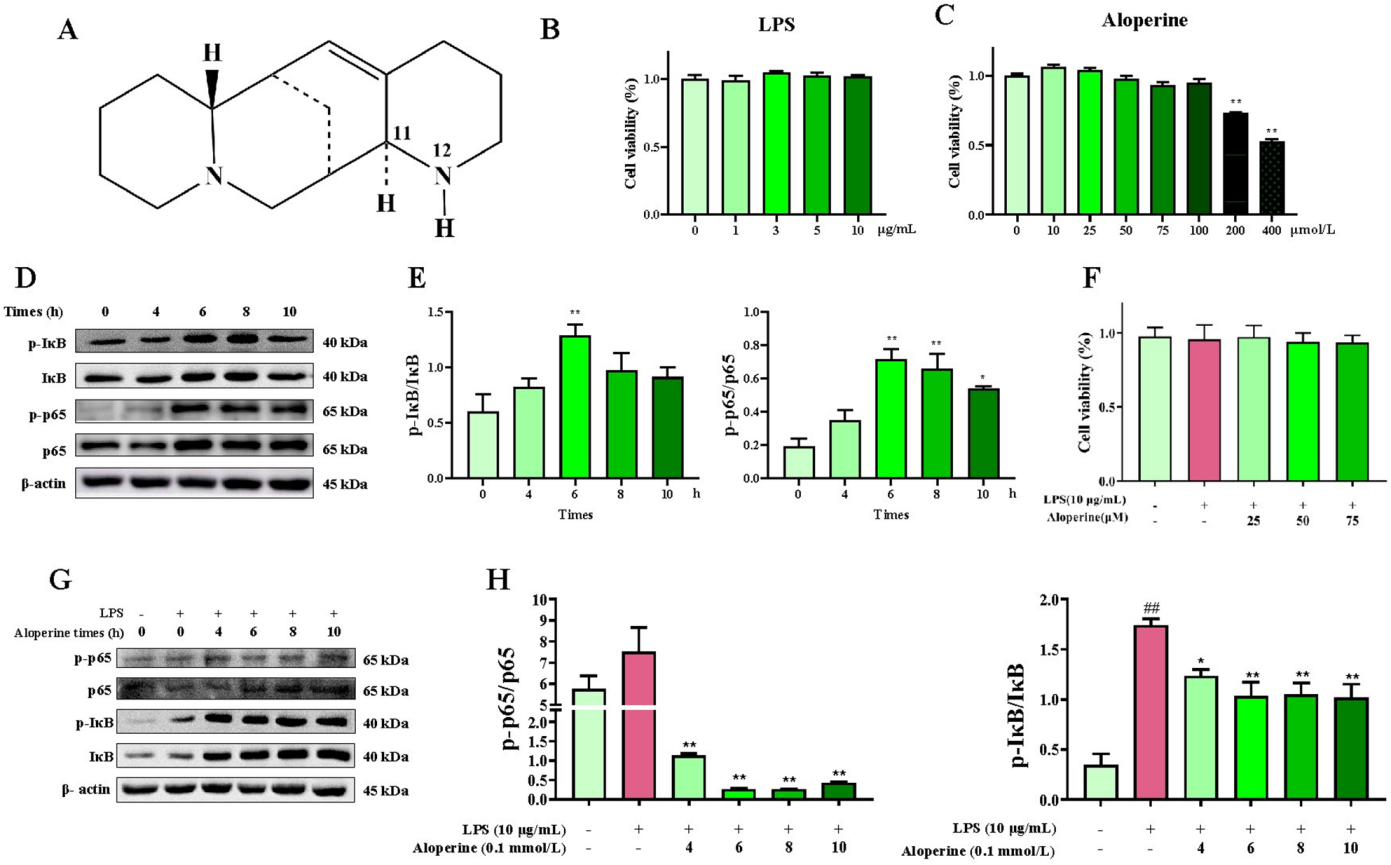
Screening of LPS and Alo concentration. **A** Structural formula of Alo. **B** Effect of LPS on the activity of BIECs-21. **C** Effect of Alo on the activity of BIECs-21, ** *P* < 0.01, as compared to the control group. **D-E** Effect of LPS in of inflammatory response key proteins expression; * *P* < 0.05, ** *P* < 0.01, as compared to the control group. **F** Effect of LPS and Alo on the activity of BIECs-21 cells. **G-H** Western blot analysis for the effect of Alo on LPS-induced inflammatory response key proteins expression, ## *P* < 0.01, as compared to the control group; * *P* < 0.05, ** *P* < 0.01, as compared to the LPS group

As shown in Fig. 2B, After treatment with LPS, the cell viability was not significantly changed at the range of 0–10 µg/mL doses of LPS; The cell viability was not significantly changed at the range of 0-100 µmol/L doses of Alo, while decreased at 200 and 400 µmol/L doses of Alo (*P* < 0.01) (Fig. 2C).

The 10 µg/mL dose of LPS was used to treat BIECs-21 at different times, and the radio levels of p-p65/p65 and p-IκB/IκB were the highest at the times of 6 h (Fig. 2D, E); Thus, the 10 µg/mL dose of LPS and treat with the times of 6 h was used to establish the inflammatory model. Based on the inflammatory model, BIECs-21 were treated with 100 µmol/L doses of Alo at different times, the radio levels of p-p65/p65 and p-IκB/IκB were lowest at 6 h after Alo treatment in Fig. 2G, H, and there was not significantly change cell viability after co-treatment of 25, 50 and 100 µmol/L doses of Alo with LPS (Fig. 2F); thus, the BIECs-21 were treated with Alo in 25, 50 and 100 µmol/L for 6 h to subsequent experiments.

### Effect of Aloperine on the LPS-induced inflammation response in BIECs-21

**Fig. 3:**
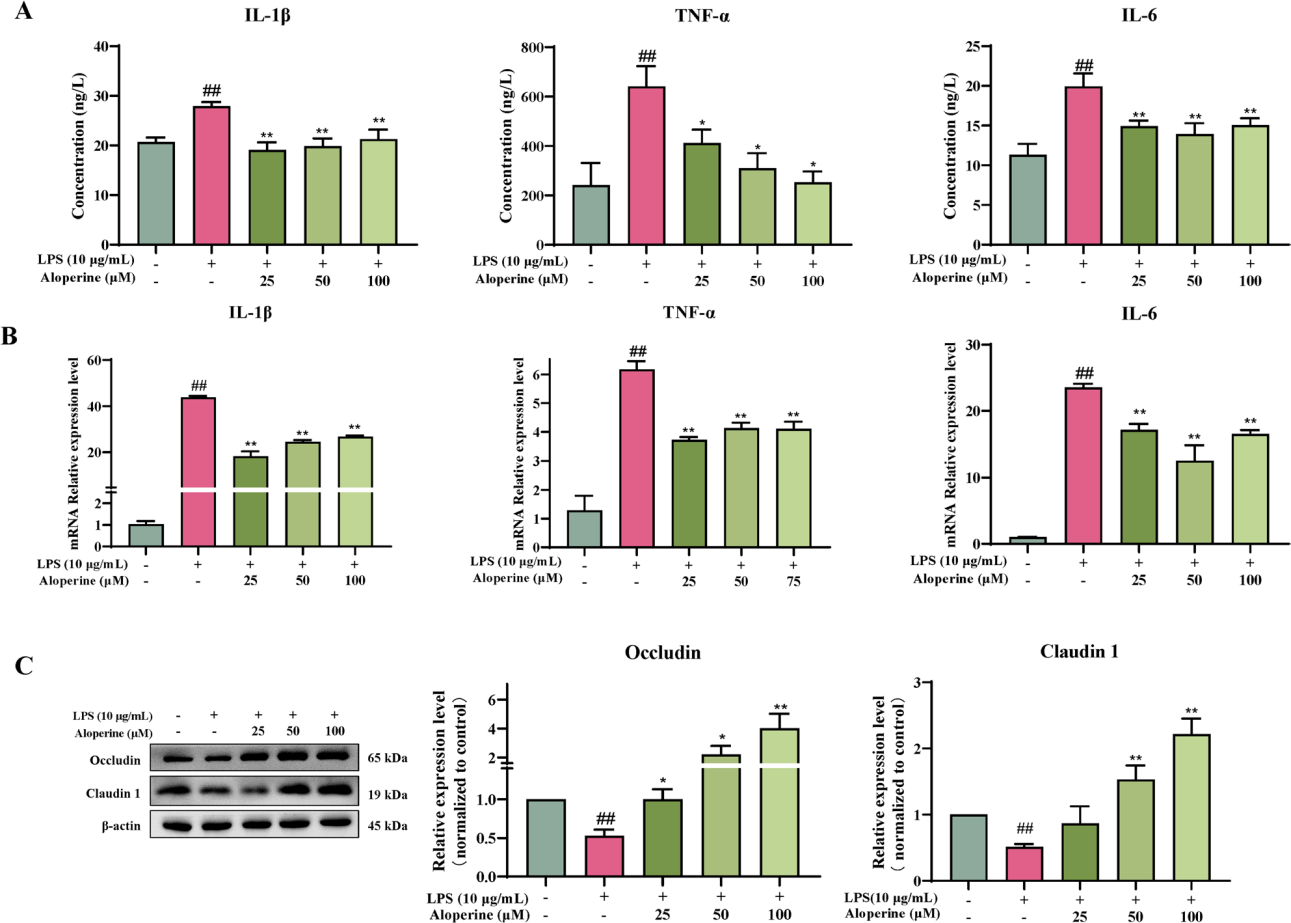
Effect of Alo on LPS-induced cytokine levels and intestinal barrier function-related proteins in BIECs-21 cells. **A** ELISA analysis for IL-1β, TNF-α and IL-6 concentration in cell supernatants. **B** RT-PCR analysis for IL-1β, TNF-α and IL-6 mRNA expression levels. Data are relative to control cells and normalized to housekeeping genes (GAPDH and 18 S). **C** Western blot analysis for the effect of Alo on intestinal barrier function-related proteins (Occludin and Claudin 1) in BIECs-21, Protein levels were normalized against the β-actin level. Data were shown as mean ± SEM and replicated three times. # *P* < 0.05, ## *P* < 0.01, as compared to the control group; * *P* < 0.05, ** *P* < 0.01, as compared to the LPS group

To investigate the anti-inflammatory activity of Alo, the concentrations of IL-1β, IL-6, and TNF-α were measured by ELISA in LPS-induced BIECs-21. As shown in Fig. 3A, LPS significantly increased the levels of IL-1β, IL-6, TNF-α, and IL-10 in the supernatant of BIECs-21 cells compared with the control group (*P* < 0.01); Alo treatment reduced the concentrations of IL-1β, IL-6, and TNF-α compared with the LPS group (*P* < 0.05 and *P* < 0.01). Similar to the results of RT-PCR results demonstrated that Alo treatment significantly decreased mRNA expression of IL-1β, IL-6, and TNF-α (Fig. 3B).

#### Effect of Aloperine on the LPS-induced tight junction in BIECs-21

To investigate the intestinal epithelial barrier function changes induced by LPS, ZO-1 and Claudin 1 protein expression levels were determined. As shown in Fig. 3C, LPS significantly down-regulated ZO-1 and Claudin 1 protein levels (*P* < 0.01). However, the proteins of ZO-1 and Claudin 1 with the treatment of Alo and LPS were significantly enhanced compared with the treatment of LPS Alone (*P* < 0.05 and *P* < 0.01). These results indicate that Alo promotes recovery from LPS-induced barrier dysfunction.

### Effect of Alo on the LPS-induced TLR4/p38 MAPK/NF-κB pathway

**Fig. 4:**
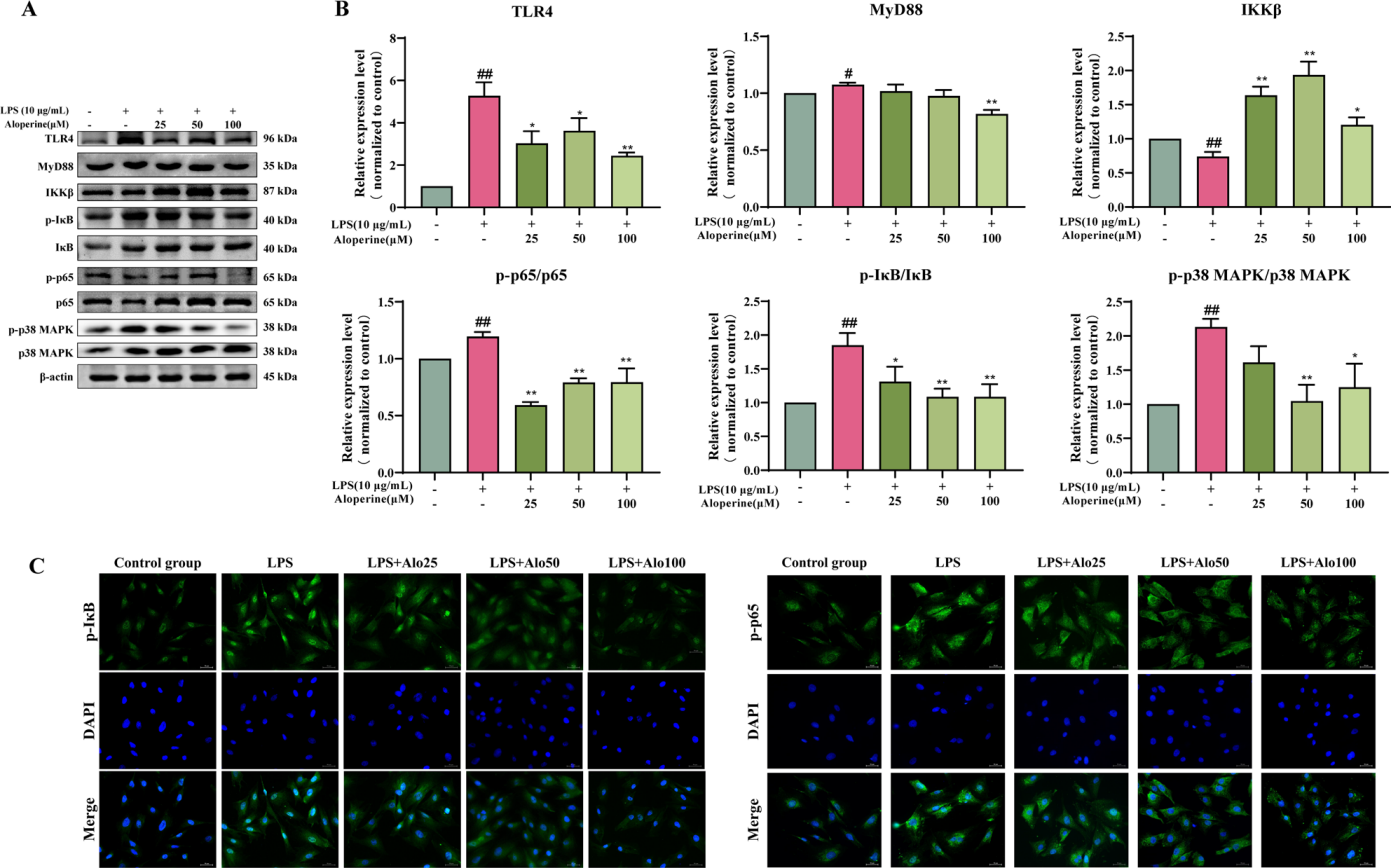
Effect of Alo on TLR4/p38 MAPK/NF-κB related proteins in BIECs-21. **A**-**B** Western blot analysis for the expression of TLR4/p38MAPK/NF-κB pathway related-proteins, protein levels were normalized against the β-actin level; # *P* < 0.05, ## *P* < 0.01, as compared to the control group; * *P* < 0.05, ** *P* < 0.01, as compared to the LPS group. **C** Immunofluorescence analysis for the effect of Alo on p-p65 and p-IκB expression in BIECs-21. Scale bars: 50 μm

NF-κB signaling plays a pathogenic role in many inflammatory diseases. As shown the Fig. 4A, B, LPS increased the expression of TLR4 and MyD88 (*P* < 0.05 and *P* < 0.01), and the ratio of p-p65/p65, p-IκB/IκB and p-p38/p38 levels (*P* < 0.01), decreased the expression of IKKβ (*P* < 0.01) compared with the control group, however, Alo inhibited the expression levels of TLR4, MyD88 (*P* < 0.05 and *P* < 0.01), the ratio of p-p65/p65, p-IκB/IκB, p-p38 MAPK/p38 MAPK levels (*P* < 0.05 and *P* < 0.01), and increased the IKKβ protein level (*P* < 0.05 and *P* < 0.01) compared with the LPS group. In addition, the fluorescence intensity of p-p65 and p-IκB was lowest among these groups compared with the LPS group after treatment with Alo (Fig. 4C).

#### Effect of Alo on the LPS-induced autophagy

**Fig. 5:**
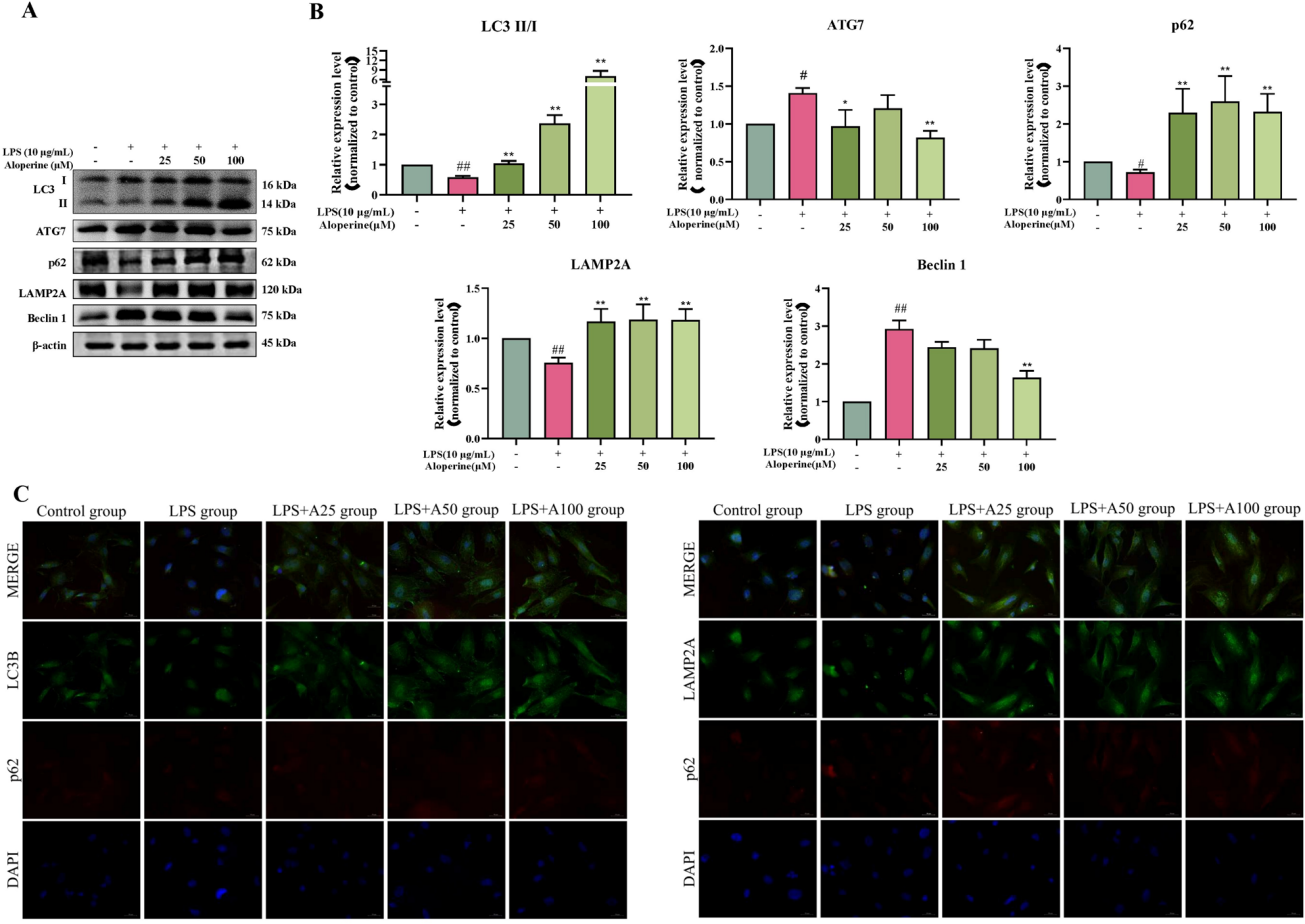
Effect of Alo on autophagy related-proteins. **A-B** Western blot analysis for the expression of related-proteins in autophagy, protein levels were normalized against the β-actin level; ## *P* < 0.01, as compared to the control group; ** *P* < 0.01, as compared to the LPS group. **C** Immunofluorescence analysis for the colocalization of p62 and LC3, p62 and LAMP2A. Scale bars: 50 μm

To clarify whether autophagy was involved in the underlying mechanism of Alo on LPS-induced BIECs-21, we assessed the effect of Alo on LPS-stimulated autophagy marker proteins LC3, p62, Beclin 1, and LAMP2A proteins expression in Fig. 5A, B. Compared with the control group, LPS upregulated the ATG7 and Beclin 1 protein expression (*P* < 0.05 and *P* < 0.01), downregulated the p62 and LAMP2A protein expression, and LC3 II/I ratio (*P* < 0.05 and *P* < 0.01). After LPS and Alo co-treatment, the LC3 II/I ratio, p62, and LAMP2A proteins expression were significantly increased compared with the LPS group (*P* < 0.01); ATG7 protein expression was significantly decreased in the 25 and 100 Alo groups (*P* < 0.05 and *P* < 0.01); Beclin 1 was significantly decreased 100 group (*P* < 0.01). The fluorescence intensity of p62 and LC3 in the LPS group was lowest among these groups (Fig. 5C).

**Fig. 6:**
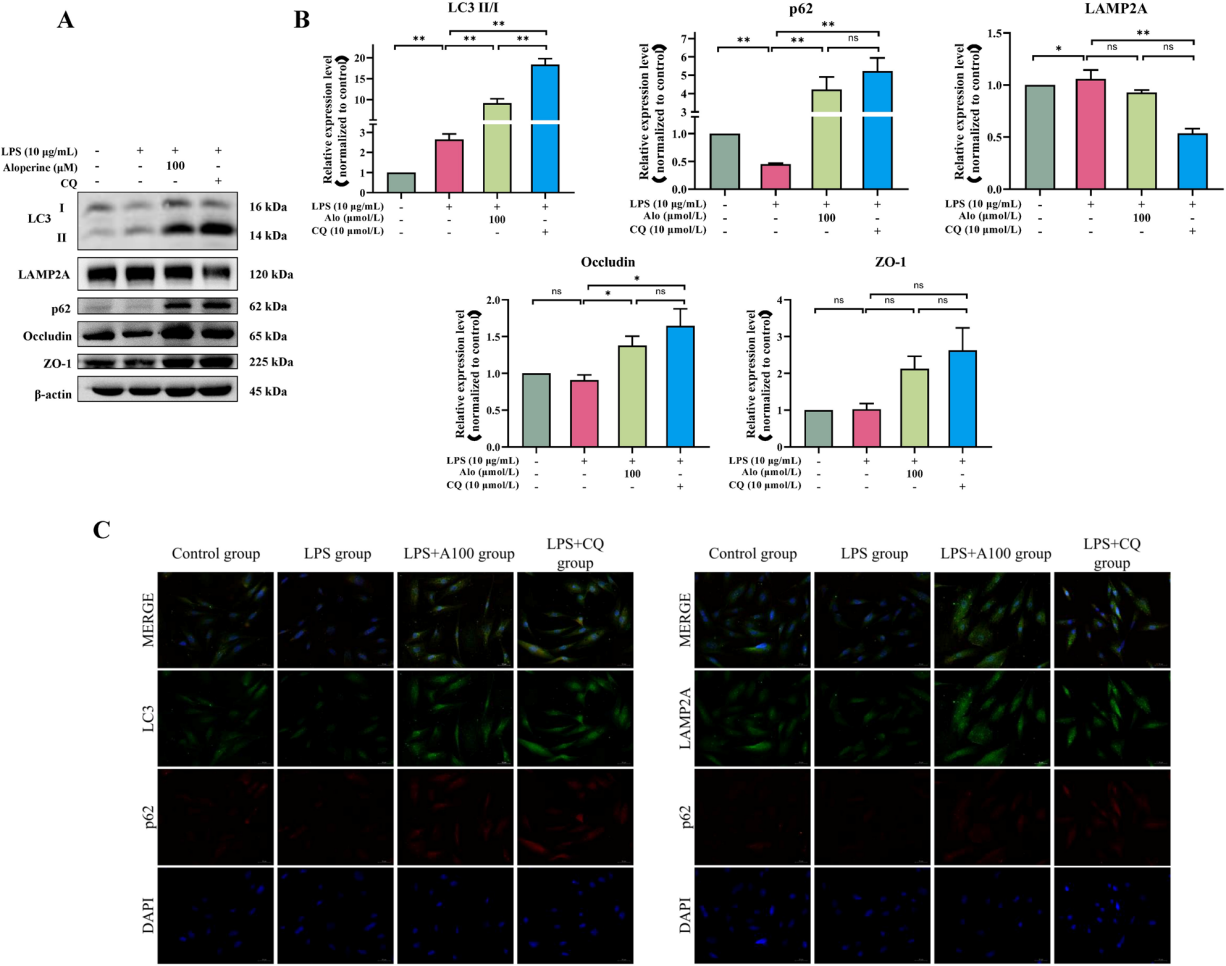
Effect of CQ on autophagy and intestinal barrier function related-proteins in BIECs-21. **A**-**B** Western blot analysis for the expression of related-proteins in autophagy and intestinal barrier function, protein levels were normalized against the β-actin level; * *P* < 0.05 indicated a significant difference, ** *P* < 0.01 indicated a highly significant difference, ns indicated not significant difference. **C** Immunofluorescence analysis for the colocalization of p62 and LC3, p62 and LAMP2A. Scale bars: 50 μm

To determine autophagy activity, cells were treated with the autophagy inhibitor chloroquine (CQ), which suppresses autophagy by inhibiting lysosome-autophagosome fusion but does not prevent the conversion of LC3 I to LC3 II, leading to the accumulation of LC3 II, and LC3 II and p62 are widely used to monitor intracellular autophagy flux [19]. After co-treatment with CQ and LPS (Fig. 6A, B), the LC3 II/I ratio, p62 and Occludin expression levels were remarkably increased compared with the LPS group (*P* < 0.05 and *P* < 0.01), the LAMP2A expression level was significantly decreased (*P* < 0.01), while the ZO-1 expression level trended to increase; The fluorescence intensity of p62 and LC3B in the LPS + CQ group was highest among these groups, and the LAMP2A was lowest among (Fig. 6C).

**Fig. 7:**
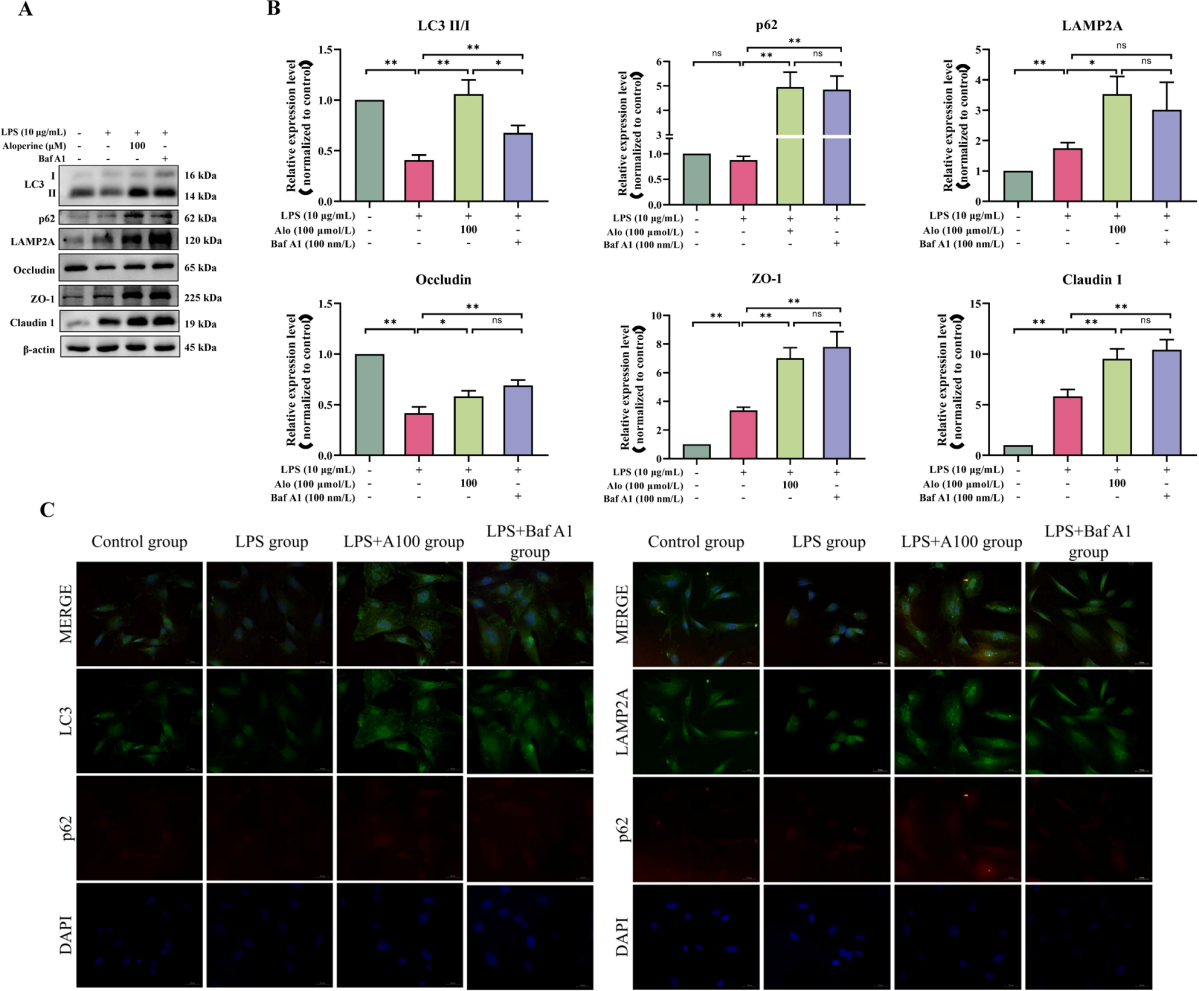
Effect of Baf A1 on autophagy and intestinal barrier function related-proteins in BIECs-21. **A-B **Western blot analysis for the expression of related-proteins in autophagy and intestinal barrier function, protein levels were normalized against the β-actin level; *P<0.05 indicated a significant difference, ** *P<*0.01 indicated a highly significant difference, ns indicated not significant difference. **C **Immunoflourescence analysis for the colocalization of p62 and LC3, p62 and LAMP2A. Scale bars: 50 μm

To further determine autophagy activity, cells were treated with autophagy inhibitor Bafilomycin A1 (Baf A1), an autophagy inhibitor at the late stage, which blocks autophagosome-lysosome fusion and inhibits acidification and protein degradation in lysosomes of cultured cells [20]. LC3 II and p62 are widely used to monitor intracellular autophagy flux. Compared with the LPS group (Fig. 7A, B), the combination treatment with Baf A1 and LPS remarkably enhanced the LC3 II/I ratio level, the p62, ZO-1, Occludin, and Claudin 1 protein expression (*P* < 0.01), and LAMP2A expression tended to be increased. The fluorescence intensity of p62 and LC3B, p62 and LAMP2A in the LPS + Baf A1 group was the highest among these groups, and these results of co-treatment with Baf A1 and LPS are consistent with the LPS + 100 Alo group, suggesting that Alo inhibits the late stages of autophagy maturation (Fig. 7C).

## Discussion

Diarrhea is a common disease in calfs and has long posed significant challenges to the cattle industry [4]. Alo exhibits anti-inflammatory pharmacological effects and has been shown to reduce DSS-induced intestinal inflammation in ulcerative colitis [17]. However, whether Alo can alleviate intestinal inflammation caused by calf diarrhea remains unclear. Therefore, this study employed Network pharmacology to systematically investigate the underlying mechanisms of Alo in inflammation. The analysis identified 68 key targets of Alo in the treatment of inflammation by searching database information and analyzing network topology. GO and KEGG enrichment analyses were conducted on these key targets to explore the potential mechanisms through which Alo mitigates inflammation. Additionally, 13 hub targets were screened by PPI analysis, including ALB, AKT1, IL-6, EGFR, etc.; Molecular docking techniques were then used to predict the binding of drugs and target active sites through geometric and energy matching. The molecular docking results indicated that Alo exhibited good docking activity with ALB and AKT1. 

In calfs, small intestinal epithelial cells are the primary sites for nutrient absorption and transport, and intact intestinal epithelial cells play a critical role in maintaining the intestinal physical barrier. Changes in intestinal epithelial barrier permeability can trigger mucosal inflammation, potentially leading to intestinal diseases [21, 22]. Studies have shown that harmful substances, such as LPS, stimulate the release of various inflammatory cytokines (such as IL-1β, IL-6, and TNF-α) in the intestinal tract [21], and this was also confirmed in the present study. The intestinal mechanical barrier consists of epithelial cells whose function depends on tight junction (TJ) proteins and other molecular complexes [23]. TJs, which include Claudins, Occludin, and zonula occludin-1 (ZO-1) protein, seal the spaces between adjacent epithelial cells, serving as key structural components that reinforce barrier function [24, 25]. Elevated levels of pro-inflammatory cytokines can increase paracellular permeability primarily by disrupting TJ proteins such as Claudins and occludin [26]. Research indicates that pathogenic *E. coli* damages intestinal TJs in calfs by destabilizing the stability of epithelial TJ proteins, reducing the expression levels of Claudin 1, Occludin, and ZO-1, which increases epithelial permeability and impairs barrier function [27–29]. Numerous studies have demonstrated that LPS enhances intestinal barrier permeability by modulating TJ protein expression, ultimately causing intestinal barrier dysfunction [30, 31]. In the present study, LPS treatment significantly decreased the expression levels of Claudin 1, Occludin, and ZO-1, whereas treatment with Alo elevated their expression. These findings suggest that Alo counteracts the LPS-induced reduction in TJ protein expression (Claudin 1, Occludin, and ZO-1), thereby contributing positively to the restoration of the intestinal barrier [21, 32].

Impaired intestinal barrier function accelerates the translocation of pathogens and other harmful substances into the bloodstream, thereby promoting the development of inflammatory bowel disease [33, 34]. This also leads to an increased release of pro-inflammatory cytokines, which activate intestinal epithelial cells and trigger as well as amplify intestinal inflammation [26]. Research has demonstrated that LPS binding to TLR4 leads to the activation of transcription factors, including NF-κB and mitogen-activated protein kinase p38 (p38 MAPK) [21, 35]. Activated p38 MAPK results in elevated expression of the pro-inflammatory cytokine IL-6 [36]. Additionally, Alo, an alkaloid, inhibits the NF-κB and MAPK signaling pathways and reduces the production of pro-inflammatory cytokines [9, 37]. Studies have shown that Alo regulates inflammatory responses in DSS-induced colitis by suppressing the PI3K/AKT/mTOR signaling pathway [17]. In the present study, treatment with Alo significantly reduced the nucleation of the p-p65 and p-IκB, lowered the protein levels of TLR4, MyD88, and IKKβ expression, and decreased the phosphorylation levels of p65, IκB, and p38 MAPK. It also inhibited LPS-induced expression of IL-1β, IL-6, and TNFα by modulating the TLR4/p38 MAPK/NF-κB pathway. These findings indicate that Alo possesses potent anti-inflammatory effects and can inhibit LPS-induced activation of the TLR4/p38 MAPK/NF-κB signaling pathway.

In intestinal diseases, autophagy helps reduce pro-inflammatory signaling by removing damaged organelles in the cell, degrading molecules that promote inflammation, and regulating the production and release of inflammatory cytokines [38–40]. Additionally, the autophagic process plays a role in the growth of intestinal epithelial cells, maintaining the mucosal barrier, and preserving intestinal homeostasis [41]. Numerous studies have shown that activating autophagy can enhance the intestinal epithelial barrier and increase levels of TJ proteins [42–45]. However, some research indicates that inhibiting autophagy may help maintain tight junction integrity and contribute to anti-inflammatory effects [40, 46]. In this study, increased of the LC3 II/I ratio, p62, and LAMP2A protein expression suggests that Alo treatment inhibits LPS-induced autophagy. Moreover, we employed Baf A1 and CQ in assays of autophagy-related proteins, which further confirmed that Alo treatment disrupts autophagic degradation. Notably, we found that the autophagy inhibitor Baf A1 increased ZO-1 expression, suggesting that Alo treatment repairs intestinal barrier function by inhibiting autophagy through blocking autophagosome formation and impairing autophagosome-lysosome fusion.

## Conclusion

**Fig. 8:**
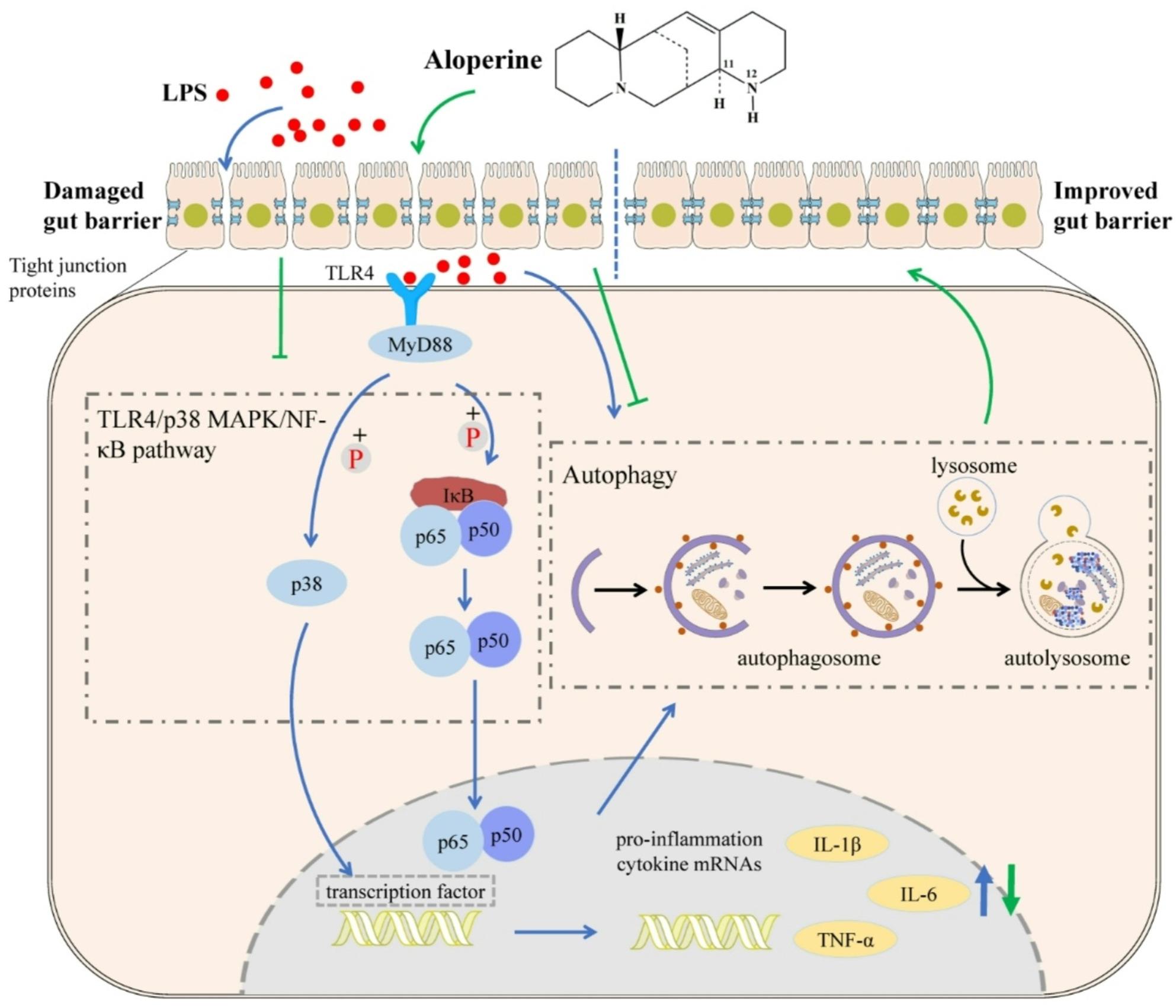
Mechanism of aloperine on LPS-induced inflammation through autophagy and TLKR4/p38 MAPK/NFκB pathway, Alo can reduce inflammatory cytokine levels and maintain the integrity of the intestinal barrier through the TLR4/p38 MAPK/NF-κB signaling pathway and autophagy

In this study, a comprehensive analysis integrating target prediction, signaling pathway examination, and molecular docking was conducted to identify the potential active ingredients and elucidate the molecular mechanisms underlying the anti-inflammatory activity of Alo. In vitro results demonstrate that Alo can reduce inflammatory cytokine levels and maintain the integrity of the intestinal barrier through the TLR4/p38 MAPK/NF-κB signaling pathway and autophagy (Fig. 8), indicating its potential value in treating intestinal diseases and effectively alleviating diarrhea.

## Supplementary Information

Supplementary Material 1.

Supplementary Material 2.

## Data Availability

This article contains the data presented in the present research and its supplementary Information.

## Abbreviations

Alo: Aloperine
BIECs-21: Bovine intestinal epithelial cells
LPS: Lipopolysaccharides
ALB: Albumin
AKT1: Serine/threonine kinase 1
IL-6: Interleukin-6
EGFR: Epidermal growth factor receptor
TLR4: Toll like receptors 4
p38 MAPK: p38 mitogen-activated protein kinase
p-p65: phospho-nuclear factor kappa-B
p65: nuclear factor kappa-B
IKKβ: Inhibitor of kappa B kinase
p-IκB: phospho-Inhibitor of NF-κB
IκB: Inhibitor of NF-κB
ZO-1: Zonula occludin-1
LAMP2A: Lysosome-associated membrane glycoprotein 2
LC3: Microtubule-associated protein 1 light chain 3
p62: Sequestosome 1
CQ: Chloroquine
Baf A1: Bafilomycin A1
