# Supplementary material for: Aloperine alleviates LPS-induced inflammation in bovine intestinal epithelial cells through autophagy and TLR4/p38 MAPK/NF-κB pathway
Source: BMC Vet Res. 2026 Feb 20;22:193. doi: 10.1186/s12917-026-05337-7 (PMC13032521; doi:10.1186/s12917-026-05337-7)

Figure 2 D

p-IκB

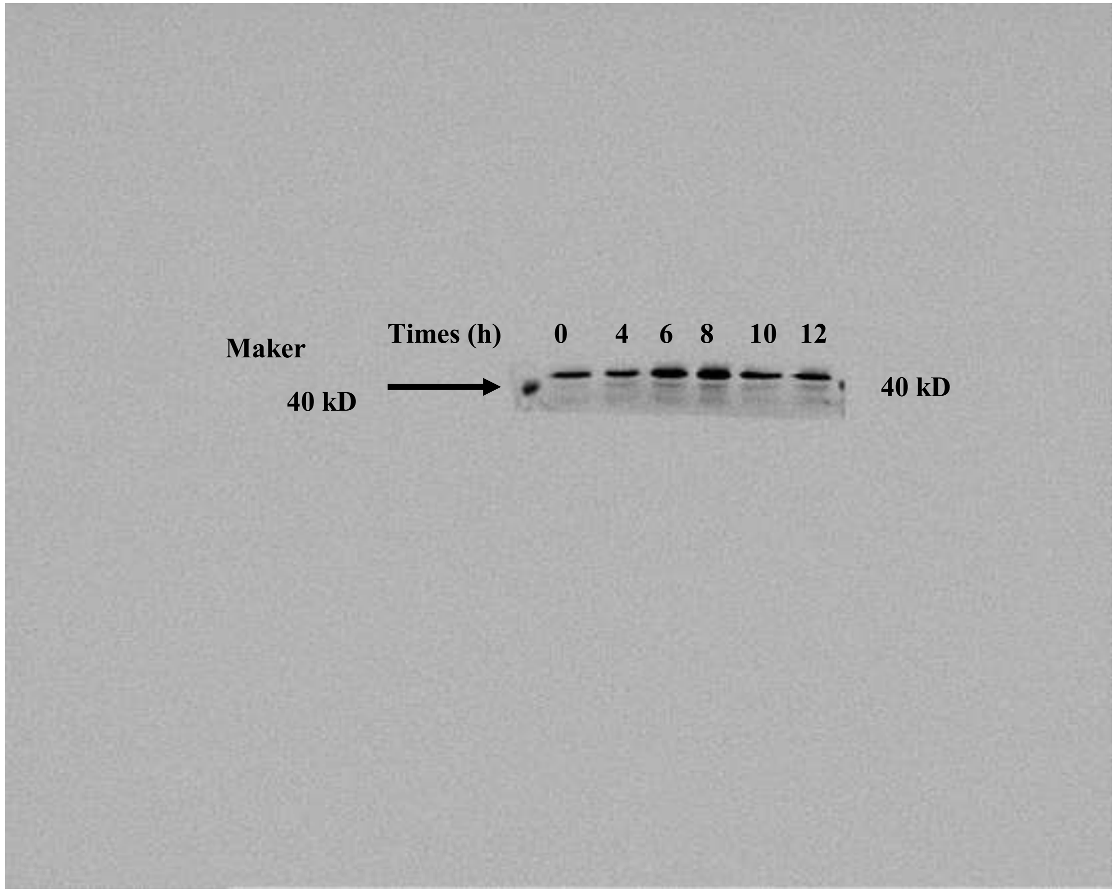

IκB

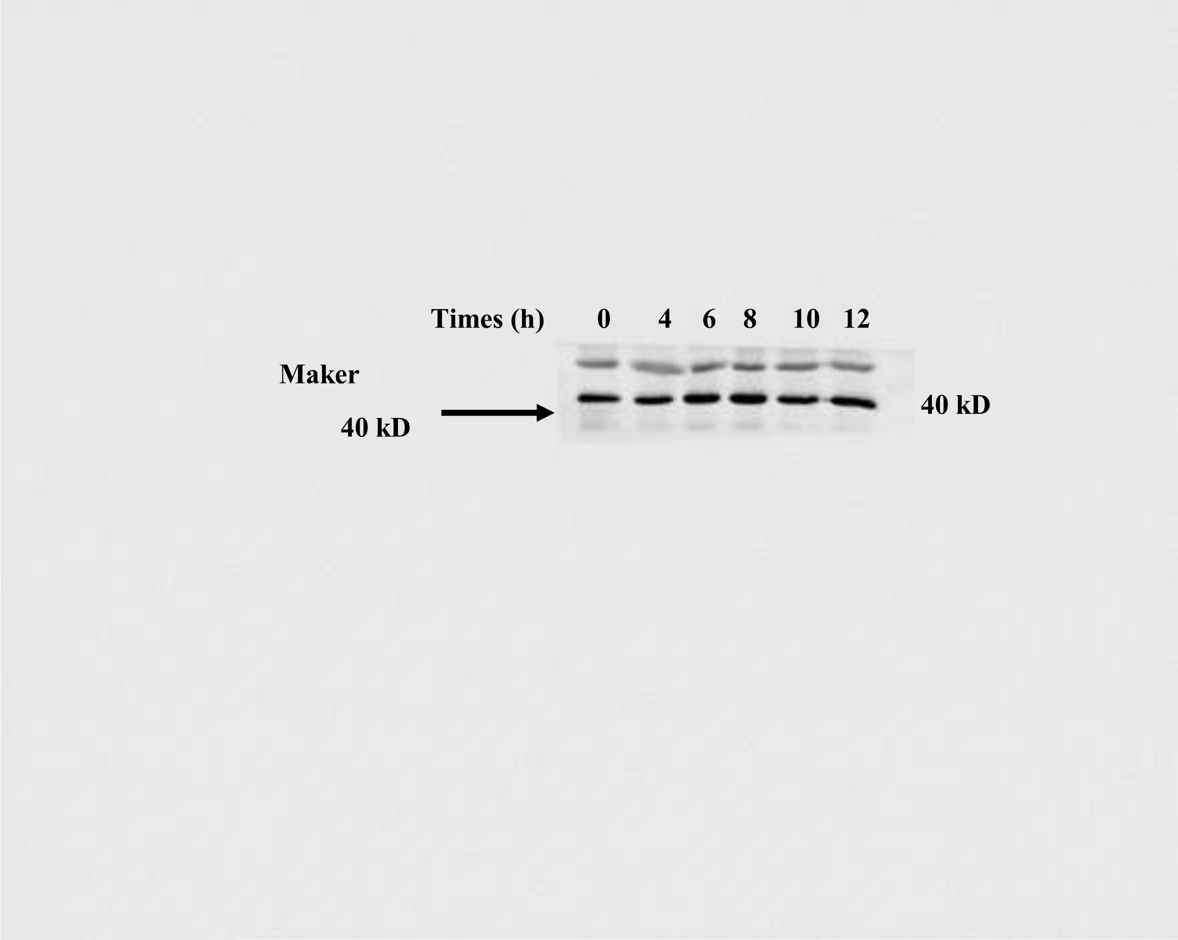

Figure 2 D

p-p65

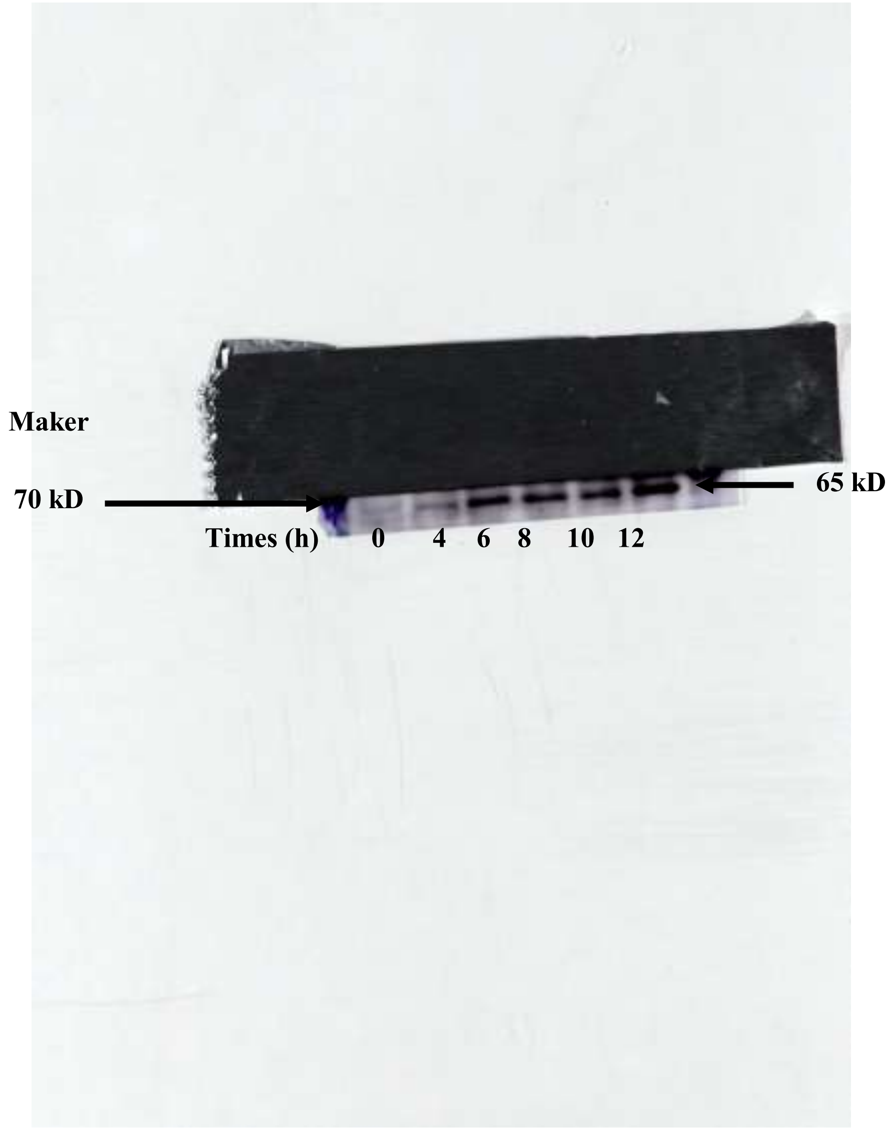

p65

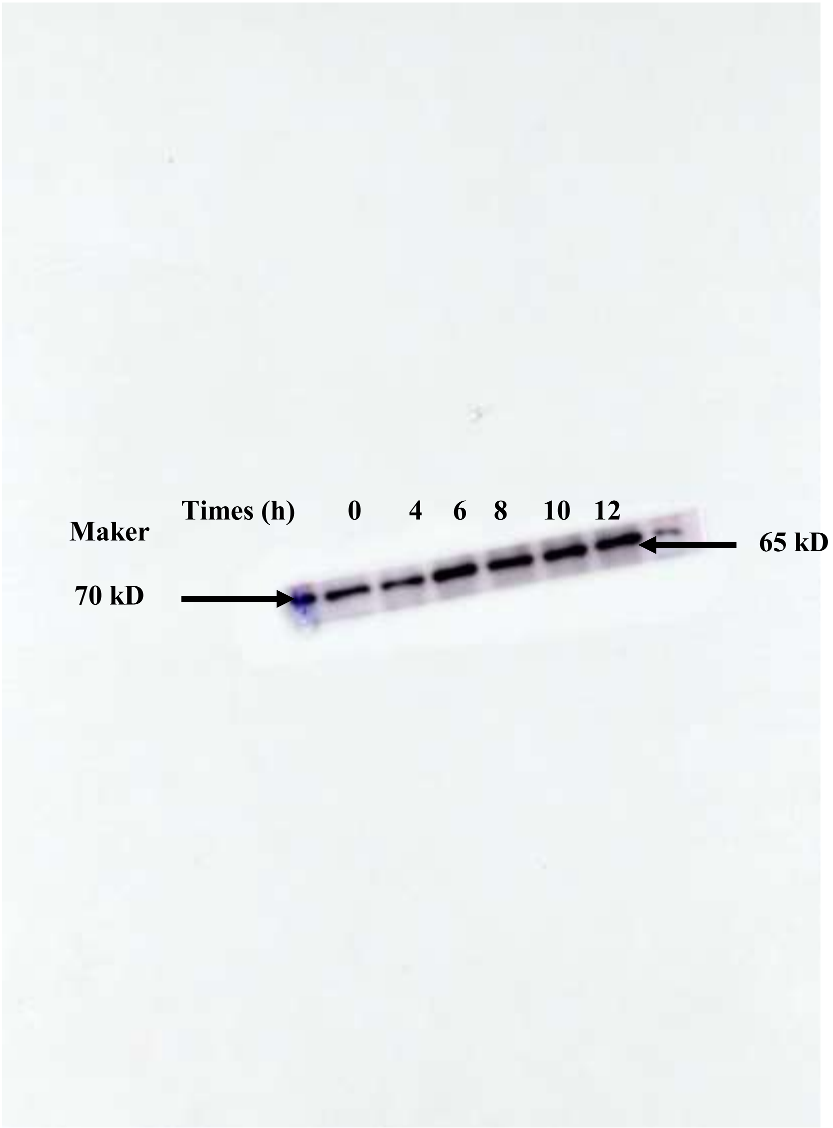

Figure 2 D

β-actin

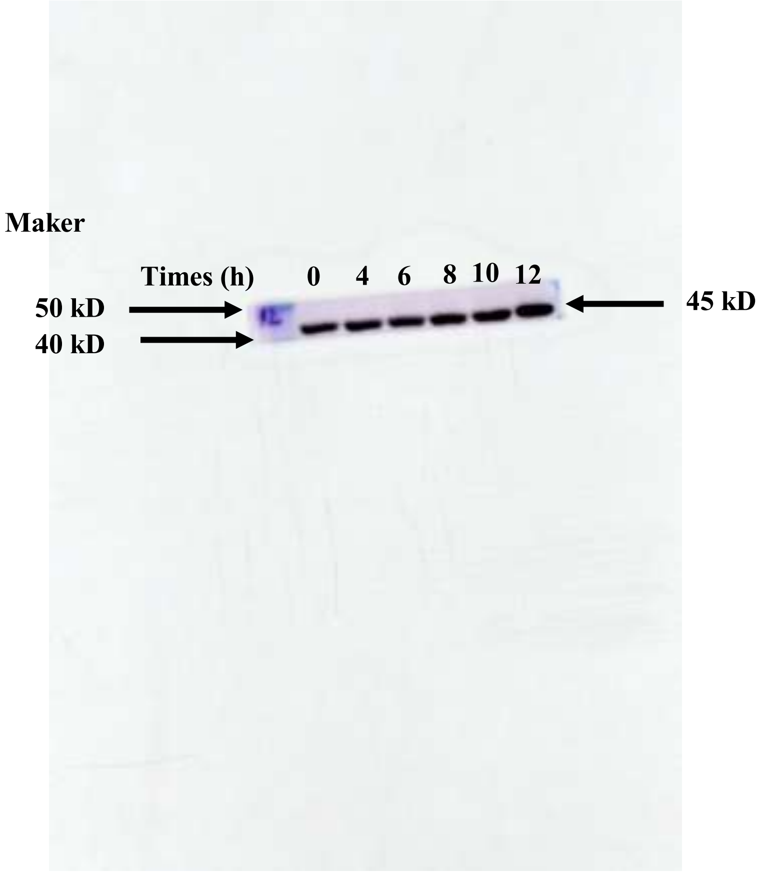

Figure 2 G

p-IκB

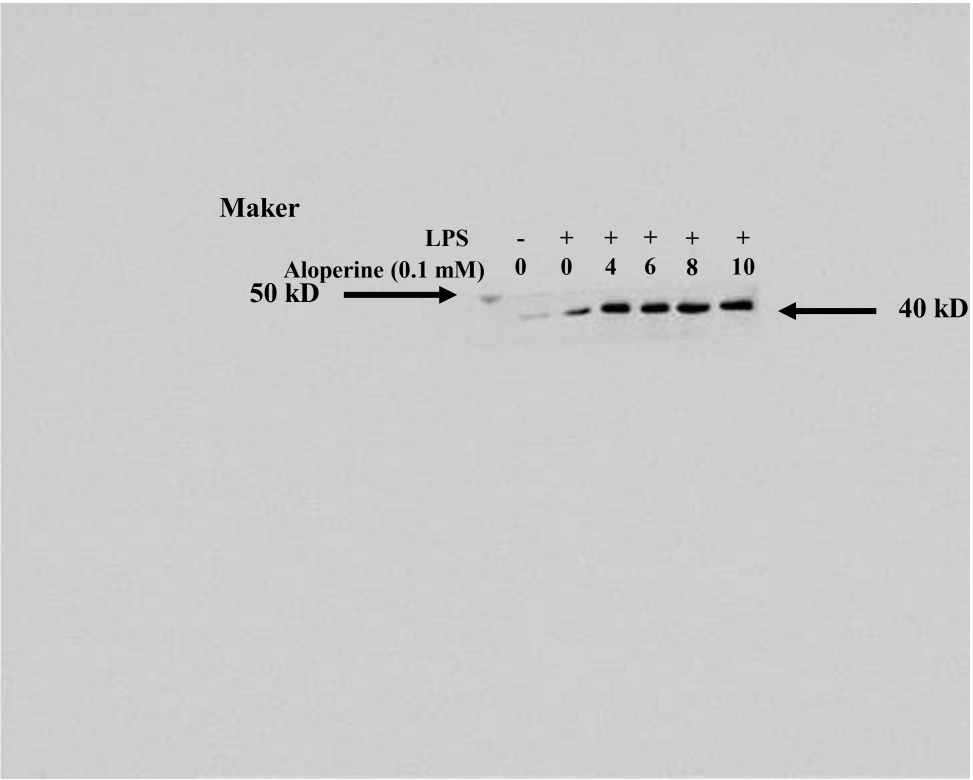

IκB

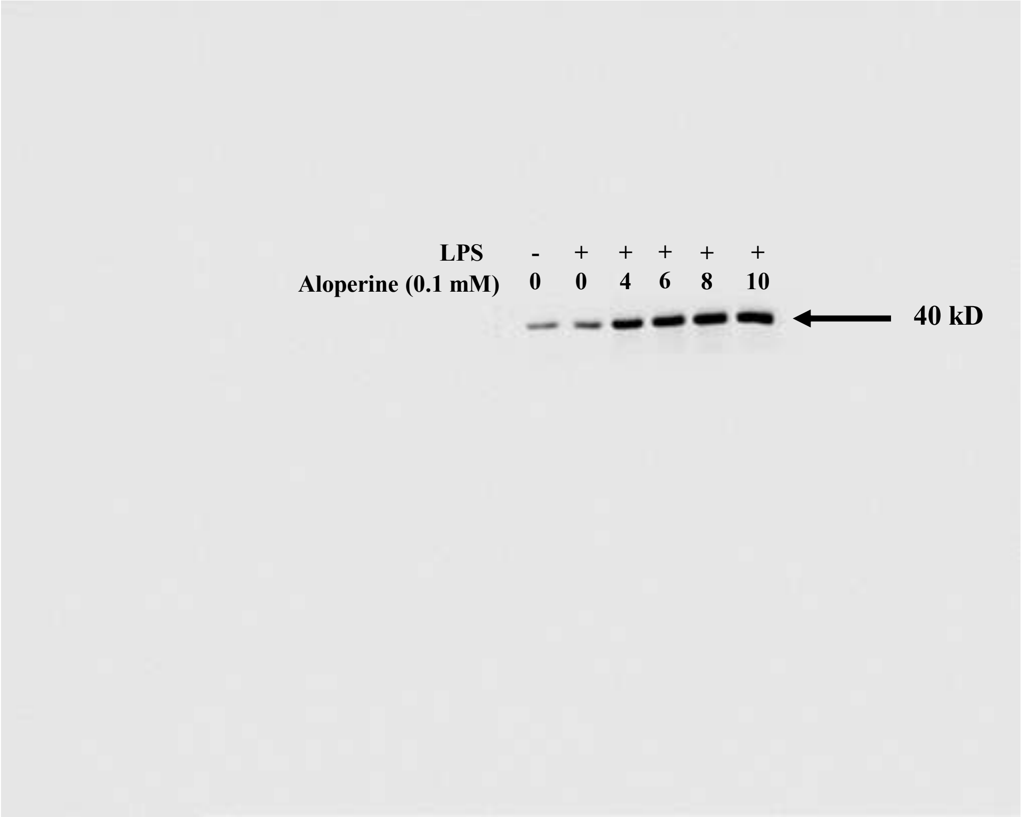

Figure 2 G

p-p65

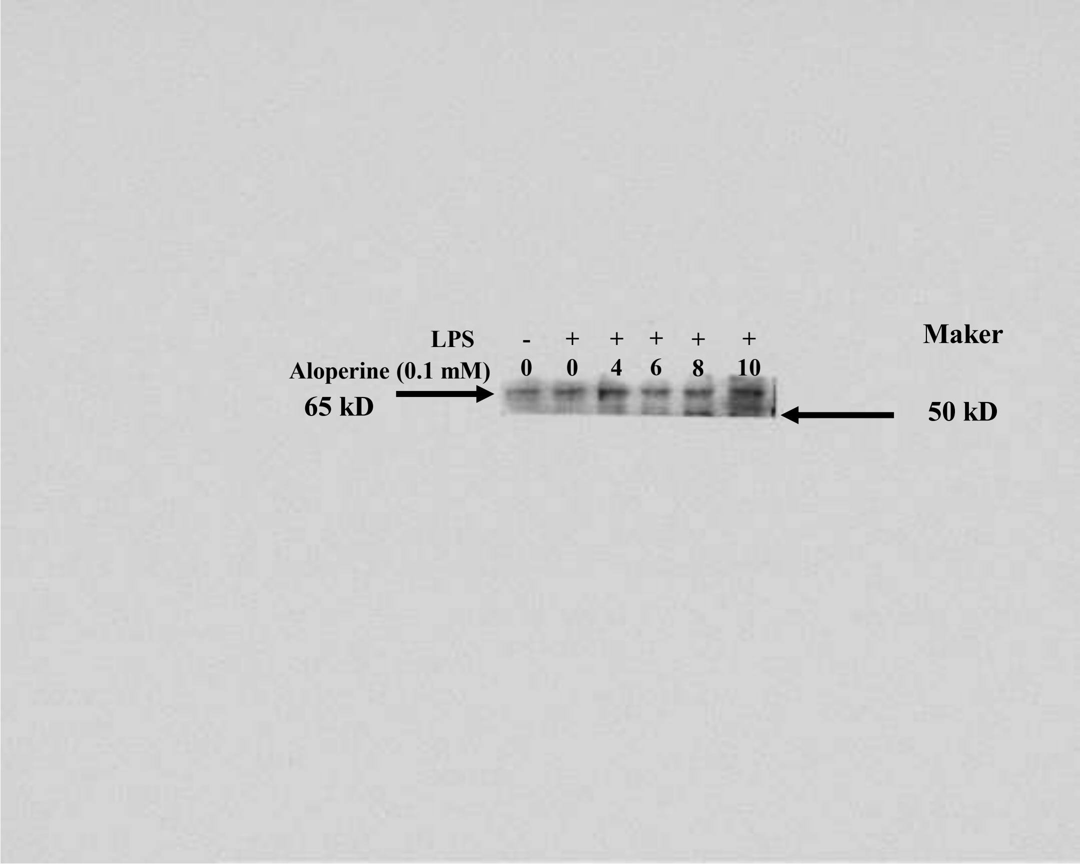

p65

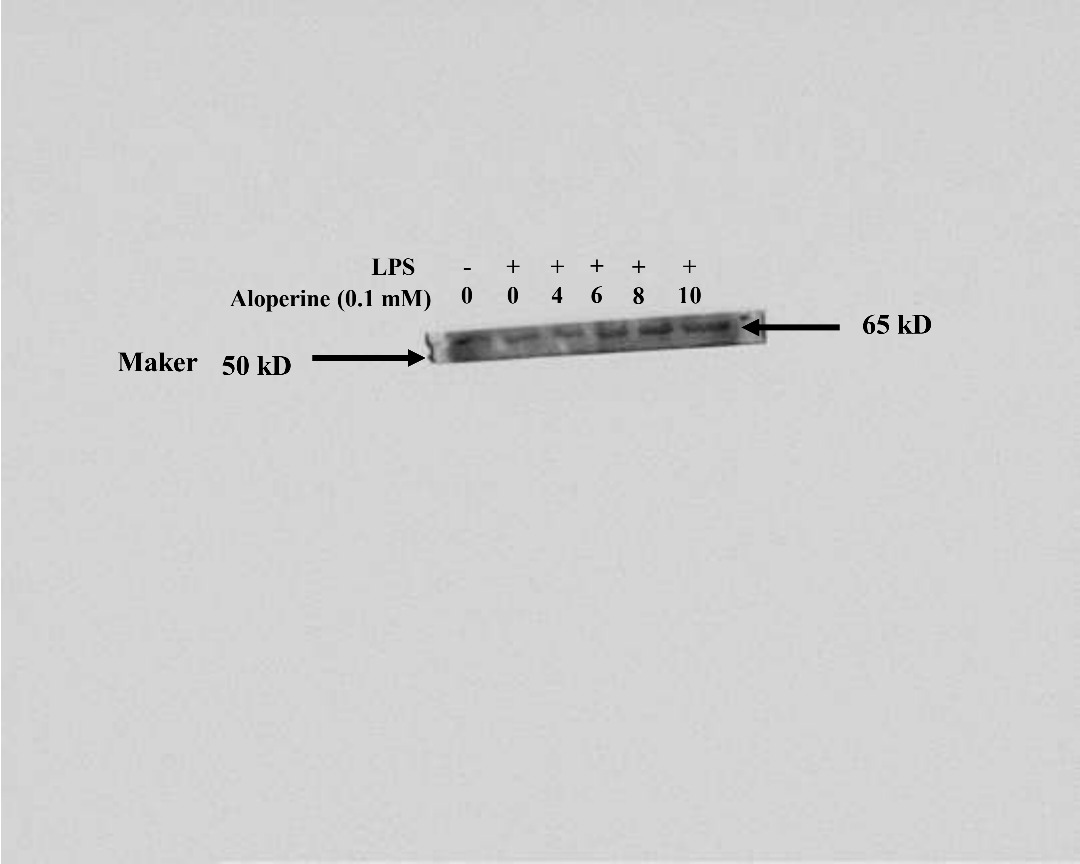

Figure 2 G

β-actin

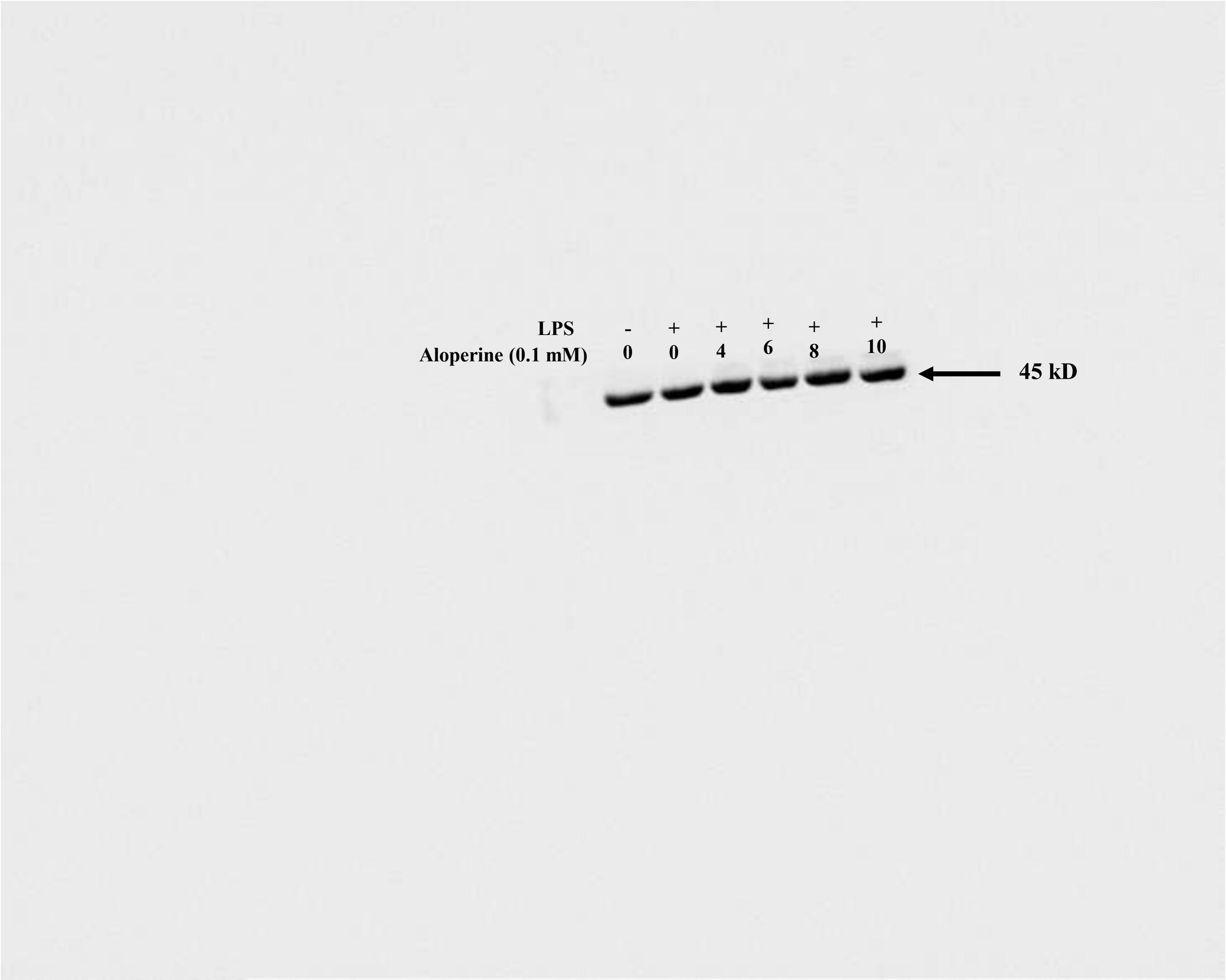

Figure 3

Claudin 1

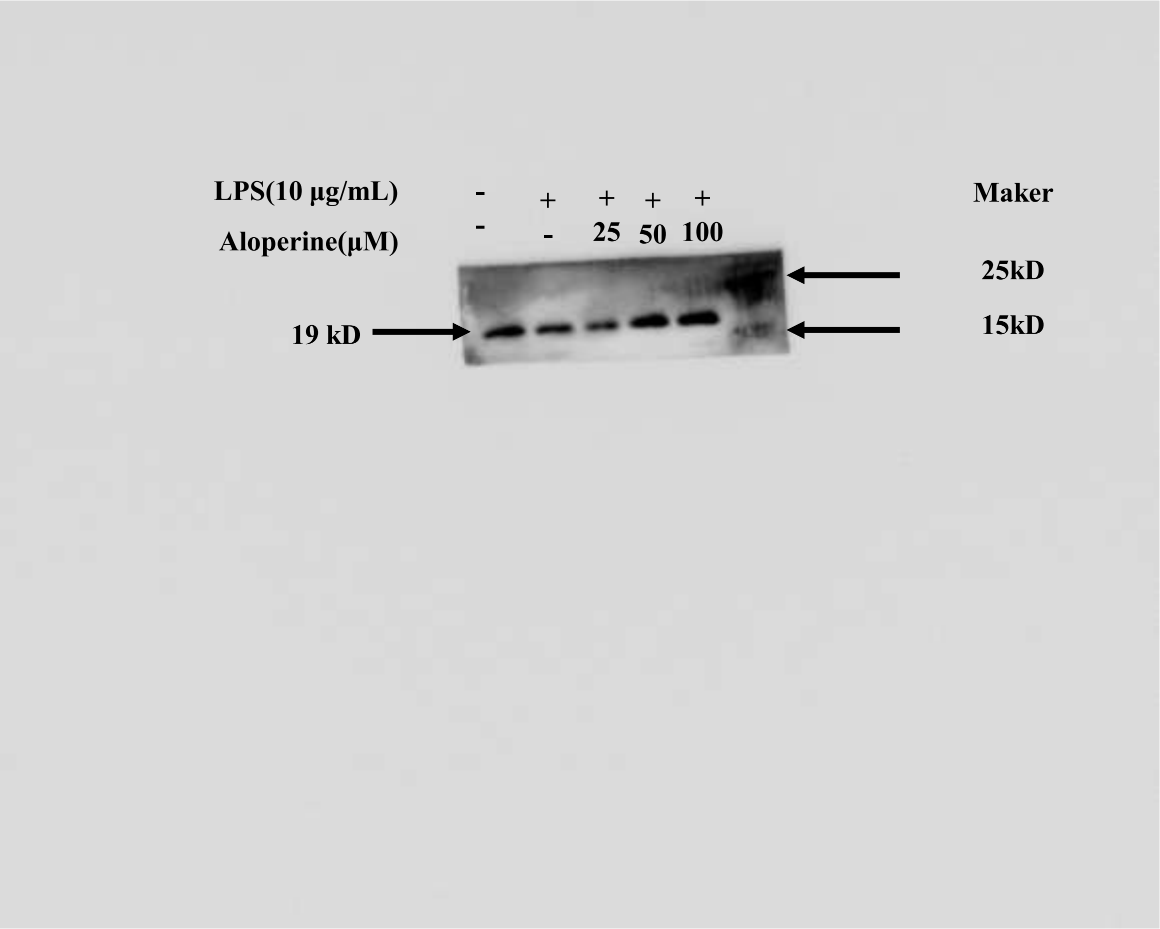

β-actin

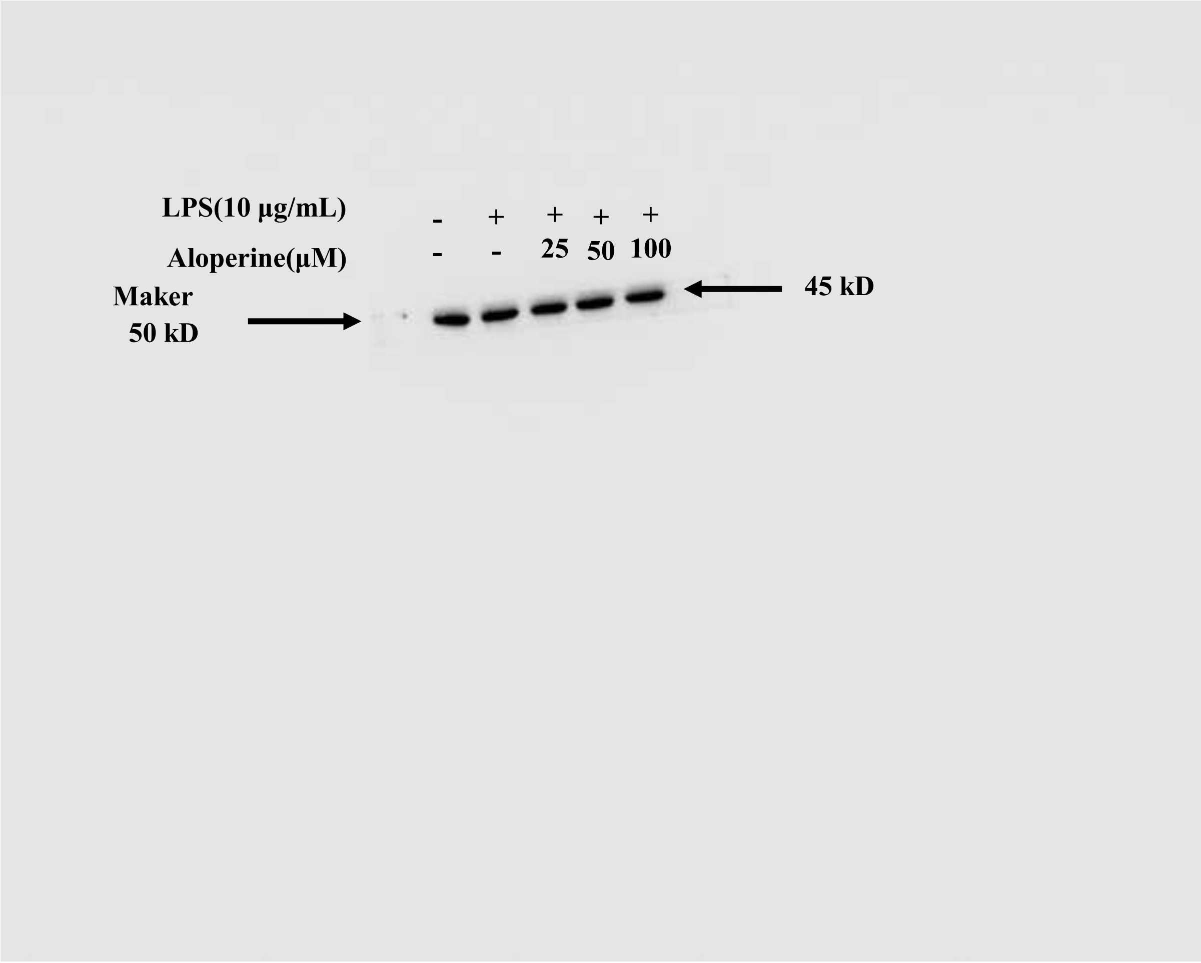

Figure 3

Occludin

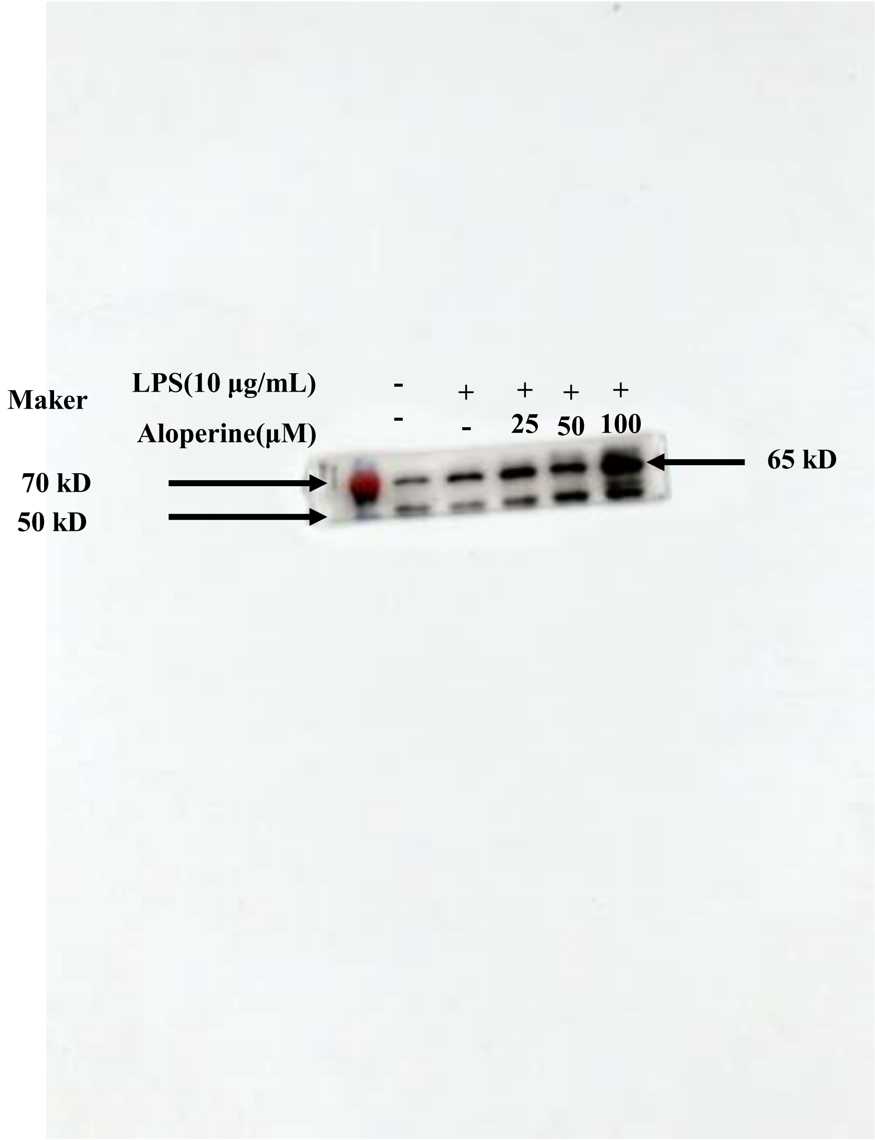

β-actin

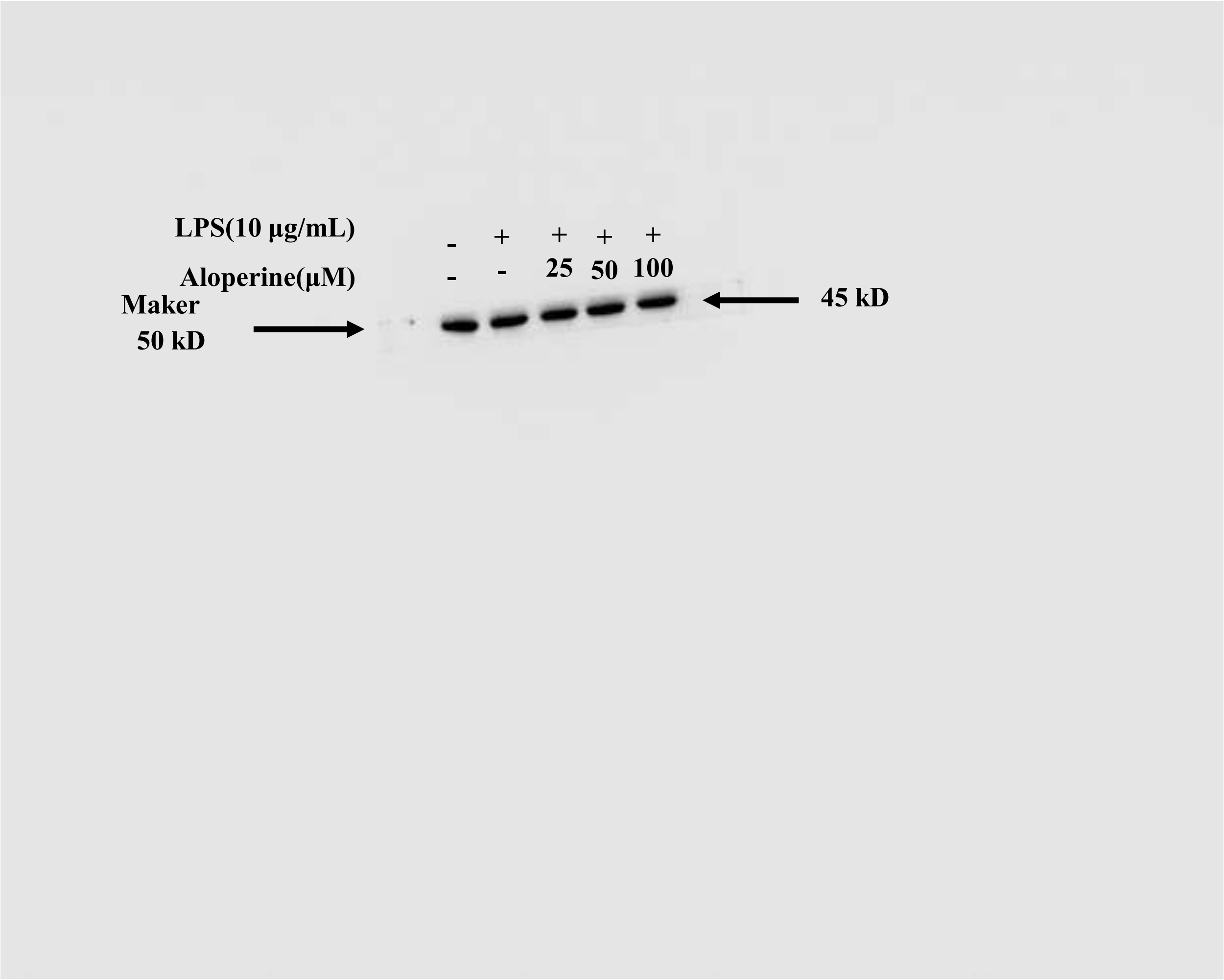

Figure 4

p-p38 MAPK

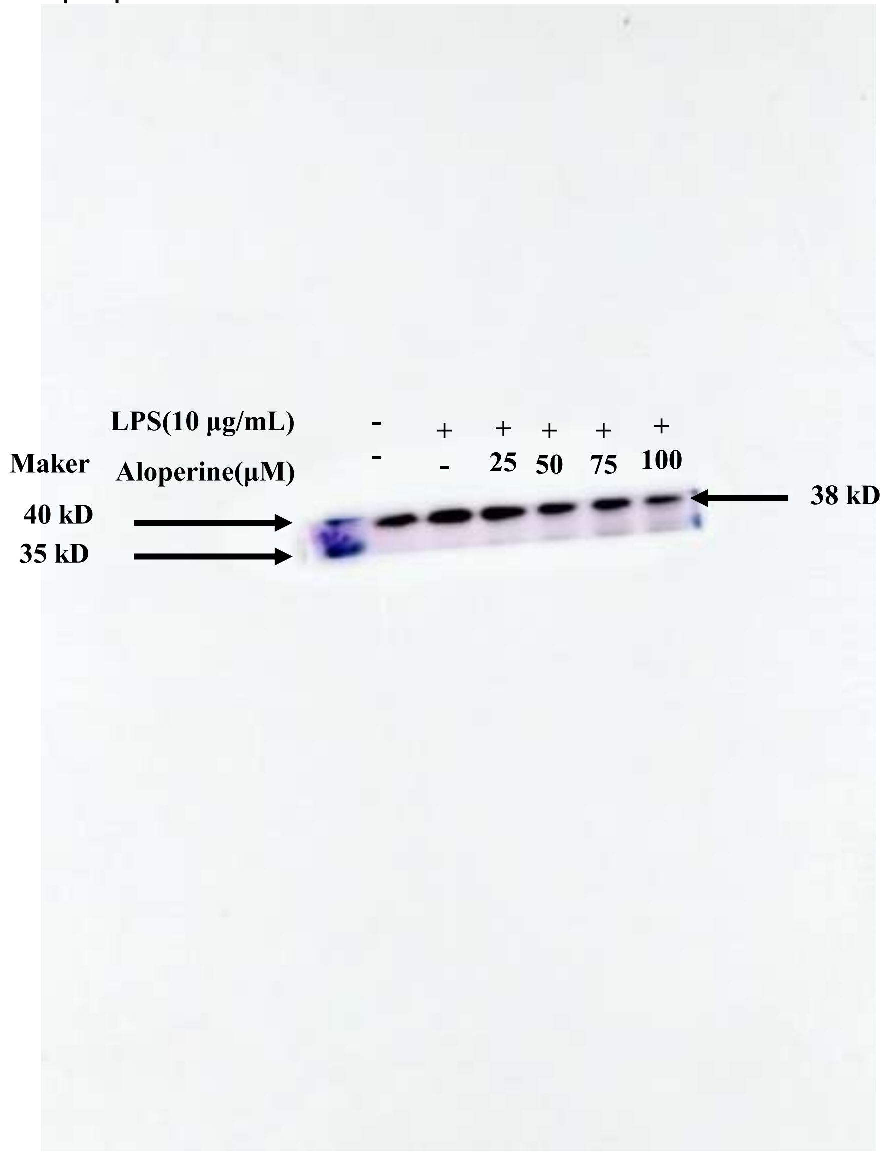

p38 MAPK

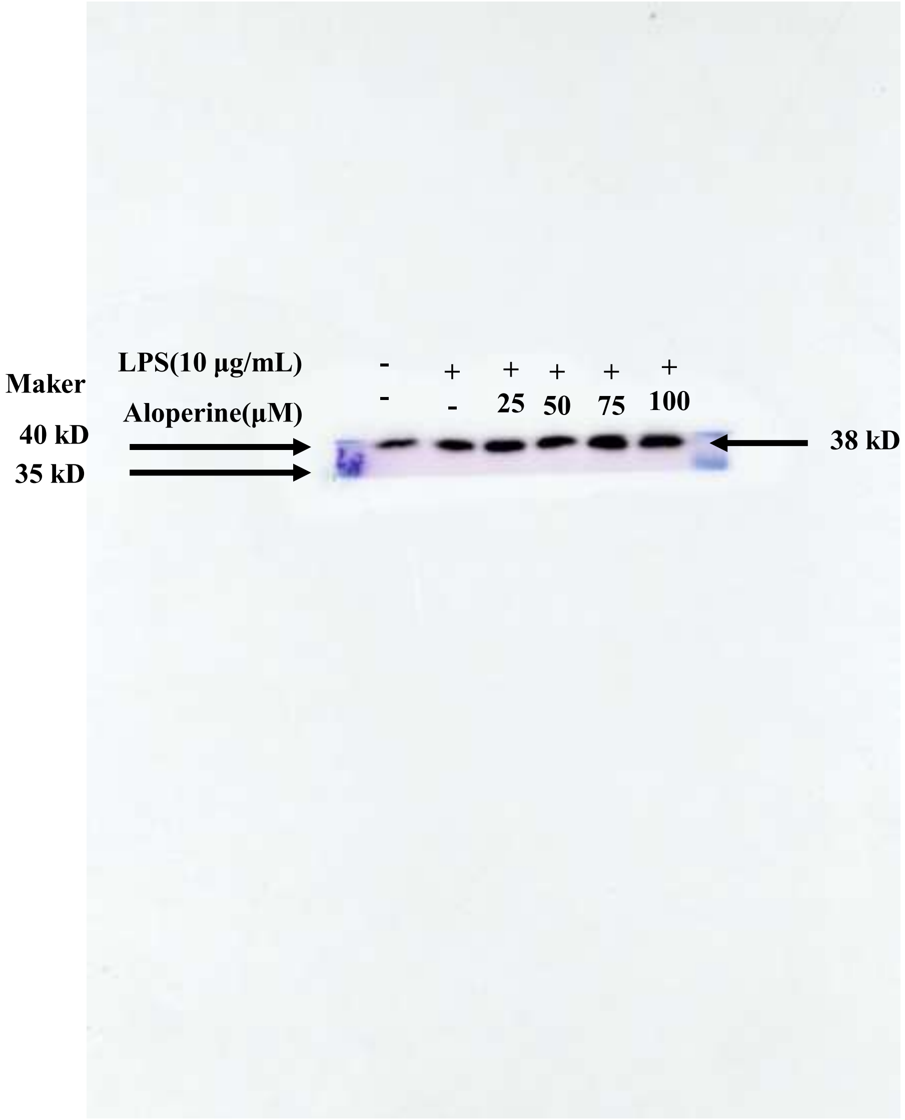

Figure 4

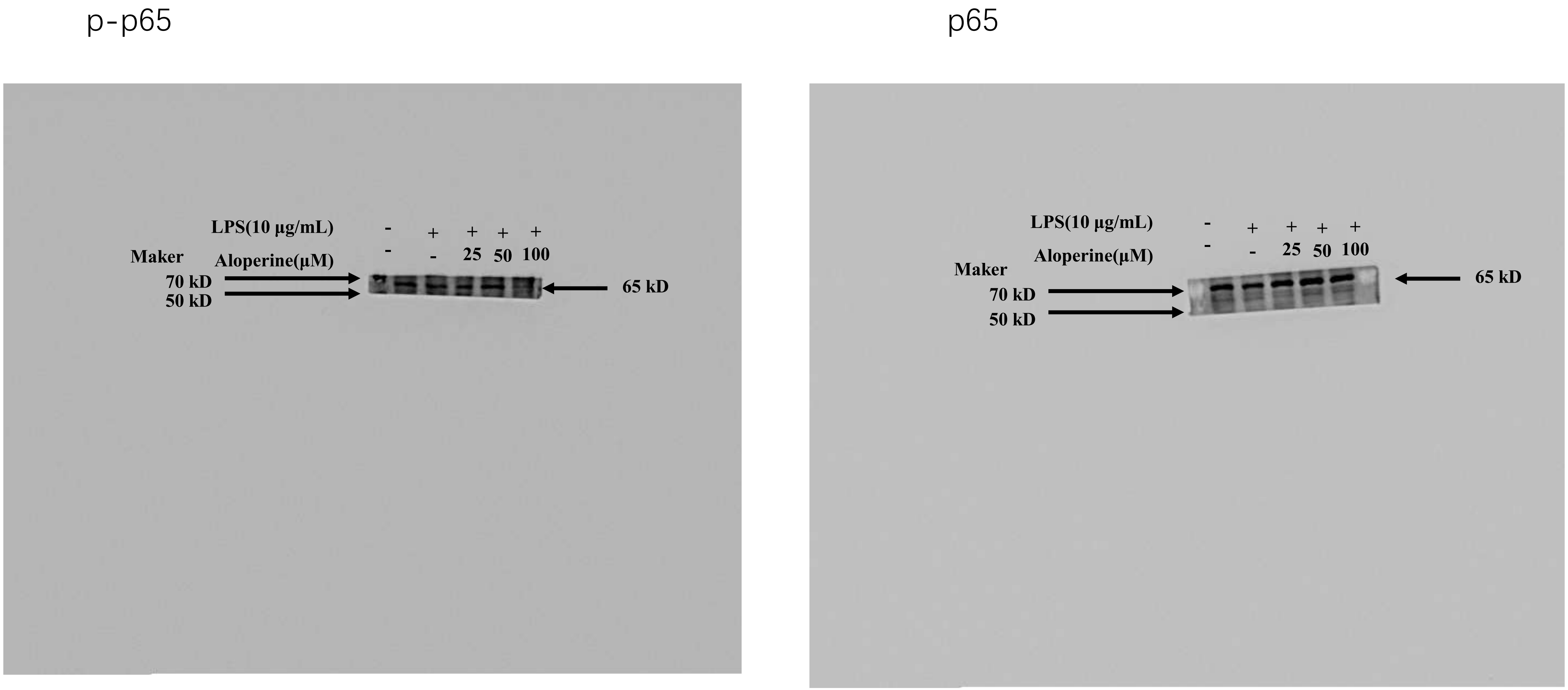
p-p65
LPS(10 μg/mL)
Aloperine(μM)
Maker
70 kD
50 kD
65 kD
p65
LPS(10 μg/mL)
Aloperine(μM)
Maker
70 kD
50 kD
65 kD

Figure 4

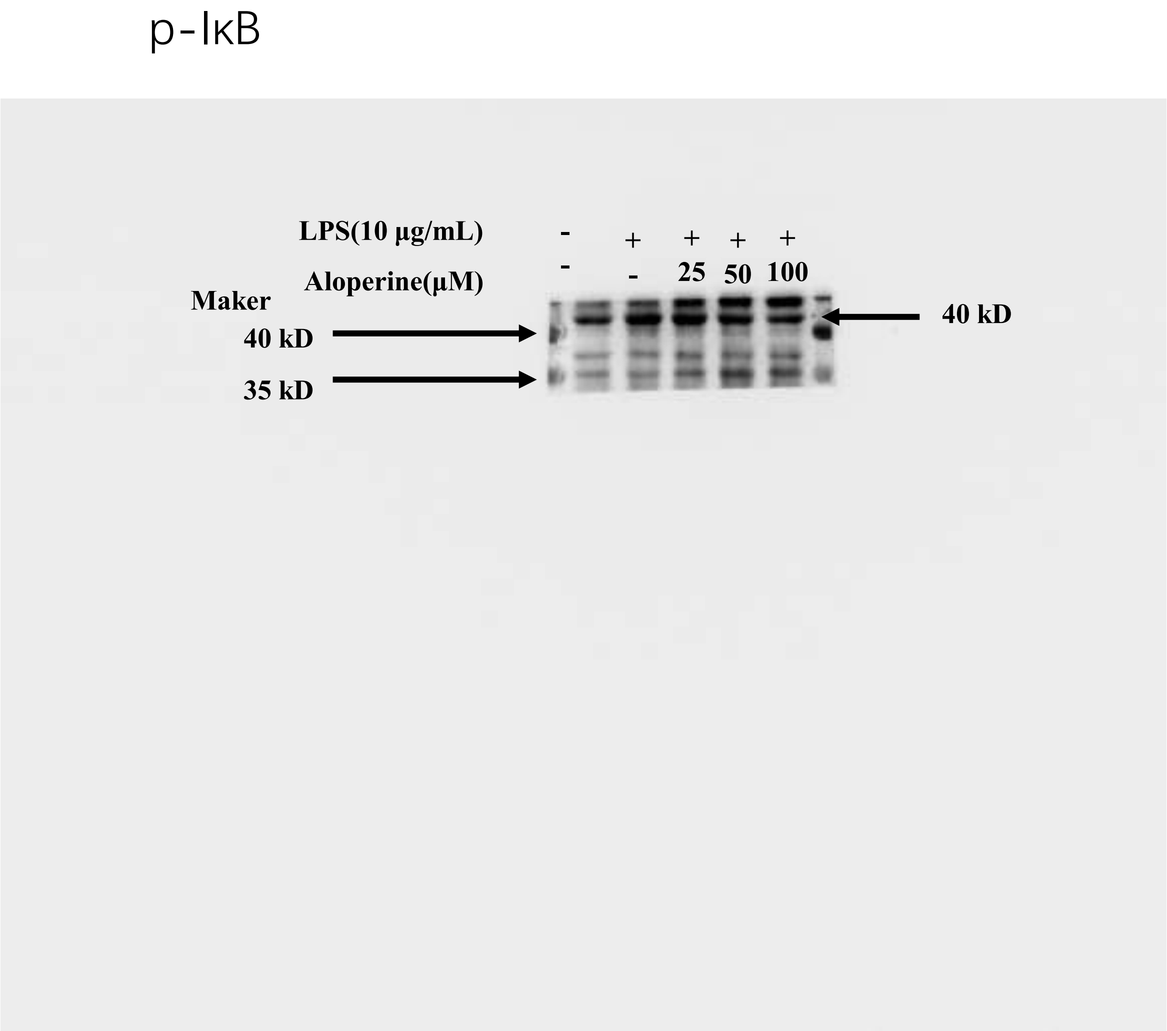
p-IκB
LPS(10 μg/mL)
Aloperine(μM)
- + + + +
- - 25 50 100
Maker
40 kD
35 kD
40 kD

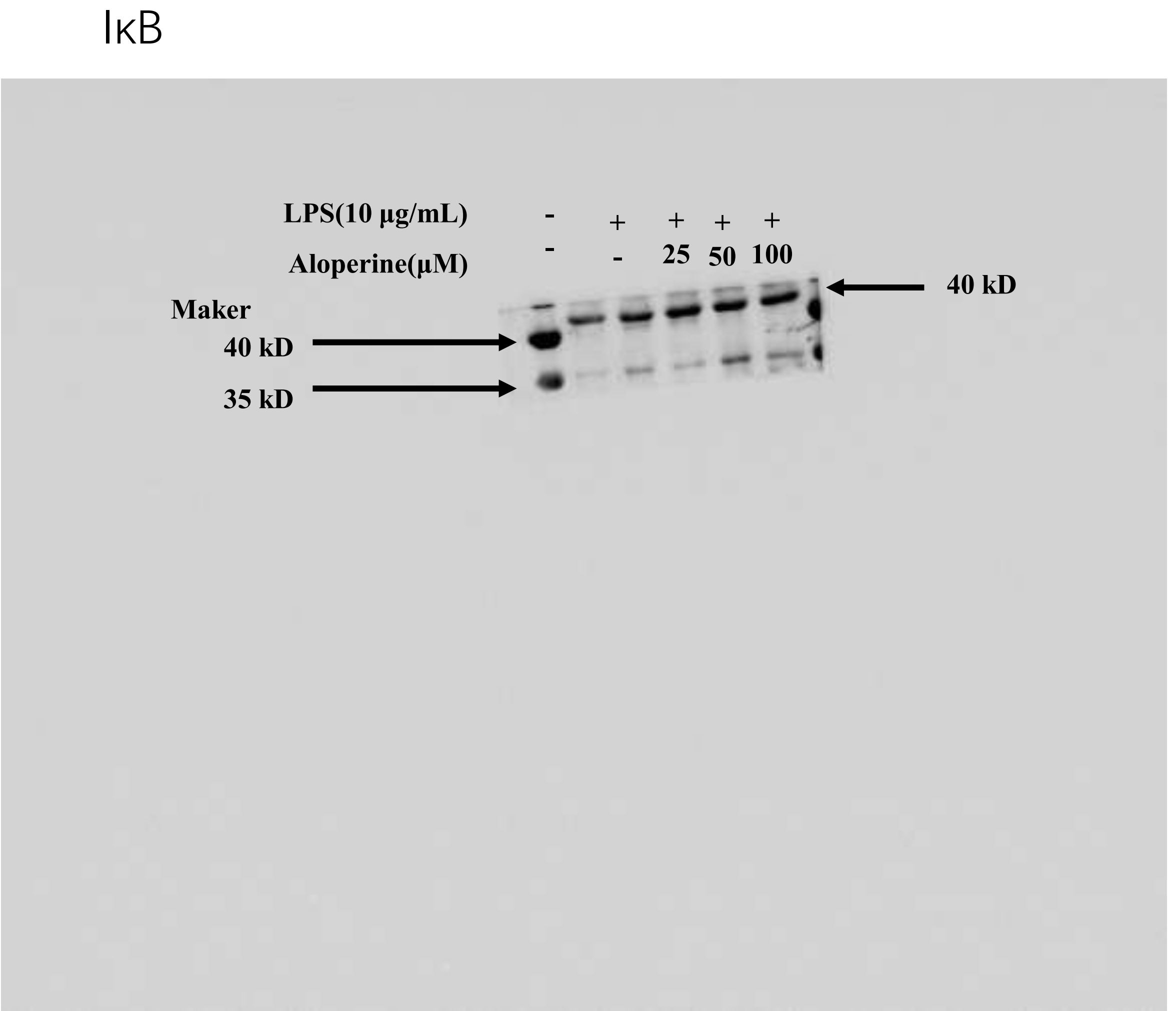
IκB
LPS(10 μg/mL)
Aloperine(μM)
- + + + +
- - 25 50 100
Maker
40 kD
35 kD
40 kD

Figure 4

MyD88

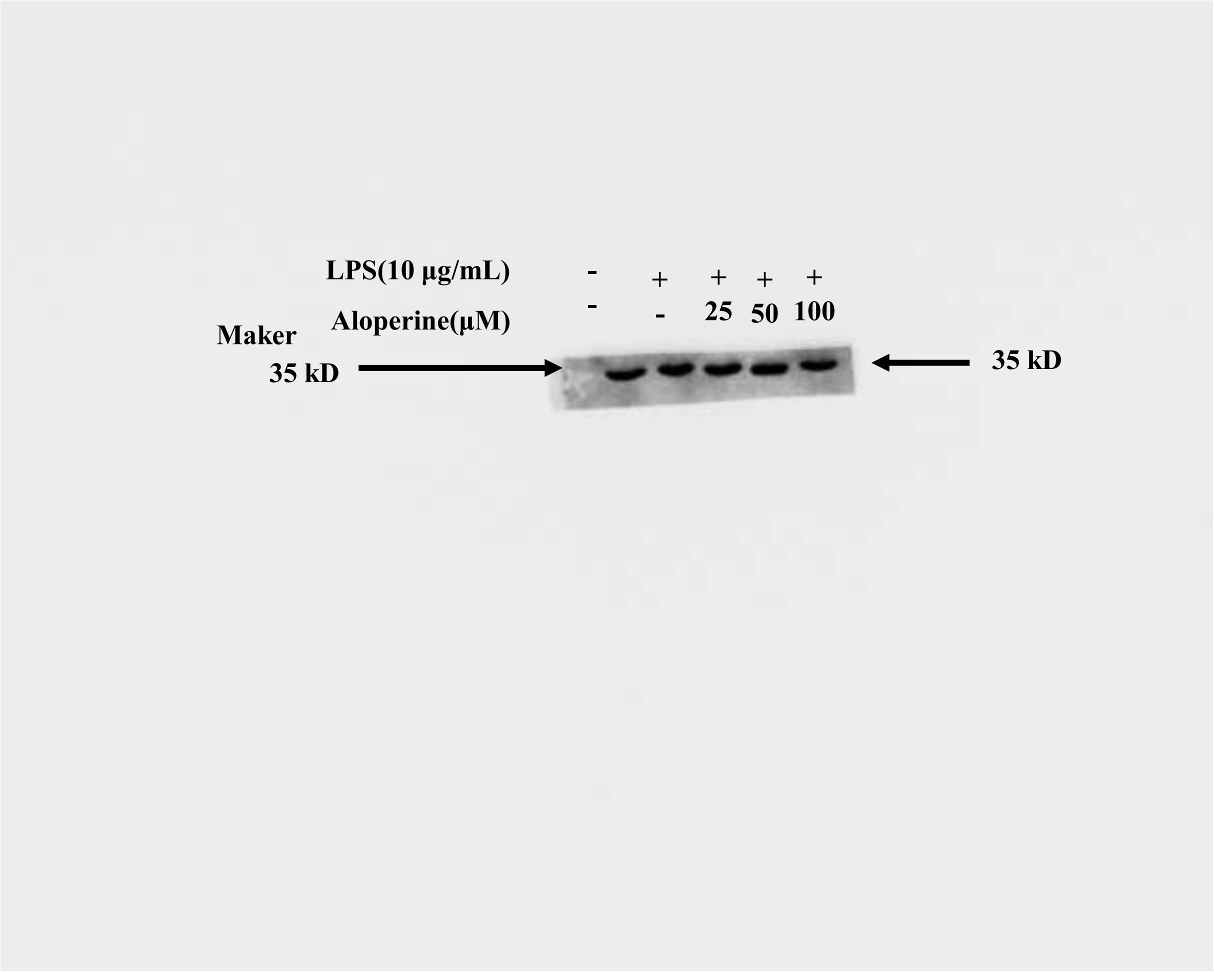

β-actin

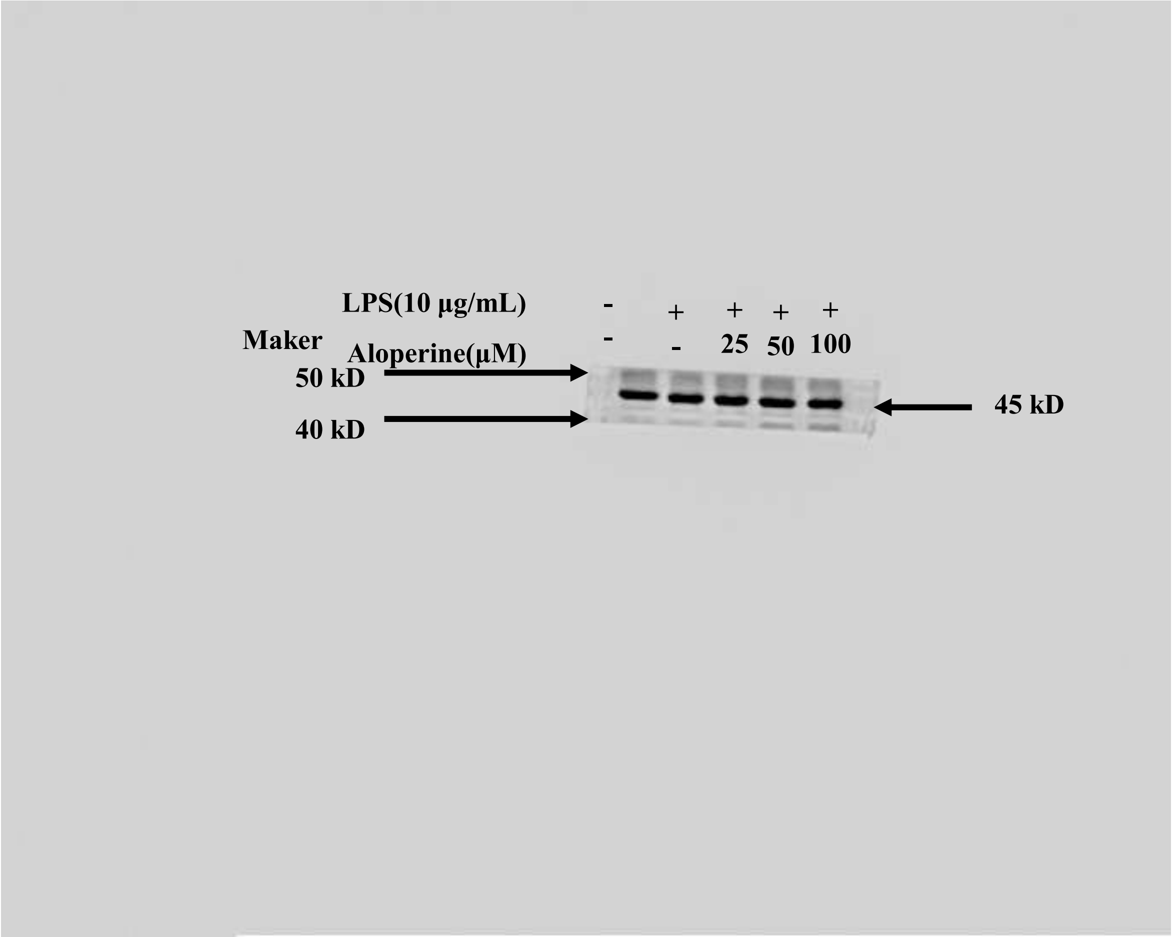

Figure 4

IKKβ

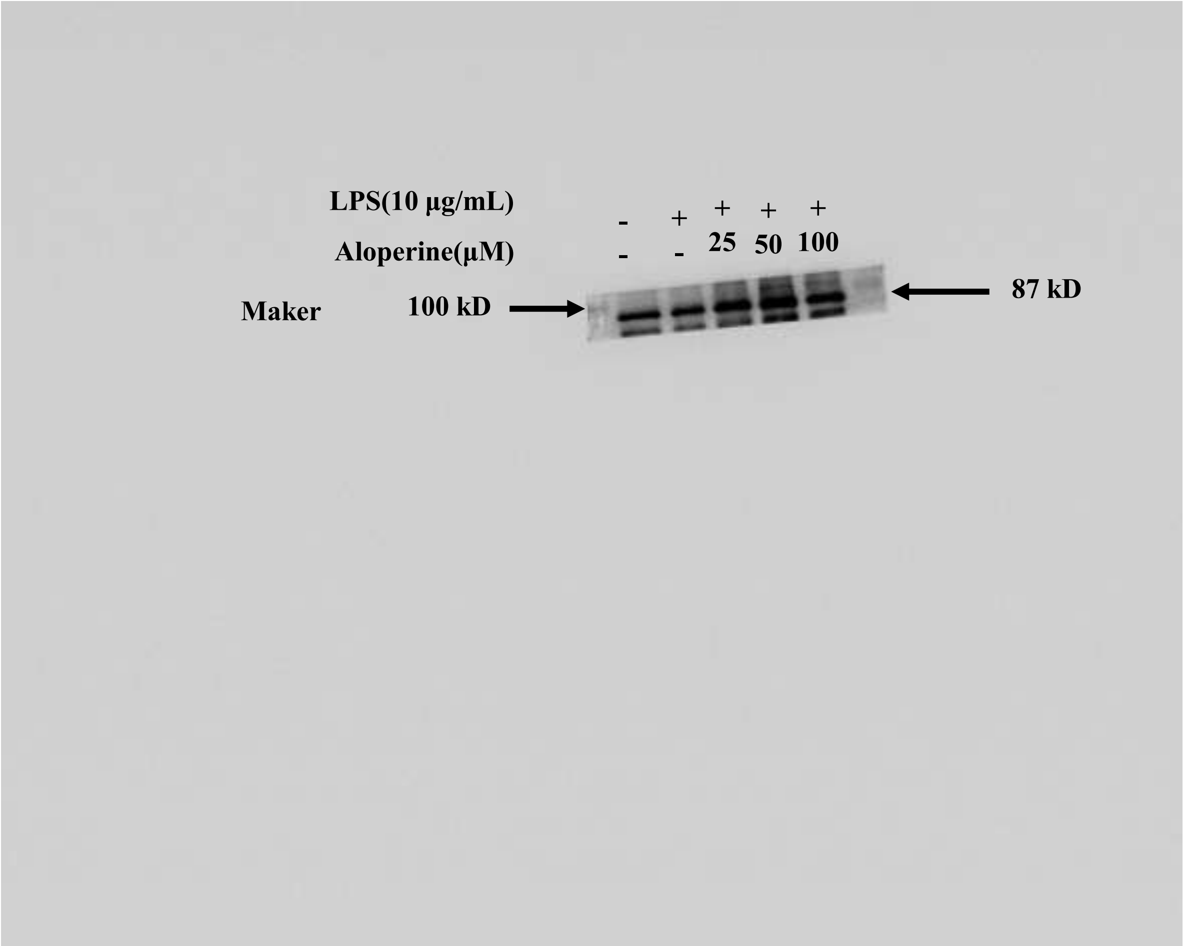

TLR4

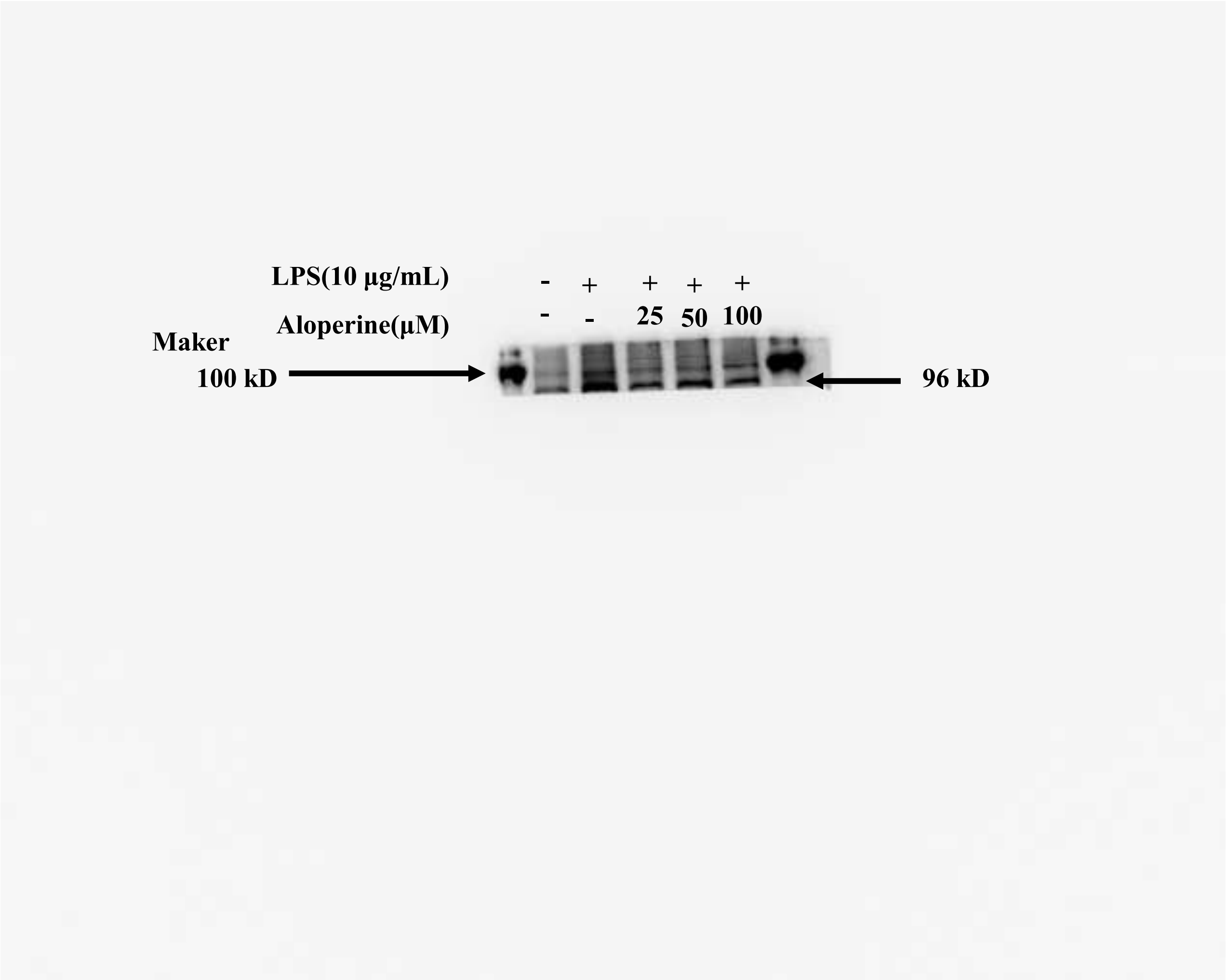

Figure 5

Beclin 1

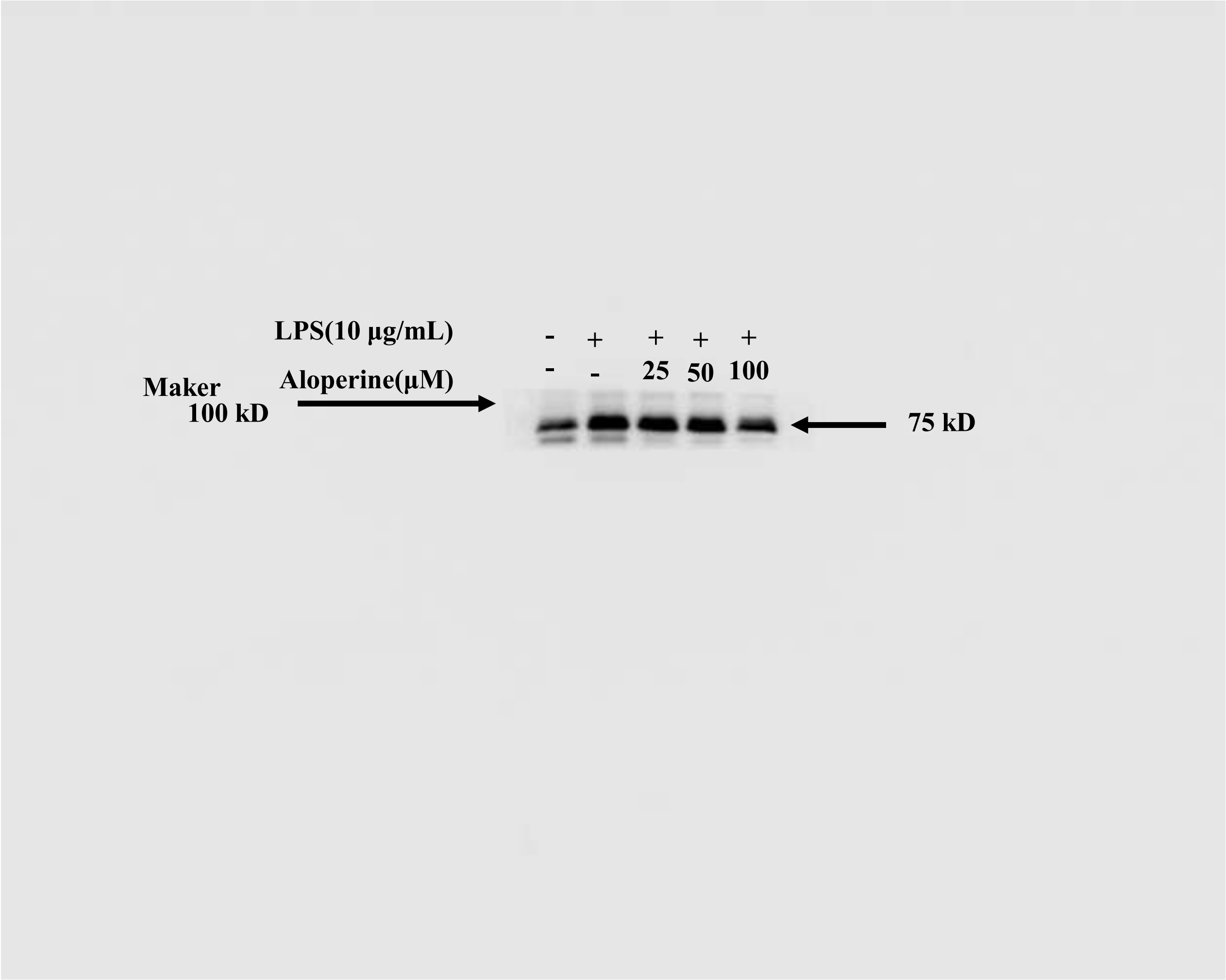

LC3

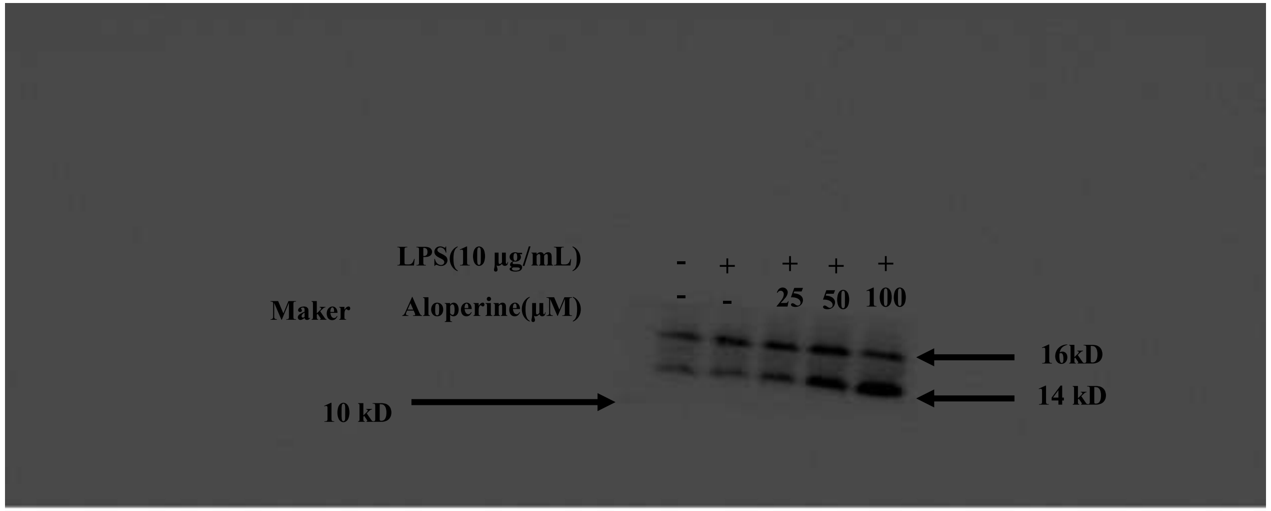

Figure 5

p62

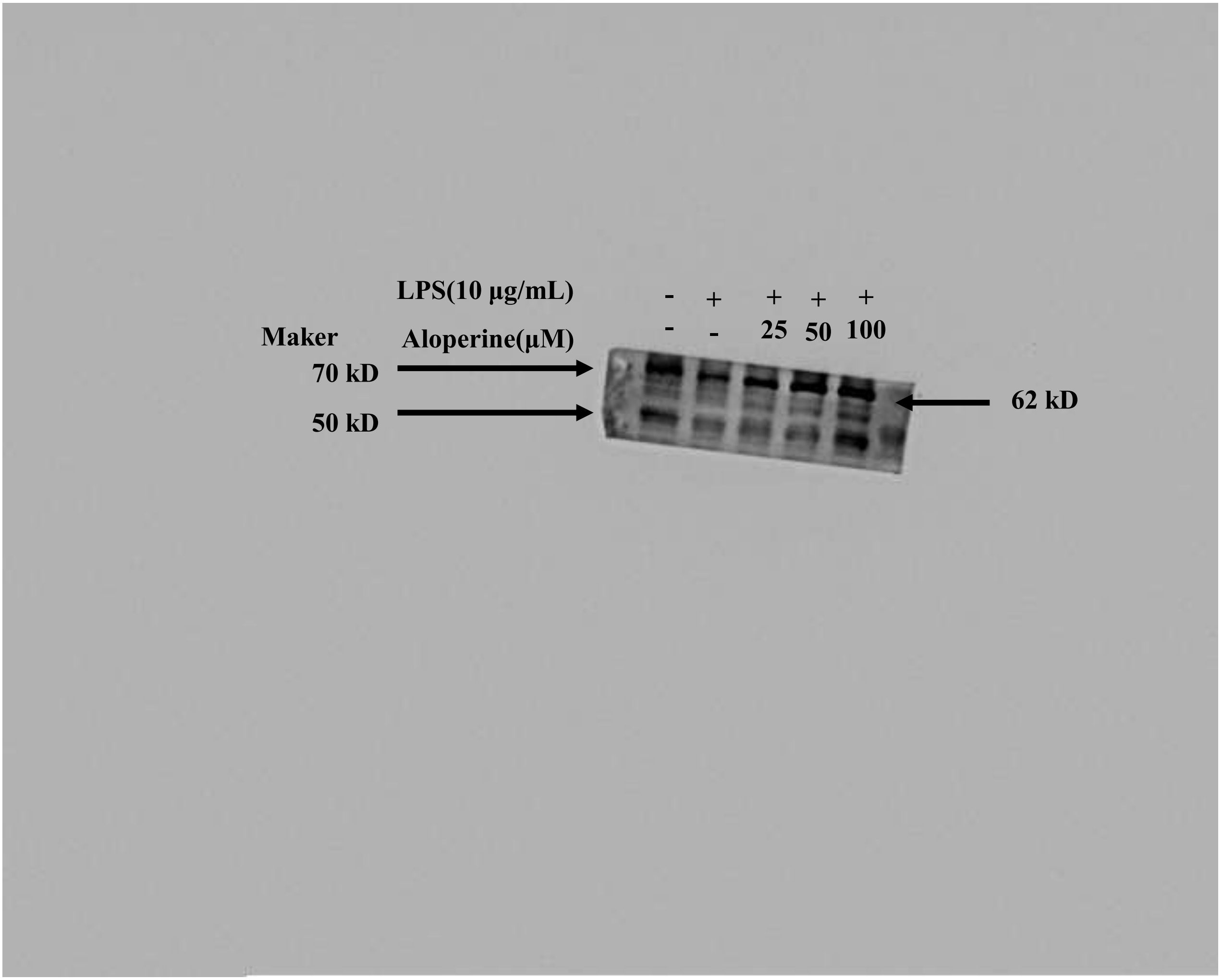

β-actin

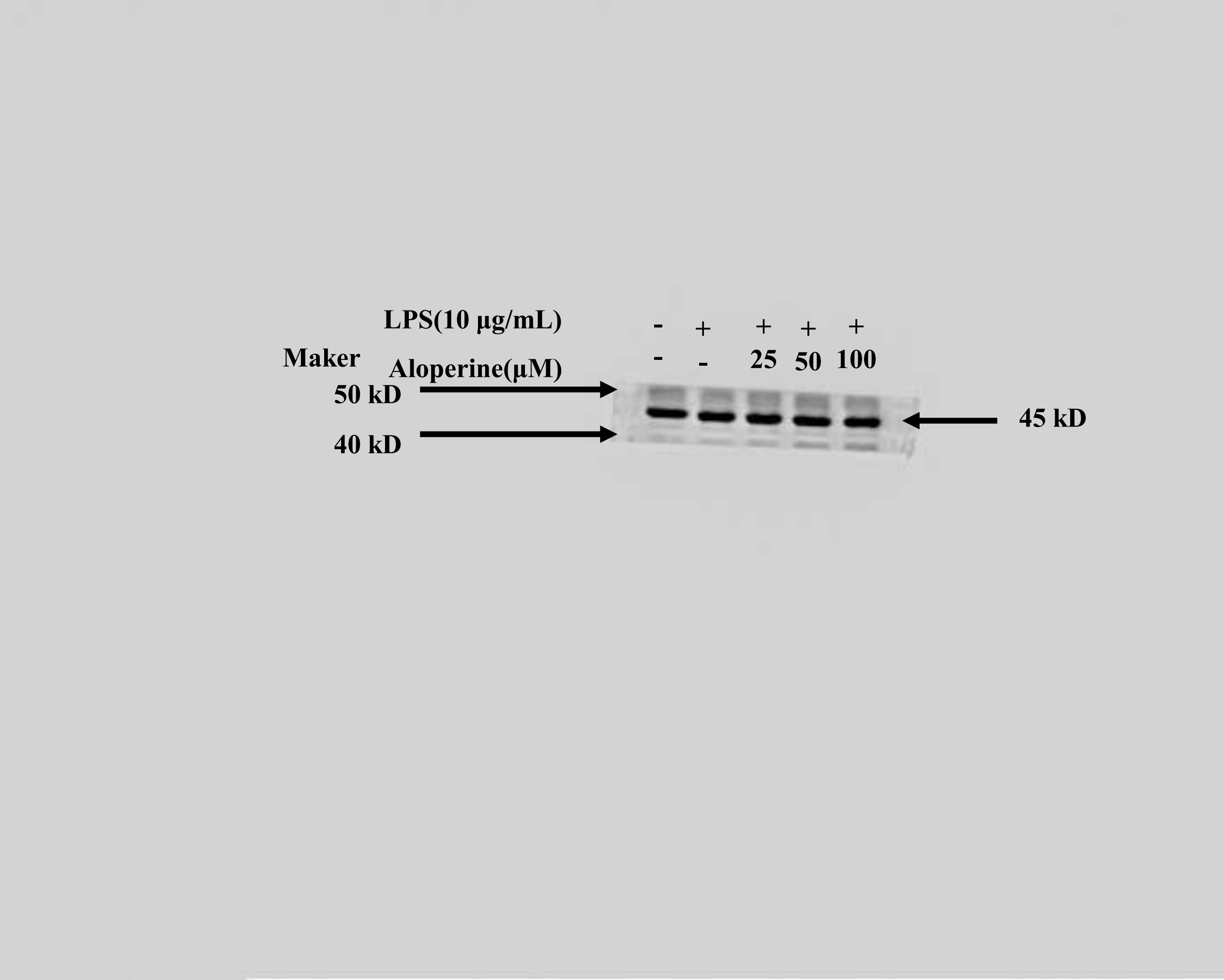

Figure 5

LAMP2A

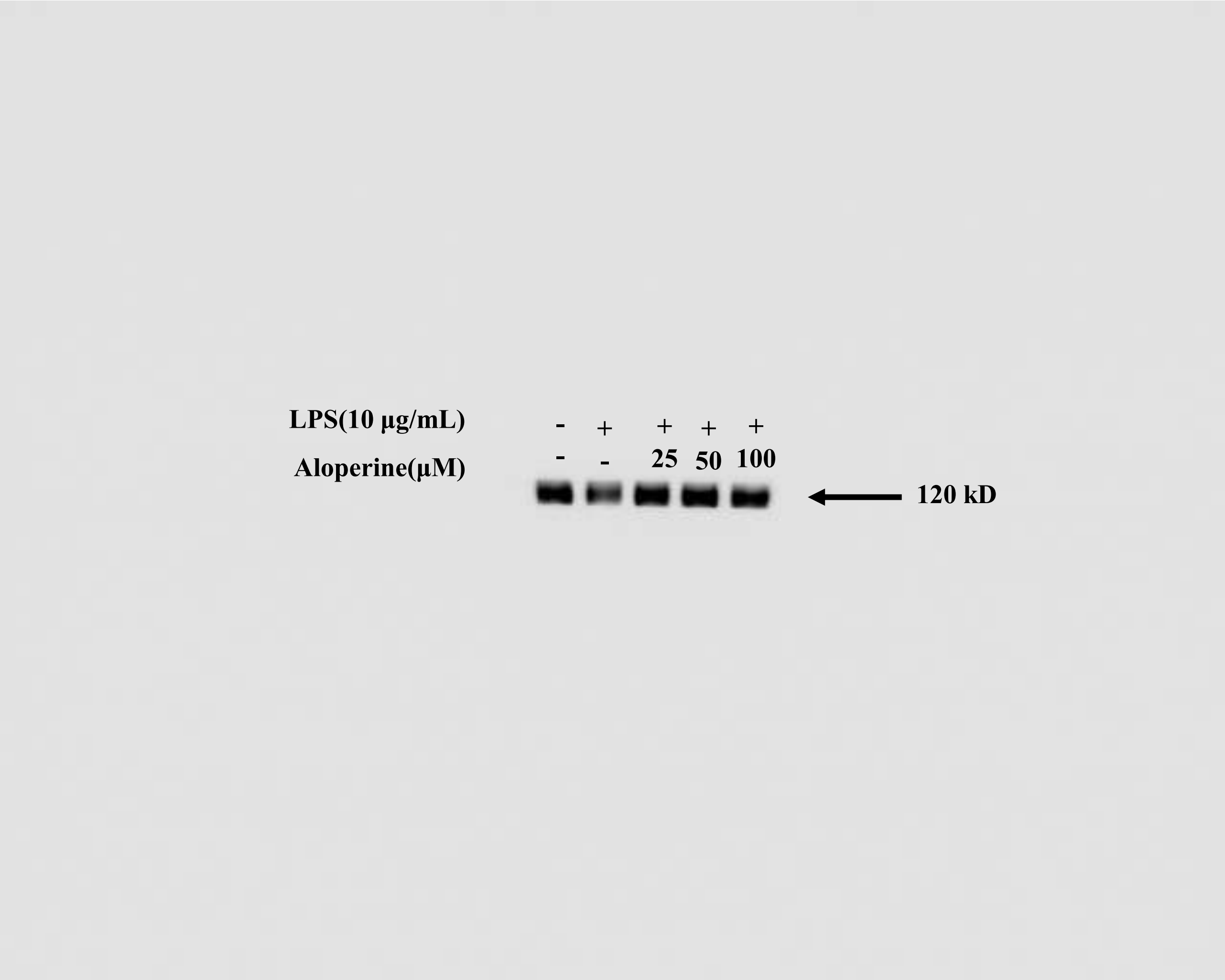

ATG7

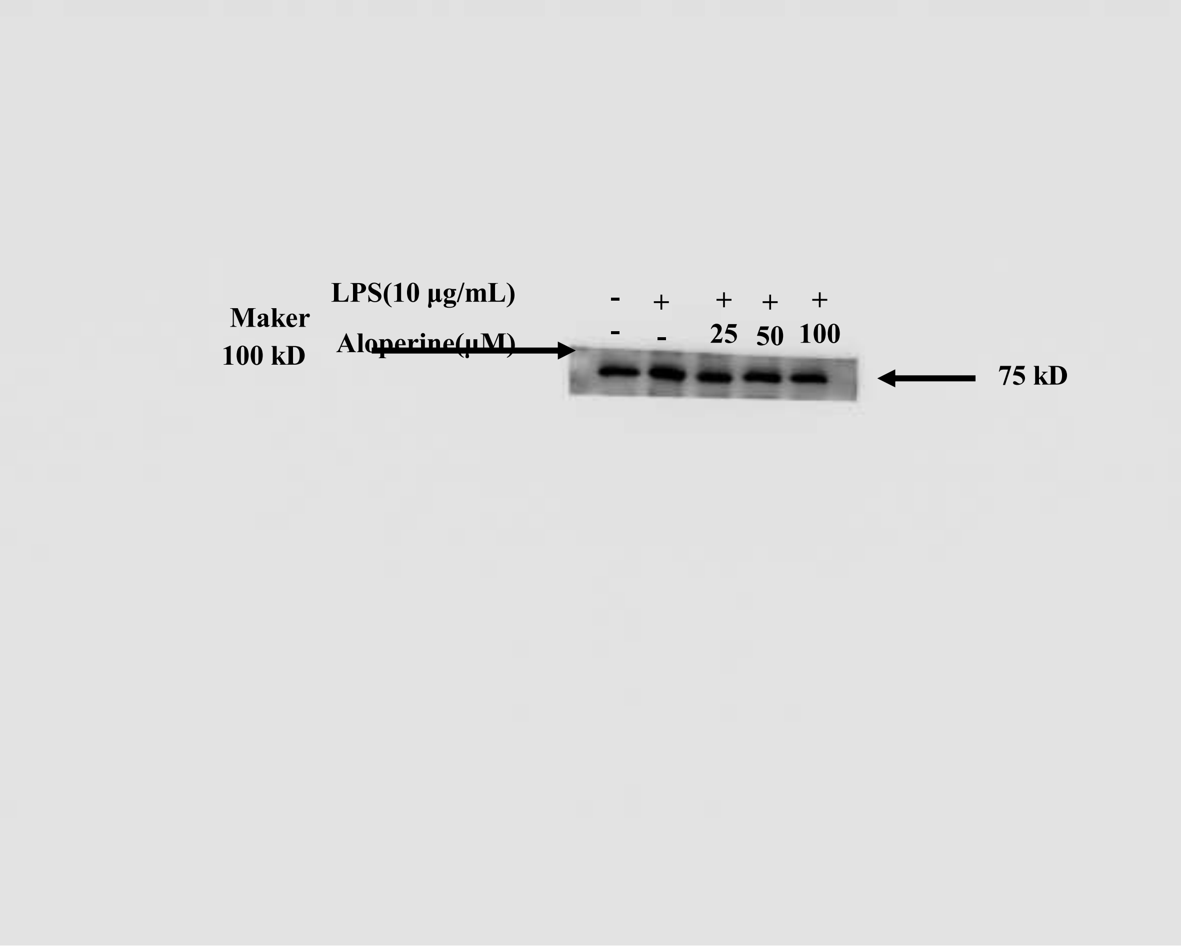

Figure 6

p62

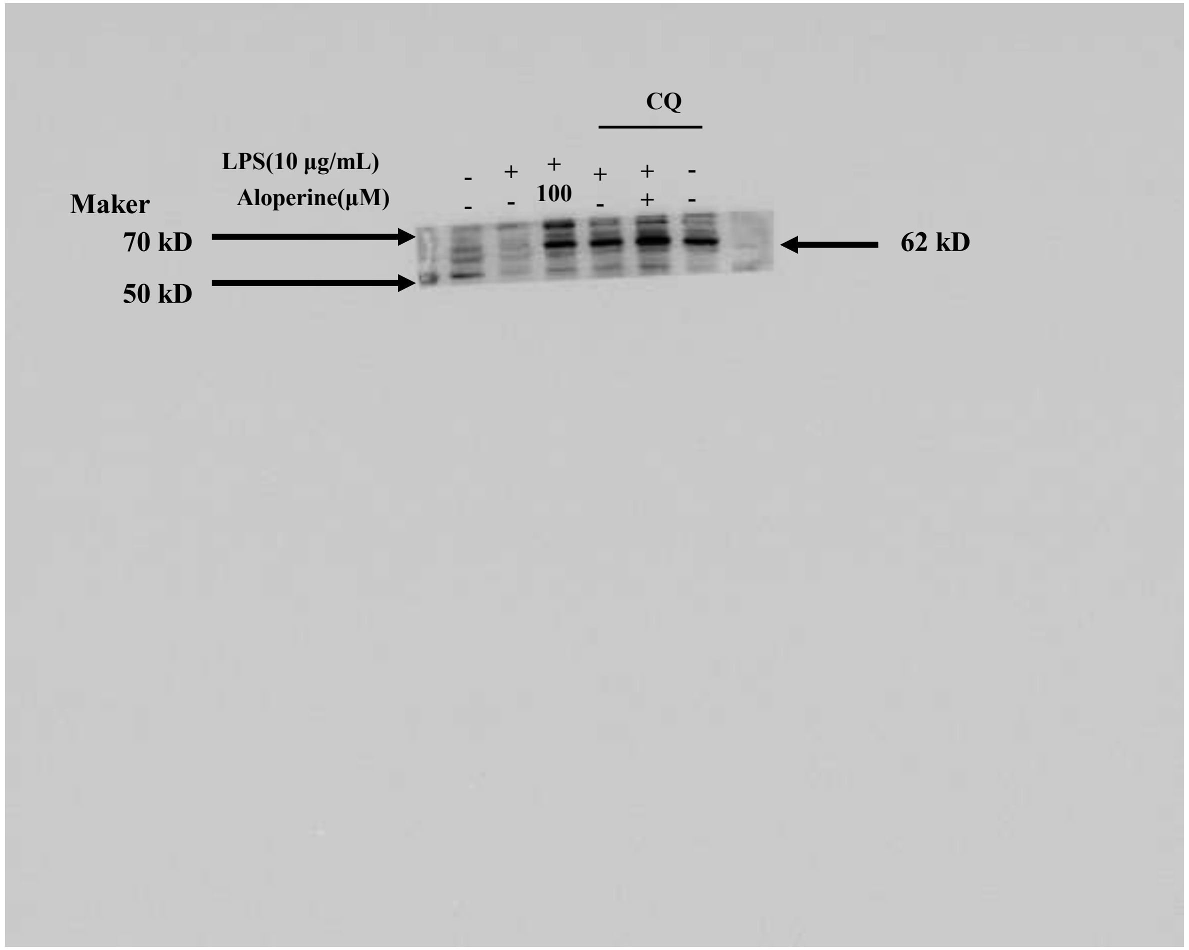

LC3

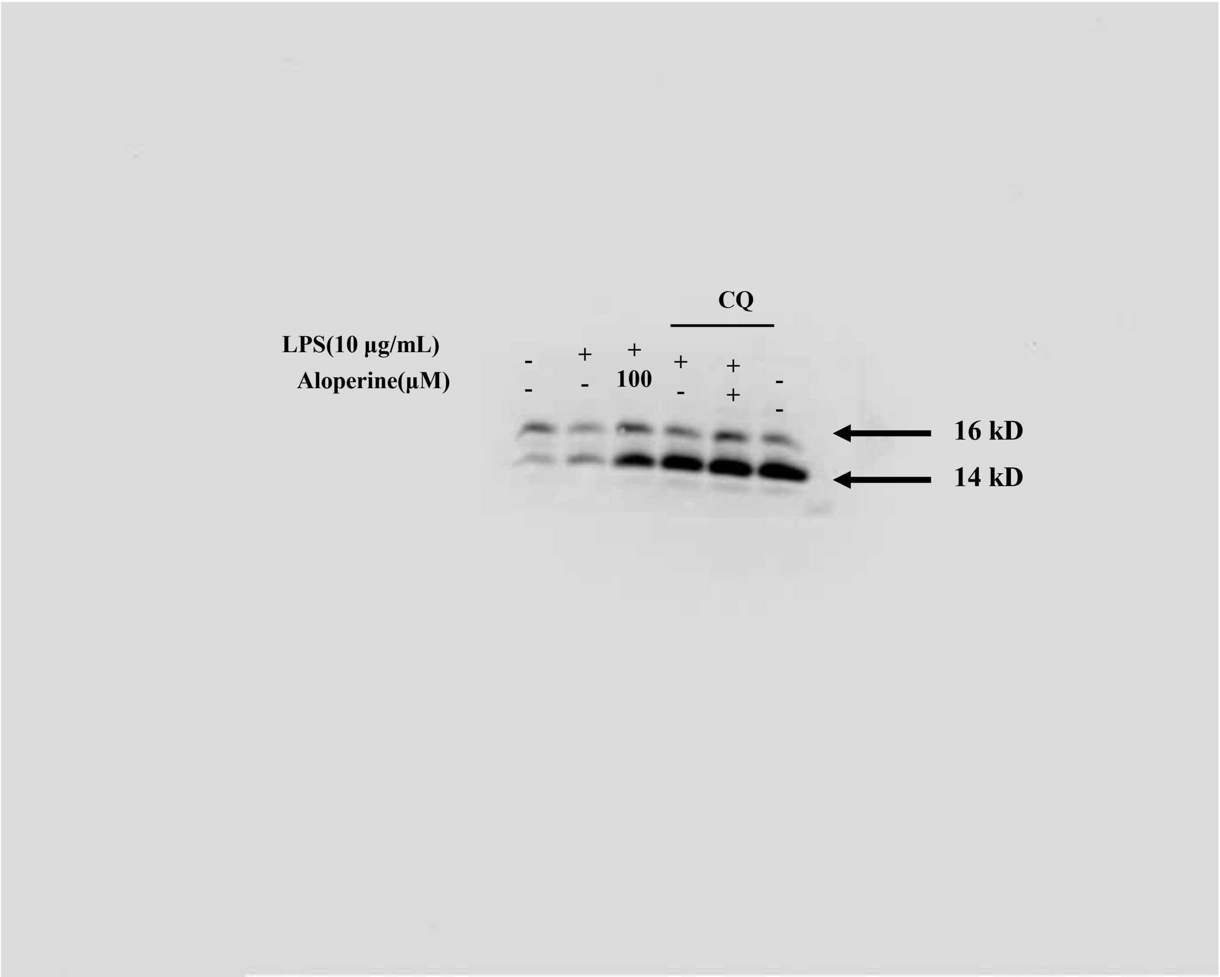

Figure 6

LAMP2A

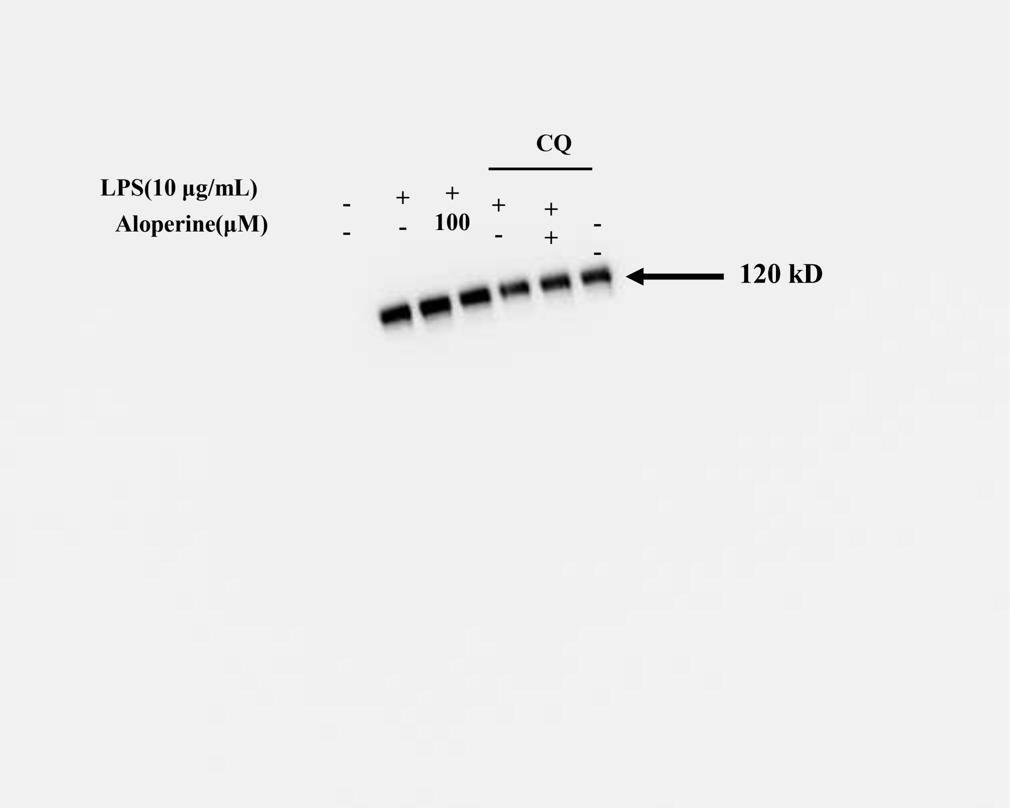

Occludin

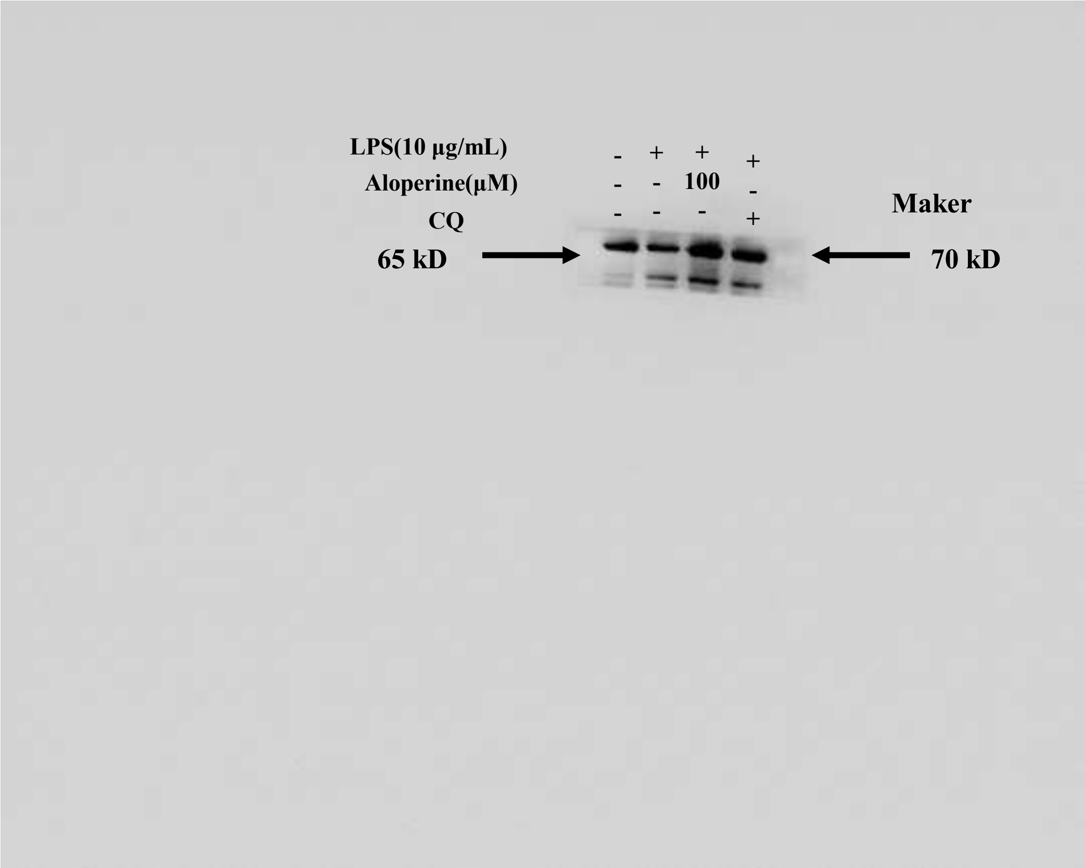

Figure 6

ZO-1

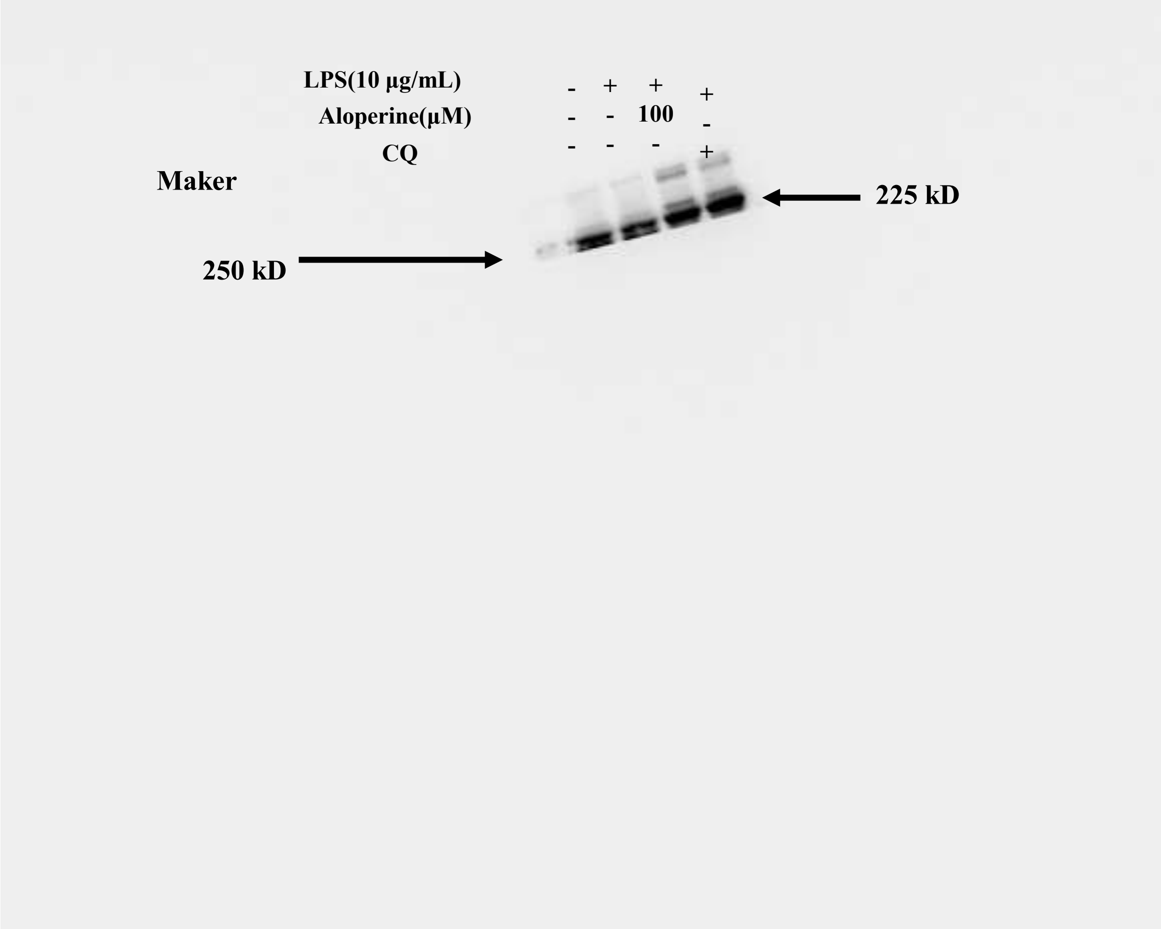

β-actin

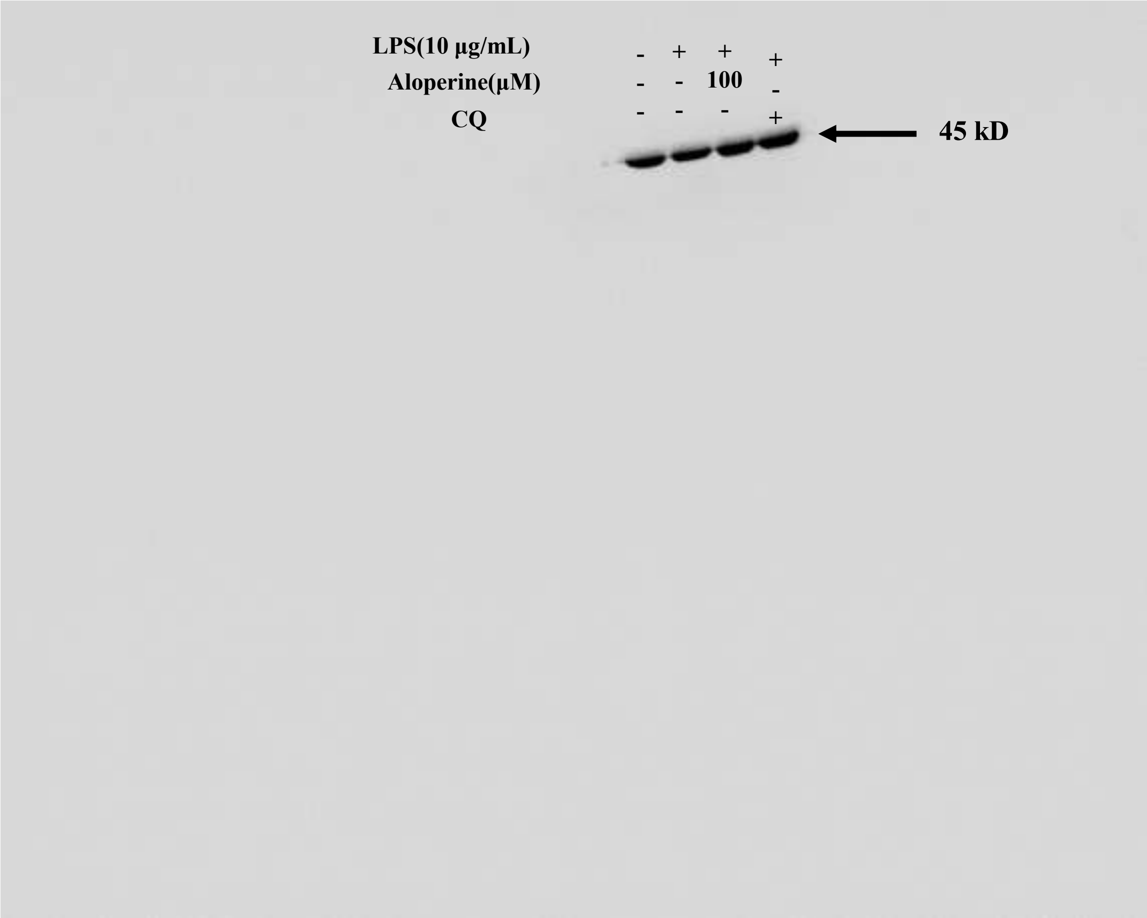

Figure 7

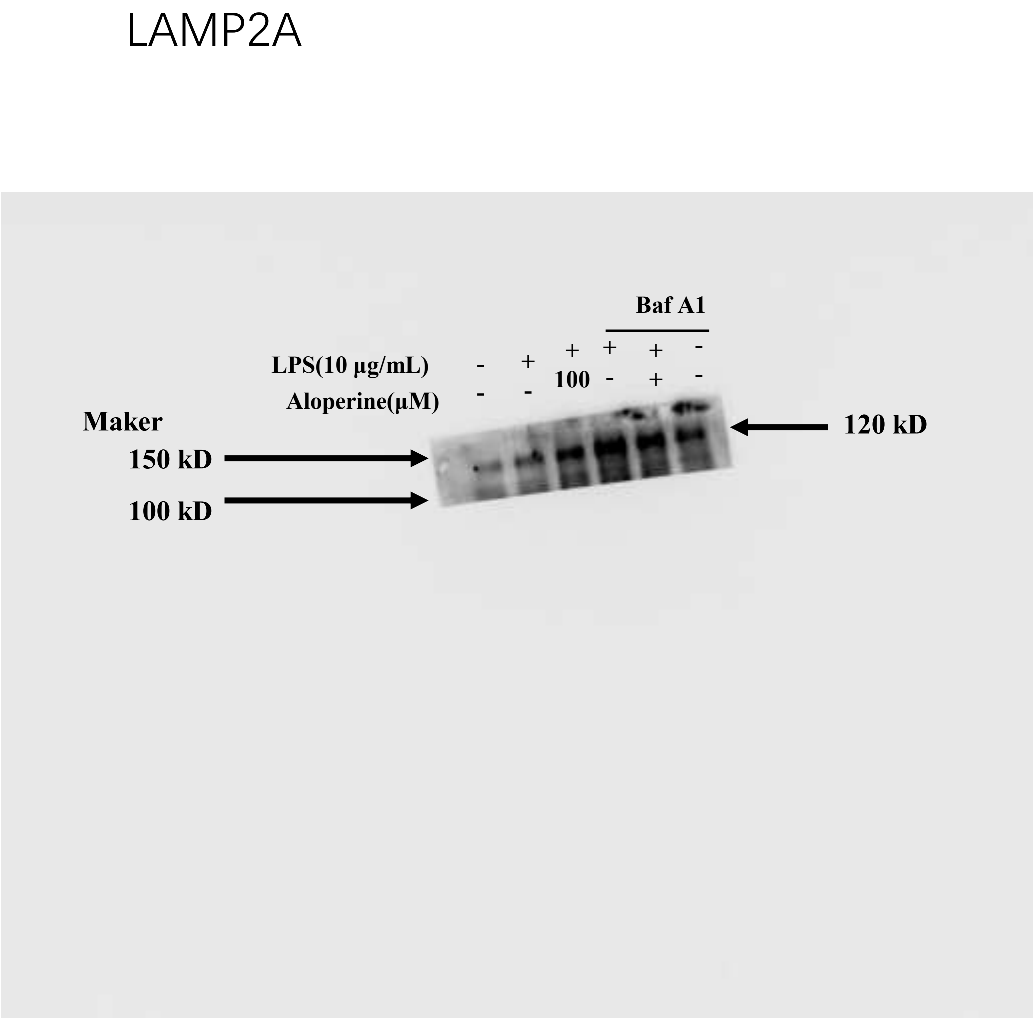
LAMP2A
Baf A1
LPS(10 μg/mL)
Aloperine(μM)
- + + + + -
- - 100 - + -
Maker
150 kD
100 kD
120 kD

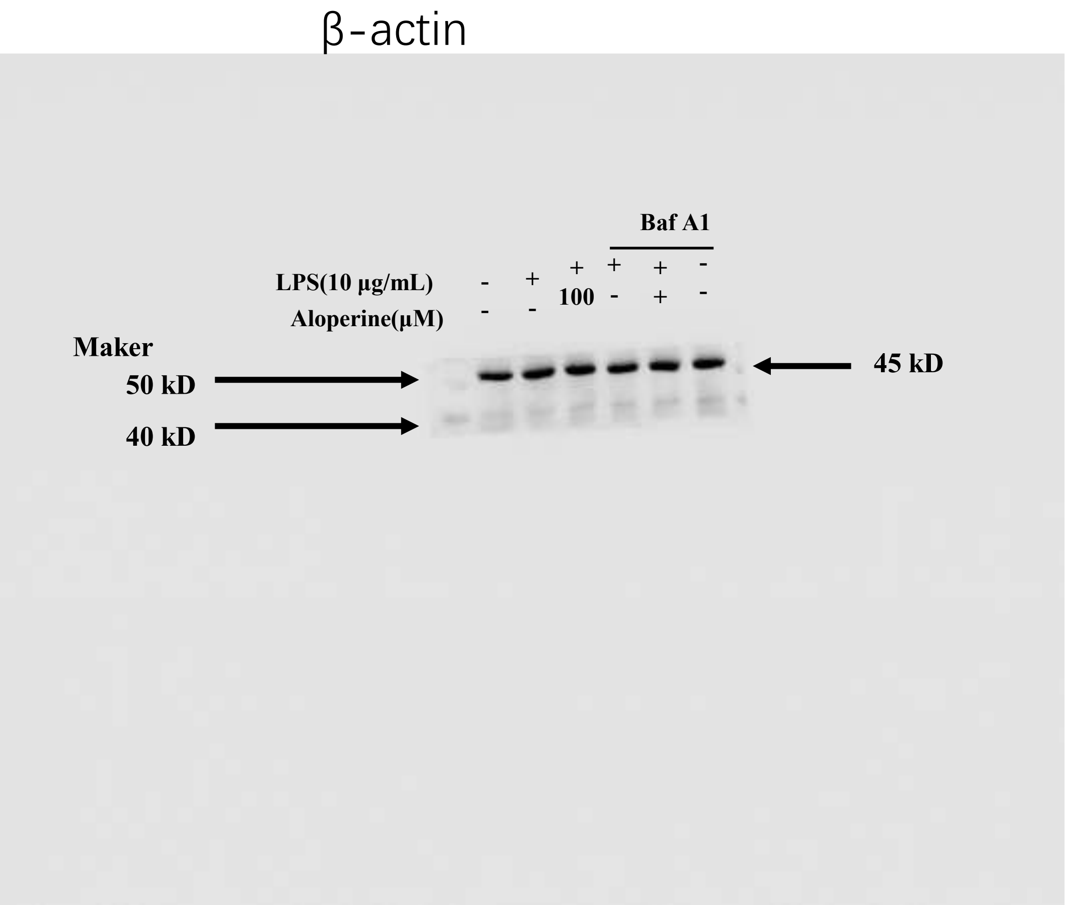
β-actin
Baf A1
LPS(10 μg/mL)
Aloperine(μM)
- + + + + -
- - 100 - + -
Maker
50 kD
40 kD
45 kD

Figure 7

p62

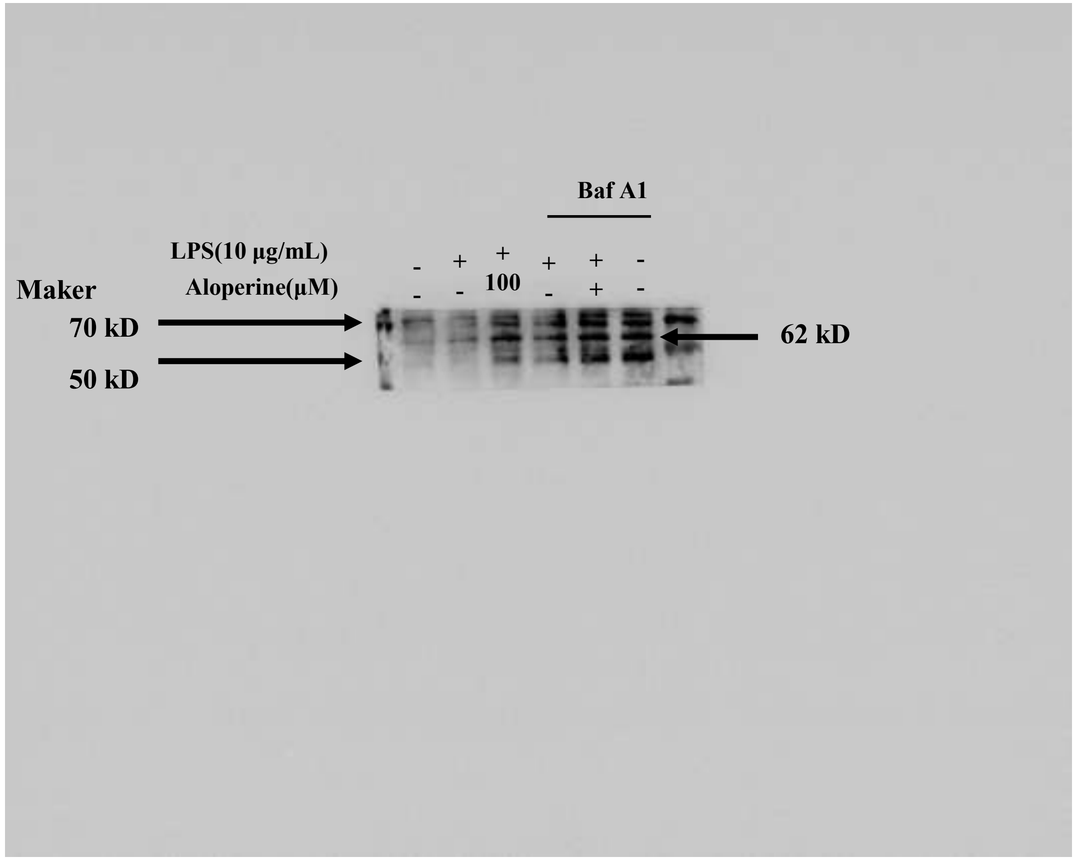

LC3

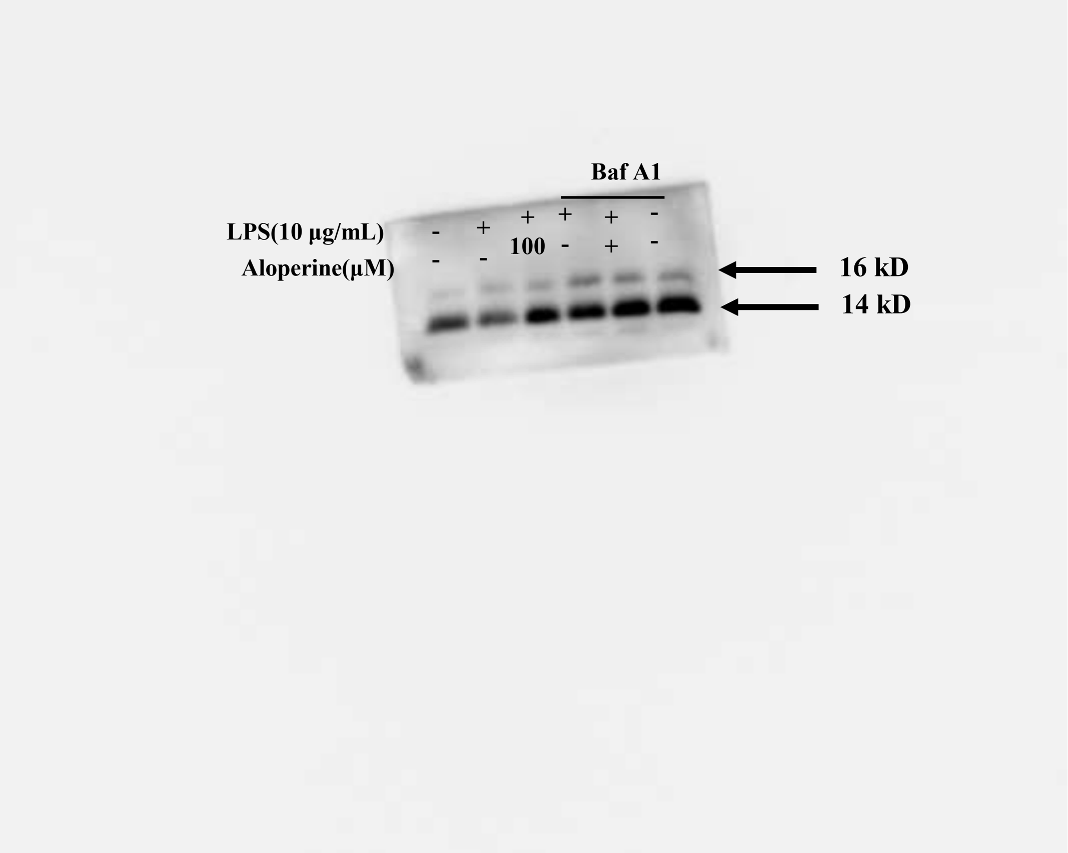

Figure 7

ZO-1

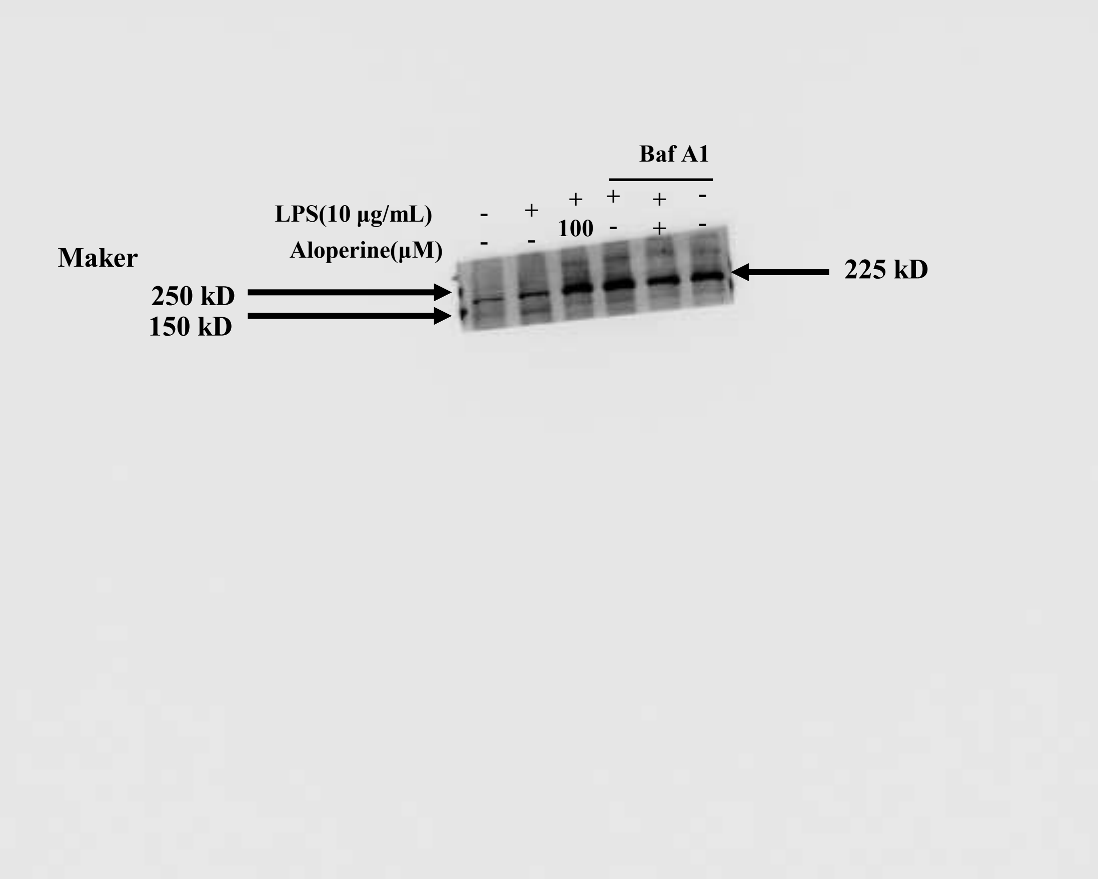

Claudin 1

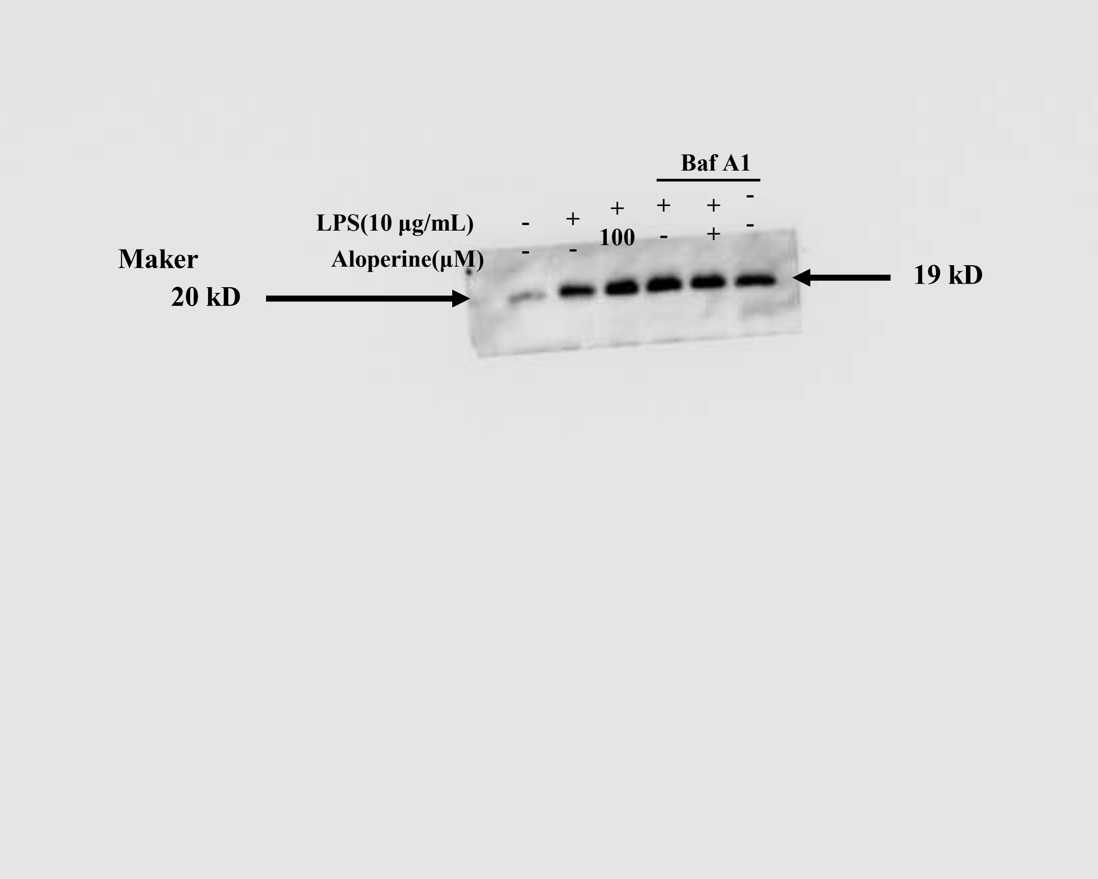

Figure 5

Occludin

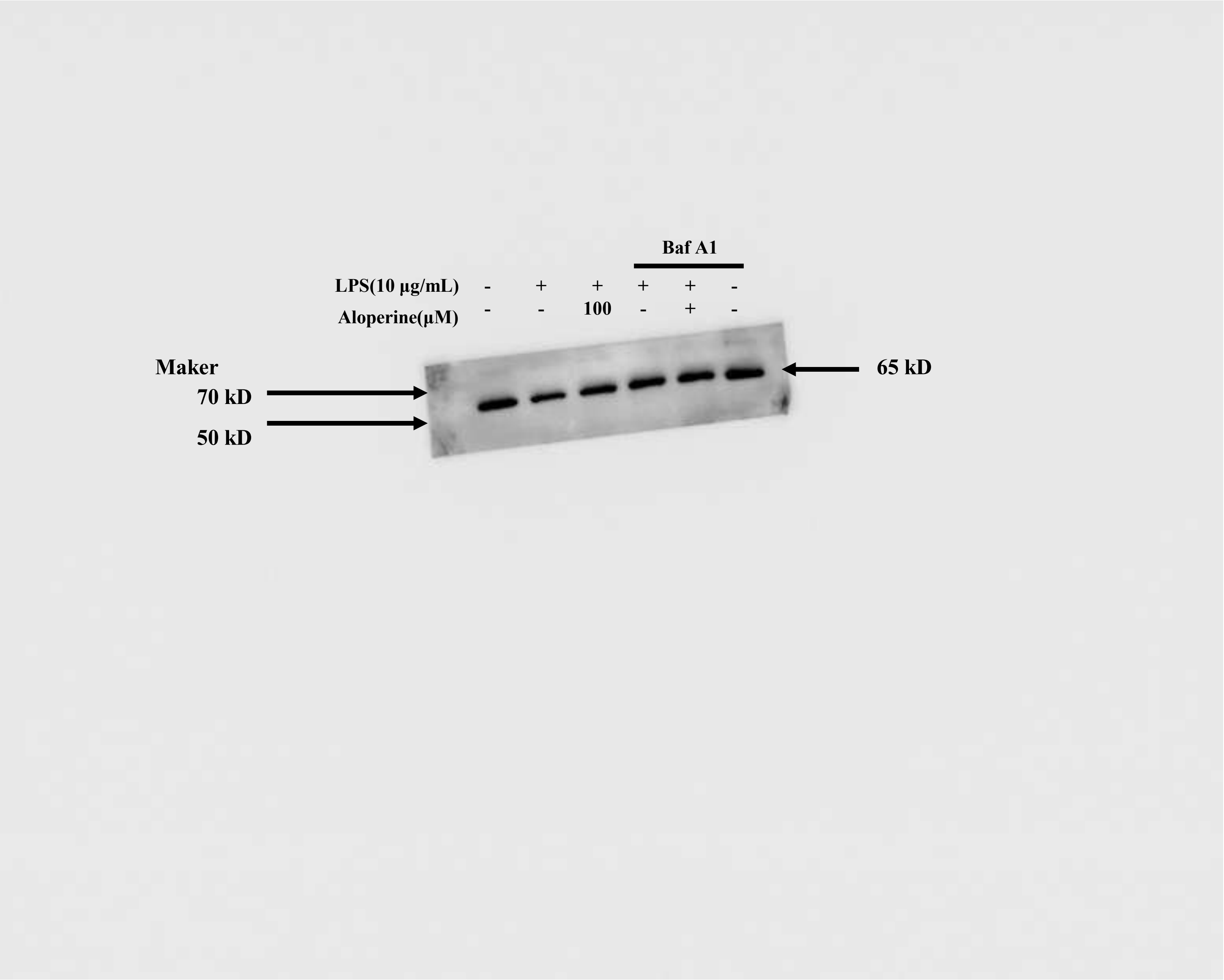

Supplement: Supplementary file 1 — Supplementary Material 1. [file 12917_2026_5337_MOESM1_ESM.pdf]
