# Supplementary material for: Aloperine alleviates LPS-induced inflammation in bovine intestinal epithelial cells through autophagy and TLR4/p38 MAPK/NF-κB pathway
Source: BMC Vet Res. 2026 Feb 20;22:193. doi: 10.1186/s12917-026-05337-7 (PMC13032521; doi:10.1186/s12917-026-05337-7)
Supplement: Supplementary file 2 — Supplementary Material 2. [file 12917_2026_5337_MOESM2_ESM.docx]

| **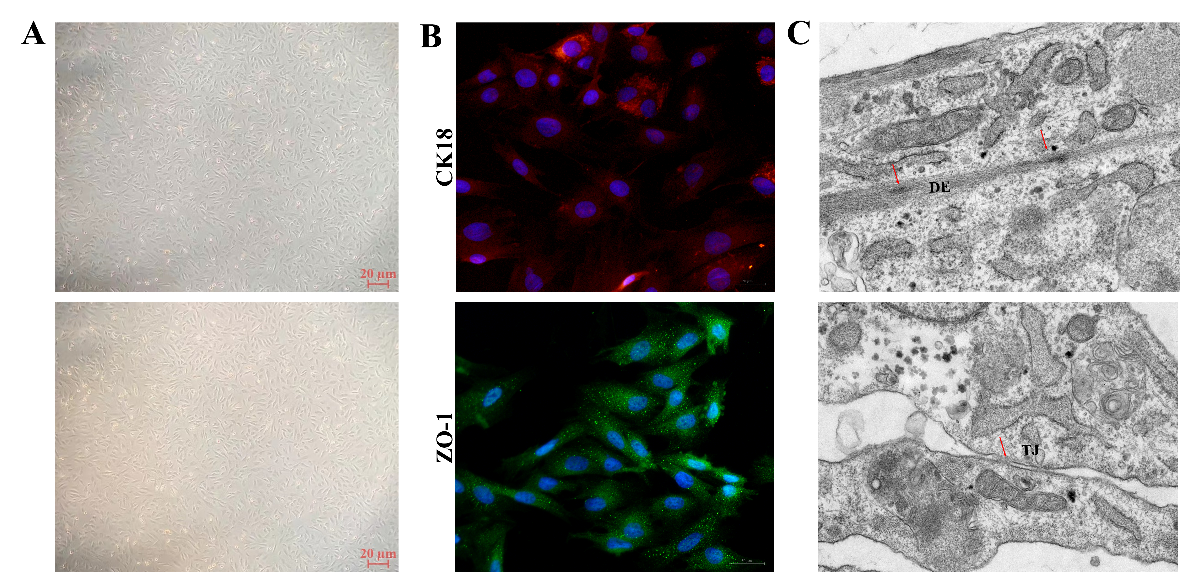**  **Figure S1 BIECs-21 cell culture and identification**  **A** The growth of BIECs-21 cells; Scale bars: 20 μm. **B** Immunofluorescence identification for BIECs-21 cells; Scale bars: 50 μm. **C** Transmission electron microscopy identification for BIECs-21 cells; Scale bars: 500 nm; DE: Desmosomes; TJ: Tight junctions. |
| --- |

**Table S1 targets for Alo and inflammation**

| **Targets name** | | | | | |
| --- | --- | --- | --- | --- | --- |
| IL6 | CTSG | ADA | CTSC | CYP19A1 | PDE4B |
| ALB | AKT1 | MMP13 | PTEN | CYP2C19 | PLA2G1B |
| JAK1 | NR1H4 | IGF1 | GSK3B | PGR | PRKCE |
| CCR5 | TYK2 | PLAT | YARS1 | HSP90AA1 | ROCK1 |
| CASP1 | NR3C1 | TACR1 | JAK3 | F10 | ROCK2 |
| FLG | PRKCD | AR | PARP1 | CDK1 | PIM2 |
| F2 | VDR | ESR1 | CHRNB2 | ESR2 | SERT |
| MMP3 | CASP8 | ARG1 | CHRNA4 | GABRA1 | KIT |
| PRKCQ | PRSS1 | LCK | TNFSF10 | GABRB2 |  |
| PTGS1 | JAK2 | SRC | NAT1 | GABRG2 |  |
| EGFR | MMP8 | LTA4H | F11 | MAPKAPK2 |  |
| MAPK14 | ADAM17 | NR1I2 | F7 | CELA1 |  |

**Table S2 GO enrichment analysis of Network pharmacy (TOP100)**

|  | **ID** | **Class** | **Descrption** | **Num** | **P-value** | **Q-value** |
| --- | --- | --- | --- | --- | --- | --- |
| 1 | GO:0010033 | Biological Process | response to organic substance | 34 | 5.25E-16 | 8.58E-13 |
| 2 | GO:0043067 | Biological Process | regulation of programmed cell death | 26 | 5.69E-16 | 8.58E-13 |
| 3 | GO:0050896 | Biological Process | response to stimulus | 58 | 1.00E-15 | 1.01E-12 |
| 4 | GO:0010941 | Biological Process | regulation of cell death | 26 | 3.44E-15 | 2.51E-12 |
| 5 | GO:0042981 | Biological Process | regulation of apoptotic process | 25 | 4.17E-15 | 2.51E-12 |
| 6 | GO:0012501 | Biological Process | programmed cell death | 27 | 6.07E-15 | 2.77E-12 |
| 7 | GO:0051716 | Biological Process | cellular response to stimulus | 54 | 6.42E-15 | 2.77E-12 |
| 8 | GO:1901700 | Biological Process | response to oxygen-containing compound | 23 | 1.67E-14 | 6.29E-12 |
| 9 | GO:0065008 | Biological Process | regulation of biological quality | 37 | 2.88E-14 | 7.46E-12 |
| 10 | GO:0006915 | Biological Process | apoptotic process | 26 | 2.89E-14 | 7.46E-12 |
| 11 | GO:0009725 | Biological Process | response to hormone | 18 | 3.26E-14 | 7.46E-12 |
| 12 | GO:0033993 | Biological Process | response to lipid | 18 | 3.26E-14 | 7.46E-12 |
| 13 | GO:0008219 | Biological Process | cell death | 27 | 3.46E-14 | 7.46E-12 |
| 14 | GO:0016265 | Biological Process | death | 27 | 3.46E-14 | 7.46E-12 |
| 15 | GO:0009719 | Biological Process | response to endogenous stimulus | 24 | 4.28E-14 | 8.60E-12 |
| 16 | GO:0014070 | Biological Process | response to organic cyclic compound | 18 | 5.26E-14 | 9.92E-12 |
| 17 | GO:0070887 | Biological Process | cellular response to chemical stimulus | 32 | 7.38E-14 | 1.29E-11 |
| 18 | GO:0035556 | Biological Process | intracellular signal transduction | 31 | 7.68E-14 | 1.29E-11 |
| 19 | GO:0007154 | Biological Process | cell communication | 50 | 1.00E-13 | 1.59E-11 |
| 20 | GO:0071310 | Biological Process | cellular response to organic substance | 29 | 1.16E-13 | 1.75E-11 |
| 21 | GO:0007165 | Biological Process | signal transduction | 48 | 2.24E-13 | 3.21E-11 |
| 22 | GO:0043167 | Molecular Function | ion binding | 47 | 1.56E-13 | 3.33E-11 |
| 23 | GO:0004672 | Molecular Function | protein kinase activity | 19 | 1.67E-13 | 3.33E-11 |
| 24 | GO:0044700 | Biological Process | single organism signaling | 49 | 3.77E-13 | 5.17E-11 |
| 25 | GO:0023052 | Biological Process | signaling | 49 | 4.10E-13 | 5.38E-11 |
| 26 | GO:0044707 | Biological Process | single-multicellular organism process | 44 | 4.45E-13 | 5.59E-11 |
| 27 | GO:0048584 | Biological Process | positive regulation of response to stimulus | 28 | 6.44E-13 | 7.77E-11 |
| 28 | GO:0071396 | Biological Process | cellular response to lipid | 15 | 8.44E-13 | 9.79E-11 |
| 29 | GO:1901701 | Biological Process | cellular response to oxygen-containing compound | 19 | 1.18E-12 | 1.32E-10 |
| 30 | GO:0048518 | Biological Process | positive regulation of biological process | 45 | 2.09E-12 | 2.26E-10 |
| 31 | GO:0016773 | Molecular Function | phosphotransferase activity, alcohol group as acceptor | 19 | 3.25E-12 | 4.31E-10 |
| 32 | GO:0032870 | Biological Process | cellular response to hormone stimulus | 15 | 5.86E-12 | 5.92E-10 |
| 33 | GO:0006468 | Biological Process | protein phosphorylation | 26 | 5.89E-12 | 5.92E-10 |
| 34 | GO:0018193 | Biological Process | peptidyl-amino acid modification | 22 | 8.48E-12 | 8.25E-10 |
| 35 | GO:0097305 | Biological Process | response to alcohol | 10 | 1.14E-11 | 1.07E-09 |
| 36 | GO:0004879 | Molecular Function | RNA polymerase II transcription factor activity, ligand-activated sequence-specific DNA binding | 8 | 1.50E-11 | 1.19E-09 |
| 37 | GO:0098531 | Molecular Function | transcription factor activity, direct ligand regulated sequence-specific DNA binding | 8 | 1.50E-11 | 1.19E-09 |
| 38 | GO:0016301 | Molecular Function | kinase activity | 19 | 2.11E-11 | 1.40E-09 |
| 39 | GO:0048522 | Biological Process | positive regulation of cellular process | 41 | 1.90E-11 | 1.74E-09 |
| 40 | GO:0006950 | Biological Process | response to stress | 33 | 2.16E-11 | 1.92E-09 |
| 41 | GO:0002376 | Biological Process | immune system process | 28 | 2.32E-11 | 2.00E-09 |
| 42 | GO:0042221 | Biological Process | response to chemical | 38 | 5.62E-11 | 4.70E-09 |
| 43 | GO:0048519 | Biological Process | negative regulation of biological process | 39 | 6.46E-11 | 5.27E-09 |
| 44 | GO:0043069 | Biological Process | negative regulation of programmed cell death | 17 | 6.98E-11 | 5.54E-09 |
| 45 | GO:0051239 | Biological Process | regulation of multicellular organismal process | 29 | 7.76E-11 | 6.00E-09 |
| 46 | GO:1901698 | Biological Process | response to nitrogen compound | 16 | 9.00E-11 | 6.78E-09 |
| 47 | GO:0051240 | Biological Process | positive regulation of multicellular organismal process | 22 | 1.15E-10 | 8.44E-09 |
| 48 | GO:1902533 | Biological Process | positive regulation of intracellular signal transduction | 18 | 1.46E-10 | 1.05E-08 |
| 49 | GO:0007166 | Biological Process | cell surface receptor signaling pathway | 28 | 1.75E-10 | 1.23E-08 |
| 50 | GO:0071495 | Biological Process | cellular response to endogenous stimulus | 19 | 2.01E-10 | 1.38E-08 |
| 51 | GO:0006796 | Biological Process | phosphate-containing compound metabolic process | 31 | 2.44E-10 | 1.61E-08 |
| 52 | GO:0060548 | Biological Process | negative regulation of cell death | 17 | 2.45E-10 | 1.61E-08 |
| 53 | GO:0023056 | Biological Process | positive regulation of signaling | 22 | 3.02E-10 | 1.94E-08 |
| 54 | GO:0019538 | Biological Process | protein metabolic process | 43 | 3.18E-10 | 2.00E-08 |
| 55 | GO:0009967 | Biological Process | positive regulation of signal transduction | 21 | 3.35E-10 | 2.05E-08 |
| 56 | GO:0097306 | Biological Process | cellular response to alcohol | 8 | 3.40E-10 | 2.05E-08 |
| 57 | GO:0018108 | Biological Process | peptidyl-tyrosine phosphorylation | 12 | 3.56E-10 | 2.11E-08 |
| 58 | GO:0006793 | Biological Process | phosphorus metabolic process | 31 | 3.68E-10 | 2.13E-08 |
| 59 | GO:0018212 | Biological Process | peptidyl-tyrosine modification | 12 | 4.03E-10 | 2.29E-08 |
| 60 | GO:0016772 | Molecular Function | transferase activity, transferring phosphorus-containing groups | 19 | 4.12E-10 | 2.34E-08 |
| 61 | GO:0010647 | Biological Process | positive regulation of cell communication | 22 | 4.39E-10 | 2.45E-08 |
| 62 | GO:0018209 | Biological Process | peptidyl-serine modification | 12 | 4.55E-10 | 2.45E-08 |
| 63 | GO:0016310 | Biological Process | phosphorylation | 26 | 4.55E-10 | 2.45E-08 |
| 64 | GO:1902531 | Biological Process | regulation of intracellular signal transduction | 22 | 4.86E-10 | 2.57E-08 |
| 65 | GO:0002684 | Biological Process | positive regulation of immune system process | 17 | 5.65E-10 | 2.94E-08 |
| 66 | GO:0010243 | Biological Process | response to organonitrogen compound | 14 | 5.94E-10 | 3.04E-08 |
| 67 | GO:0060745 | Biological Process | mammary gland branching involved in pregnancy | 4 | 8.45E-10 | 4.25E-08 |
| 68 | GO:0007169 | Biological Process | transmembrane receptor protein tyrosine kinase signaling pathway | 14 | 1.25E-09 | 6.16E-08 |
| 69 | GO:0009628 | Biological Process | response to abiotic stimulus | 16 | 1.43E-09 | 6.97E-08 |
| 70 | GO:0032559 | Molecular Function | adenyl ribonucleotide binding | 22 | 1.48E-09 | 7.37E-08 |
| 71 | GO:0030554 | Molecular Function | adenyl nucleotide binding | 22 | 1.73E-09 | 7.66E-08 |
| 72 | GO:0004714 | Molecular Function | transmembrane receptor protein tyrosine kinase activity | 8 | 2.10E-09 | 8.35E-08 |
| 73 | GO:0003824 | Molecular Function | catalytic activity | 45 | 2.34E-09 | 8.45E-08 |
| 74 | GO:0002682 | Biological Process | regulation of immune system process | 19 | 2.14E-09 | 1.02E-07 |
| 75 | GO:0010604 | Biological Process | positive regulation of macromolecule metabolic process | 30 | 2.21E-09 | 1.04E-07 |
| 76 | GO:0008270 | Molecular Function | zinc ion binding | 16 | 4.11E-09 | 1.28E-07 |
| 77 | GO:0004871 | Molecular Function | signal transducer activity | 27 | 4.17E-09 | 1.28E-07 |
| 78 | GO:0018105 | Biological Process | peptidyl-serine phosphorylation | 11 | 2.90E-09 | 1.35E-07 |
| 79 | GO:0032501 | Biological Process | multicellular organismal process | 45 | 3.94E-09 | 1.78E-07 |
| 80 | GO:0009605 | Biological Process | response to external stimulus | 24 | 3.96E-09 | 1.78E-07 |
| 81 | GO:0071407 | Biological Process | cellular response to organic cyclic compound | 12 | 4.13E-09 | 1.83E-07 |
| 82 | GO:0005524 | Molecular Function | ATP binding | 21 | 6.44E-09 | 1.83E-07 |
| 83 | GO:0043066 | Biological Process | negative regulation of apoptotic process | 15 | 4.43E-09 | 1.94E-07 |
| 84 | GO:0048583 | Biological Process | regulation of response to stimulus | 31 | 4.74E-09 | 2.04E-07 |
| 85 | GO:0004715 | Molecular Function | non-membrane spanning protein tyrosine kinase activity | 6 | 7.76E-09 | 2.05E-07 |
| 86 | GO:0019199 | Molecular Function | transmembrane receptor protein kinase activity | 8 | 8.26E-09 | 2.05E-07 |
| 87 | GO:0032270 | Biological Process | positive regulation of cellular protein metabolic process | 20 | 4.96E-09 | 2.11E-07 |
| 88 | GO:0009611 | Biological Process | response to wounding | 14 | 5.58E-09 | 2.34E-07 |
| 89 | GO:0004713 | Molecular Function | protein tyrosine kinase activity | 8 | 1.03E-08 | 2.40E-07 |
| 90 | GO:0046914 | Molecular Function | transition metal ion binding | 18 | 1.14E-08 | 2.52E-07 |
| 91 | GO:0044093 | Biological Process | positive regulation of molecular function | 20 | 7.38E-09 | 3.05E-07 |
| 92 | GO:0044238 | Biological Process | primary metabolic process | 56 | 7.81E-09 | 3.18E-07 |
| 93 | GO:0006952 | Biological Process | defense response | 19 | 8.53E-09 | 3.43E-07 |
| 94 | GO:0071704 | Biological Process | organic substance metabolic process | 57 | 8.77E-09 | 3.48E-07 |
| 95 | GO:0044267 | Biological Process | cellular protein metabolic process | 38 | 8.96E-09 | 3.51E-07 |
| 96 | GO:0051704 | Biological Process | multi-organism process | 22 | 9.31E-09 | 3.60E-07 |
| 97 | GO:0004175 | Molecular Function | endopeptidase activity | 13 | 1.80E-08 | 3.77E-07 |
| 98 | GO:0019899 | Molecular Function | enzyme binding | 22 | 1.96E-08 | 3.90E-07 |
| 99 | GO:0031325 | Biological Process | positive regulation of cellular metabolic process | 29 | 1.06E-08 | 4.03E-07 |
| 100 | GO:0060444 | Biological Process | branching involved in mammary gland duct morphogenesis | 5 | 1.14E-08 | 4.30E-07 |

**Table S3 KEGG enrichment analysis of Network pharmacy (TOP100)**

|  | **ID** | **Pathways** | **Class** | **Num** | **Ratio** | **P-value** | **Q-value** |
| --- | --- | --- | --- | --- | --- | --- | --- |
| 1 | ko05200 | Pathways in cancer | Human Diseases | 18 | 0.032 | 8.60E-09 | 1.38E-06 |
| 2 | ko05215 | Prostate cancer | Human Diseases | 9 | 0.088 | 1.45E-08 | 1.38E-06 |
| 3 | ko01521 | EGFR tyrosine kinase inhibitor resistance | Human Diseases | 8 | 0.091 | 7.76E-08 | 4.92E-06 |
| 4 | ko05224 | Breast cancer | Human Diseases | 9 | 0.059 | 4.64E-07 | 2.20E-05 |
| 5 | ko05167 | Kaposi sarcoma-associated herpesvirus infection | Human Diseases | 10 | 0.049 | 5.83E-07 | 2.21E-05 |
| 6 | ko04917 | Prolactin signaling pathway | Organismal Systems | 7 | 0.083 | 9.75E-07 | 3.09E-05 |
| 7 | ko01522 | Endocrine resistance | Human Diseases | 7 | 0.072 | 2.59E-06 | 5.58E-05 |
| 8 | ko04062 | Chemokine signaling pathway | Organismal Systems | 9 | 0.048 | 2.75E-06 | 5.58E-05 |
| 9 | ko05163 | Human cytomegalovirus infection | Human Diseases | 10 | 0.041 | 2.86E-06 | 5.58E-05 |
| 10 | ko04915 | Estrogen signaling pathway | Organismal Systems | 8 | 0.057 | 2.93E-06 | 5.58E-05 |
| 11 | ko04217 | Necroptosis | Cellular Processes | 9 | 0.047 | 3.27E-06 | 5.65E-05 |
| 12 | ko05235 | PD-L1 expression and PD-1 checkpoint pathway in cancer | Human Diseases | 8 | 0.055 | 3.81E-06 | 6.03E-05 |
| 13 | ko04625 | C-type lectin receptor signaling pathway | Organismal Systems | 7 | 0.066 | 4.70E-06 | 6.87E-05 |
| 14 | ko04750 | Inflammatory mediator regulation of TRP channels | Organismal Systems | 7 | 0.065 | 5.33E-06 | 7.23E-05 |
| 15 | ko05033 | Nicotine addiction | Human Diseases | 5 | 0.122 | 5.81E-06 | 7.35E-05 |
| 16 | ko05145 | Toxoplasmosis | Human Diseases | 7 | 0.057 | 1.19E-05 | 1.37E-04 |
| 17 | ko05161 | Hepatitis B | Human Diseases | 8 | 0.047 | 1.23E-05 | 1.37E-04 |
| 18 | ko04659 | Th17 cell differentiation | Organismal Systems | 8 | 0.046 | 1.33E-05 | 1.41E-04 |
| 19 | ko04611 | Platelet activation | Organismal Systems | 7 | 0.055 | 1.55E-05 | 1.55E-04 |
| 20 | ko05135 | Yersinia infection | Human Diseases | 8 | 0.043 | 2.44E-05 | 2.32E-04 |
| 21 | ko04914 | Progesterone-mediated oocyte maturation | Organismal Systems | 6 | 0.064 | 2.87E-05 | 2.59E-04 |
| 22 | ko04550 | Signaling pathways regulating pluripotency of stem cells | Cellular Processes | 7 | 0.049 | 3.20E-05 | 2.77E-04 |
| 23 | ko04510 | Focal adhesion | Cellular Processes | 8 | 0.04 | 3.94E-05 | 3.26E-04 |
| 24 | ko04658 | Th1 and Th2 cell differentiation | Organismal Systems | 7 | 0.045 | 5.39E-05 | 4.26E-04 |
| 25 | ko04657 | IL-17 signaling pathway | Organismal Systems | 6 | 0.056 | 5.97E-05 | 4.54E-04 |
| 26 | ko05205 | Proteoglycans in cancer | Human Diseases | 8 | 0.037 | 7.01E-05 | 5.12E-04 |
| 27 | ko04931 | Insulin resistance | Human Diseases | 6 | 0.053 | 8.10E-05 | 5.70E-04 |
| 28 | ko04071 | Sphingolipid signaling pathway | Environmental Information Processing | 6 | 0.049 | 0.000124 | 8.41E-04 |
| 29 | ko05164 | Influenza A | Human Diseases | 7 | 0.037 | 0.000182 | 1.19E-03 |
| 30 | ko04068 | FoxO signaling pathway | Environmental Information Processing | 6 | 0.043 | 0.000243 | 1.54E-03 |
| 31 | ko04151 | PI3K-Akt signaling pathway | Environmental Information Processing | 10 | 0.024 | 0.000262 | 1.61E-03 |
| 32 | ko04270 | Vascular smooth muscle contraction | Organismal Systems | 6 | 0.043 | 0.000273 | 1.62E-03 |
| 33 | ko04610 | Complement and coagulation cascades | Organismal Systems | 5 | 0.053 | 0.000328 | 1.89E-03 |
| 34 | ko04621 | NOD-like receptor signaling pathway | Organismal Systems | 7 | 0.034 | 0.00034 | 1.90E-03 |
| 35 | ko05152 | Tuberculosis | Human Diseases | 7 | 0.033 | 0.000415 | 2.25E-03 |
| 36 | ko04370 | VEGF signaling pathway | Environmental Information Processing | 4 | 0.071 | 0.000441 | 2.33E-03 |
| 37 | ko04933 | AGE-RAGE signaling pathway in diabetic complications | Human Diseases | 5 | 0.047 | 0.000571 | 2.93E-03 |
| 38 | ko05213 | Endometrial cancer | Human Diseases | 4 | 0.066 | 0.000612 | 3.06E-03 |
| 39 | ko05160 | Hepatitis C | Human Diseases | 6 | 0.036 | 0.000718 | 3.50E-03 |
| 40 | ko04935 | Growth hormone synthesis, secretion and action | Organismal Systems | 5 | 0.043 | 0.000894 | 4.25E-03 |
| 41 | ko05132 | Salmonella infection | Human Diseases | 7 | 0.028 | 0.000951 | 4.41E-03 |
| 42 | ko05230 | Central carbon metabolism in cancer | Human Diseases | 4 | 0.058 | 0.000975 | 4.41E-03 |
| 43 | ko04722 | Neurotrophin signaling pathway | Organismal Systems | 5 | 0.041 | 0.001079 | 4.77E-03 |
| 44 | Ko04630 | JAK-STAT signaling pathway | Environmental Information Processing | 6 | 0.032 | 0.001183 | 5.11E-03 |
| 45 | ko04380 | Osteoclast differentiation | Organismal Systems | 5 | 0.039 | 0.001385 | 5.78E-03 |
| 46 | ko05218 | Melanoma | Human Diseases | 4 | 0.053 | 0.0014 | 5.78E-03 |
| 47 | ko04926 | Relaxin signaling pathway | Organismal Systems | 5 | 0.038 | 0.001483 | 5.99E-03 |
| 48 | ko05203 | Viral carcinogenesis | Human Diseases | 7 | 0.026 | 0.001563 | 6.19E-03 |
| 49 | ko05214 | Glioma | Human Diseases | 4 | 0.051 | 0.001616 | 6.27E-03 |
| 50 | ko04114 | Oocyte meiosis | Cellular Processes | 5 | 0.037 | 0.001694 | 6.44E-03 |
| 51 | ko04080 | Neuroactive ligand-receptor interaction | Environmental Information Processing | 8 | 0.022 | 0.001936 | 7.21E-03 |
| 52 | ko05162 | Measles | Human Diseases | 6 | 0.029 | 0.002195 | 8.02E-03 |
| 53 | ko04012 | ErbB signaling pathway | Environmental Information Processing | 4 | 0.046 | 0.002304 | 8.26E-03 |
| 54 | ko04115 | p53 signaling pathway | Cellular Processes | 4 | 0.045 | 0.002402 | 8.45E-03 |
| 55 | ko04912 | GnRH signaling pathway | Organismal Systems | 4 | 0.043 | 0.002824 | 9.76E-03 |
| 56 | ko04727 | GABAergic synapse | Organismal Systems | 4 | 0.042 | 0.003172 | 1.08E-02 |
| 57 | ko05418 | Fluid shear stress and atherosclerosis | Human Diseases | 5 | 0.032 | 0.003271 | 1.08E-02 |
| 58 | ko00590 | Arachidonic acid metabolism | Metabolism | 4 | 0.042 | 0.003294 | 1.08E-02 |
| 59 | ko05032 | Morphine addiction | Human Diseases | 4 | 0.04 | 0.00368 | 1.19E-02 |
| 60 | ko04660 | T cell receptor signaling pathway | Organismal Systems | 5 | 0.031 | 0.003841 | 1.22E-02 |
| 61 | ko04928 | Parathyroid hormone synthesis, secretion and action | Organismal Systems | 4 | 0.037 | 0.004857 | 1.51E-02 |
| 62 | ko04218 | Cellular senescence | Cellular Processes | 5 | 0.028 | 0.005706 | 1.75E-02 |
| 63 | ko04725 | Cholinergic synapse | Organismal Systems | 4 | 0.035 | 0.006075 | 1.83E-02 |
| 64 | ko04919 | Thyroid hormone signaling pathway | Organismal Systems | 4 | 0.034 | 0.006457 | 1.92E-02 |
| 65 | ko04210 | Apoptosis | Cellular Processes | 5 | 0.027 | 0.006696 | 1.96E-02 |
| 66 | ko04668 | TNF signaling pathway | Environmental Information Processing | 4 | 0.033 | 0.007919 | 2.25E-02 |
| 67 | ko04726 | Serotonergic synapse | Organismal Systems | 4 | 0.033 | 0.007919 | 2.25E-02 |
| 68 | ko04015 | Rap1 signaling pathway | Environmental Information Processing | 6 | 0.022 | 0.008166 | 2.25E-02 |
| 69 | ko05165 | Human papillomavirus infection | Human Diseases | 7 | 0.019 | 0.008193 | 2.25E-02 |
| 70 | ko05340 | Primary immunodeficiency | Human Diseases | 3 | 0.046 | 0.008303 | 2.25E-02 |
| 71 | ko05221 | Acute myeloid leukemia | Human Diseases | 3 | 0.045 | 0.008659 | 2.32E-02 |
| 72 | ko05223 | Non-small cell lung cancer | Human Diseases | 3 | 0.044 | 0.009398 | 2.48E-02 |
| 73 | ko04920 | Adipocytokine signaling pathway | Organismal Systems | 3 | 0.041 | 0.01183 | 3.08E-02 |
| 74 | ko04010 | MAPK signaling pathway | Environmental Information Processing | 6 | 0.02 | 0.012599 | 3.23E-02 |
| 75 | ko05169 | Epstein-Barr virus infection | Human Diseases | 6 | 0.02 | 0.013179 | 3.34E-02 |
| 76 | ko04810 | Regulation of actin cytoskeleton | Cellular Processes | 5 | 0.022 | 0.014233 | 3.56E-02 |
| 77 | ko05212 | Pancreatic cancer | Human Diseases | 3 | 0.037 | 0.014594 | 3.60E-02 |
| 78 | ko04140 | Autophagy - animal | Cellular Processes | 4 | 0.026 | 0.016613 | 4.05E-02 |
| 79 | ko04921 | Oxytocin signaling pathway | Organismal Systems | 4 | 0.026 | 0.017344 | 4.17E-02 |
| 80 | ko05210 | Colorectal cancer | Human Diseases | 3 | 0.034 | 0.019371 | 4.60E-02 |
| 81 | ko04540 | Gap junction | Cellular Processes | 3 | 0.032 | 0.02174 | 5.10E-02 |
| 82 | ko04723 | Retrograde endocannabinoid signaling | Organismal Systems | 4 | 0.023 | 0.024369 | 5.65E-02 |
| 83 | ko04072 | Phospholipase D signaling pathway | Environmental Information Processing | 4 | 0.023 | 0.024829 | 5.67E-02 |
| 84 | ko00232 | Caffeine metabolism | Metabolism | 1 | 0.25 | 0.02545 | 5.67E-02 |
| 85 | ko04150 | mTOR signaling pathway | Environmental Information Processing | 4 | 0.023 | 0.025764 | 5.67E-02 |
| 86 | ko05225 | Hepatocellular carcinoma | Human Diseases | 4 | 0.023 | 0.025764 | 5.67E-02 |
| 87 | ko04022 | cGMP-PKG signaling pathway | Environmental Information Processing | 4 | 0.023 | 0.02624 | 5.67E-02 |
| 88 | ko05206 | MicroRNAs in cancer | Human Diseases | 4 | 0.023 | 0.02624 | 5.67E-02 |
| 89 | ko05140 | Leishmaniasis | Human Diseases | 3 | 0.029 | 0.028328 | 6.05E-02 |
| 90 | ko04620 | Toll-like receptor signaling pathway | Organismal Systems | 3 | 0.029 | 0.02976 | 6.28E-02 |
| 91 | ko04360 | Axon guidance | Organismal Systems | 4 | 0.022 | 0.030235 | 6.31E-02 |
| 92 | ko00591 | Linoleic acid metabolism | Metabolism | 2 | 0.044 | 0.033667 | 6.95E-02 |
| 93 | ko04530 | Tight junction | Cellular Processes | 4 | 0.021 | 0.03514 | 7.18E-02 |
| 94 | ko05219 | Bladder cancer | Human Diseases | 2 | 0.043 | 0.036463 | 7.37E-02 |
| 95 | ko04666 | Fc gamma R-mediated phagocytosis | Organismal Systems | 3 | 0.026 | 0.037483 | 7.50E-02 |
| 96 | ko04930 | Type II diabetes mellitus | Human Diseases | 2 | 0.042 | 0.037894 | 7.50E-02 |
| 97 | ko04066 | HIF-1 signaling pathway | Environmental Information Processing | 3 | 0.025 | 0.042563 | 8.33E-02 |
| 98 | ko05170 | Human immunodeficiency virus 1 infection | Human Diseases | 5 | 0.017 | 0.042978 | 8.33E-02 |
| 99 | ko04670 | Leukocyte transendothelial migration | Organismal Systems | 3 | 0.024 | 0.04433 | 8.51E-02 |
| 100 | ko04961 | Endocrine and other factor-regulated calcium reabsorption | Organismal Systems | 2 | 0.038 | 0.045363 | 8.62E-02 |
